# Contrast versus identity encoding in the face image follow distinct orientation selectivity profiles

**DOI:** 10.1371/journal.pone.0229185

**Published:** 2020-03-18

**Authors:** Christianne Jacobs, Kirsten Petras, Pieter Moors, Valerie Goffaux

**Affiliations:** 1 Faculty of Psychology and Educational Sciences, Research Institute for Psychological Science (IPSY), UC Louvain, Louvain-la-Neuve, Belgium; 2 Department of Cognitive Neuroscience, Faculty of Psychology and Neuroscience, Maastricht University, Maastricht, the Netherlands; 3 Department of Brain and Cognition, Laboratory of Experimental Psychology, KU Leuven, Leuven, Belgium; 4 Institute of Neuroscience (IoNS), UC Louvain, Louvain-la-Neuve, Belgium; University of Toyama, JAPAN

## Abstract

Orientation selectivity is a fundamental property of primary visual encoding. High-level processing stages also show some form of orientation dependence, with face identification preferentially relying on horizontally-oriented information. How high-level orientation tuning emerges from primary orientation biases is unclear. In the same group of participants, we derived the orientation selectivity profile at primary and high-level visual processing stages using a contrast detection and an identity matching task. To capture the orientation selectivity profile, we calculated the difference in performance between all tested orientations (0, 45, 90, and 135°) for each task and for upright and inverted faces, separately. Primary orientation selectivity was characterized by higher sensitivity to oblique as compared to cardinal orientations. The orientation profile of face identification showed superior horizontal sensitivity to face identity. In each task, performance with upright and inverted faces projected onto qualitatively similar *a priori* models of orientation selectivity. Yet the fact that the orientation selectivity profiles of contrast detection in upright and inverted faces correlated significantly while such correlation was absent for identification indicates a progressive dissociation of orientation selectivity profiles from primary to high-level stages of orientation encoding. Bayesian analyses further indicate a lack of correlation between the orientation selectivity profiles in the contrast detection and face identification tasks, for upright and inverted faces. From these findings, we conclude that orientation selectivity shows distinct profiles at primary and high-level stages of face processing and that a transformation must occur from general cardinal attenuation when processing basic properties of the face image to horizontal tuning when encoding more complex properties such as identity.

## Introduction

Before we can make sense of the light the world projects onto our retina, the induced neural signals need to undergo extensive processing in the cortex, which they reach mainly through the primary visual cortex (V1). V1 neurons respond to contrast at selective orientations. Orientation selectivity is the hallmark computational principle of primary visual processing in mammals [1–3].

Despite being more complex than the features encoded in V1, the features encoded at higher-level processing stages (e.g., shape, curvature, category membership, face identity) still show some form of orientation dependence [4–9]. Consistent evidence indicates that the processing of face identity is tuned to horizontally oriented input; it declines progressively as visual input is oriented away from horizontal, and reaches its minimum when based on vertically-oriented cues. This horizontal tuning is already present in infants and strengthens until adulthood [10–12]. We further showed that horizontally-filtered face images trigger the largest response in the Fusiform Face Area (FFA), a high-level visual region responding preferentially to faces ([13]; see [14,15] for EEG evidence of a horizontal dependence of face-specialized neural responses at a latency corresponding to high-level processing stages). We know little about the emergence of such high-level manifestations of orientation selectivity from the primary encoding of orientation. This question is far from trivial and extends beyond the domain of face perception [16].

Evidence indicates that the primary encoding of orientation adopts drastically different profiles depending on stimulus properties. In studies using simple, single orientation stimuli like gratings or Gabor stimuli, humans are typically most sensitive to contrast at horizontal and vertical orientations, a preference generally referred to as the oblique effect [17–24]. However, this orientation selectivity profile reverses when measured with images containing a wider range of orientations (i.e., broadband images [25–29], see [30] for a similar finding with narrowband gratings). In broadband images, human observers indeed detect contrast increments best in oblique, worse in horizontal, and intermediate in the vertical orientation band, a pattern referred to as the horizontal effect. This horizontal effect presumably reflects suppressive gain control mechanisms in V1 [25,26,31,32]. Horizontal energy typically predominates in natural scene images due, in part, to the presence of the horizon and the foreshortening of the ground plane in the vertical direction [24,28]. Suppressive mechanisms would act to increase the salience of the off-horizontal elements in natural broadband input.

There is also evidence for higher-level influences on the primary encoding of local orientation. For example, the sensitivity to the orientation of a local Gabor embedded in a complex scene is influenced by the figure-ground organization of the scene at mid- or high-level processing stages more than by the low-level contrast properties at the locus of Gabor insertion [33,34].

Akin to natural scenes, face images are broadband and contain disproportionately more contrast in the horizontal range [4,6,8,35]. Past studies have investigated the sensitivity to identity across orientations; but none of them has investigated the orientation selectivity of the processing of primary properties of the face stimulus. It therefore remains unclear how the horizontal tuning of high-level face identification emerges from primary orientation biases.

In the present study, we characterized the orientation selectivity profile of primary contrast detection in face images and examined its link to the horizontal tuning of high-level face identification. Our approach offers a unique opportunity to address the relationship between primary and high-level orientation biases.

Several lines of evidence indicate that the horizontal tuning to face identity does not arise at primary orientation encoding stages. Firstly, inverting the face image in the picture plane significantly reduces the horizontal dependence of identification performance [5–7,36]. Inversion interferes with the high-level processing of the face image, but leaves its orientation content (and average response in V1; see e.g. 13) unchanged. If the primary orientation-selective encoding of the face stimulus were driving the horizontal tuning to face identity, inverted face images should also be matched best in the horizontal range. The observation that, in contrast to high-level FFA, V1 shows a decreased BOLD signal in response to any horizontally filtered image (be it upright, inverted, or scrambled) as compared to other orientations [13], further contradicts the idea that horizontal tuning directly derives from primary orientation biases.

However, this issue can only be addressed by systematically comparing the high-level orientation dependence of face identification to the tuning properties as observed with a task that specifically taps into the primary encoding of oriented contrast. To do so we used two experimental paradigms conventionally used to investigate the primary orientation biases in contrast detection [27–29] and the horizontal tuning of high-level face identification [5,6,35,36], respectively. In the contrast detection task, we asked participants to detect the presence of a contrast increment of a particular orientation in upright and inverted images of faces. In the face identification task, participants matched the identity of orientation-filtered faces.

Stimulus and task parameters differed across experiments. Nevertheless, we transformed the data of each task into a similar format, which allowed us to compare orientation selectivity profiles across tasks. We captured the individual orientation selectivity profile across all tested orientations by expressing individual data as a matrix or vector in which each of the cells represents the difference of performance between two orientation conditions. These relative differences are well suited for the exploration of orientation selectivity patterns, as they are not susceptible to any absolute sensitivity differences potentially caused by differing stimulus or task parameters. In addition, our analyses take the pattern across all orientation conditions into consideration rather than simply detecting any difference between two orientation conditions (like classical analysis-of-variance).

We then compared these individual difference vectors of both tasks to *a priori* models of orientation selectivity. We included 1) a model of the cardinal effect, 2) a horizontal effect model, and 3) a ‘horizontal is special’-model. Since face images are complex and broadband, we expected the contrast sensitivity pattern across orientations to resemble that acquired previously with broadband stimuli. We thus hypothesized to find attenuated contrast sensitivity to the cardinal, and especially horizontal, orientations as opposed to the oblique orientations (i.e. horizontal\cardinal effect). This prediction is also in accordance with the reduced neural response to horizontally filtered images in V1 [13,30].

Next, we correlated the orientation selectivity patterns of the contrast detection task with that of the face identification task in order to test whether orientation selectivity across tasks is related. Despite potentially opposite directions in primary and high-level orientation biases at the group level, they could still be functionally linked such that the most horizontally-tuned face identifiers would also be most sensitive to horizontal contrast in the primary task. We used correlation analyses to evaluate the relationship between individuals’ orientation response profiles between the contrast detection and face identification tasks. In the case of a functional link between primary and high-level orientation dependencies, we further hypothesized to observe a difference in the orientation selectivity profiles in the contrast detection task for upright and inverted faces. Considering past findings that high-level representations influence the primary processing of oriented contrast [33,34], higher-level feedback about the presence of an upright face in the image plausibly modulates the orientation tuning towards the diagnostic orientation already at the primary stages of processing and reduces the horizontal effect compared to when an inverted face is processed.

## Methods

### Participants

Twenty-four participants (18 females, mean age 23.3y SD 1.76y) completed a contrast detection task on three categories of face stimuli: upright, inverted, and phase-scrambled faces, and an identification task on upright and inverted faces. A subset of this group (N = 10, 6 females, mean age 23.6y, SD 2.5y) also completed the contrast detection task on intact and phase-scrambled natural scenes in order to allow for a comparison between faces and natural scenes.

All participants scored within the normal range on computerized tests of visual acuity (Landolt C task) and astigmatism (standard astigmatism charts). They provided written informed consent at the start of the experiment, and received a monetary compensation of 8 euro/h after the experiment was completed. The experimental protocol adhered to the Declaration of Helsinki and was approved by the local ethical committee (Psychological Sciences Research Institute, UC Louvain).

### Stimuli

**Fig 1:**
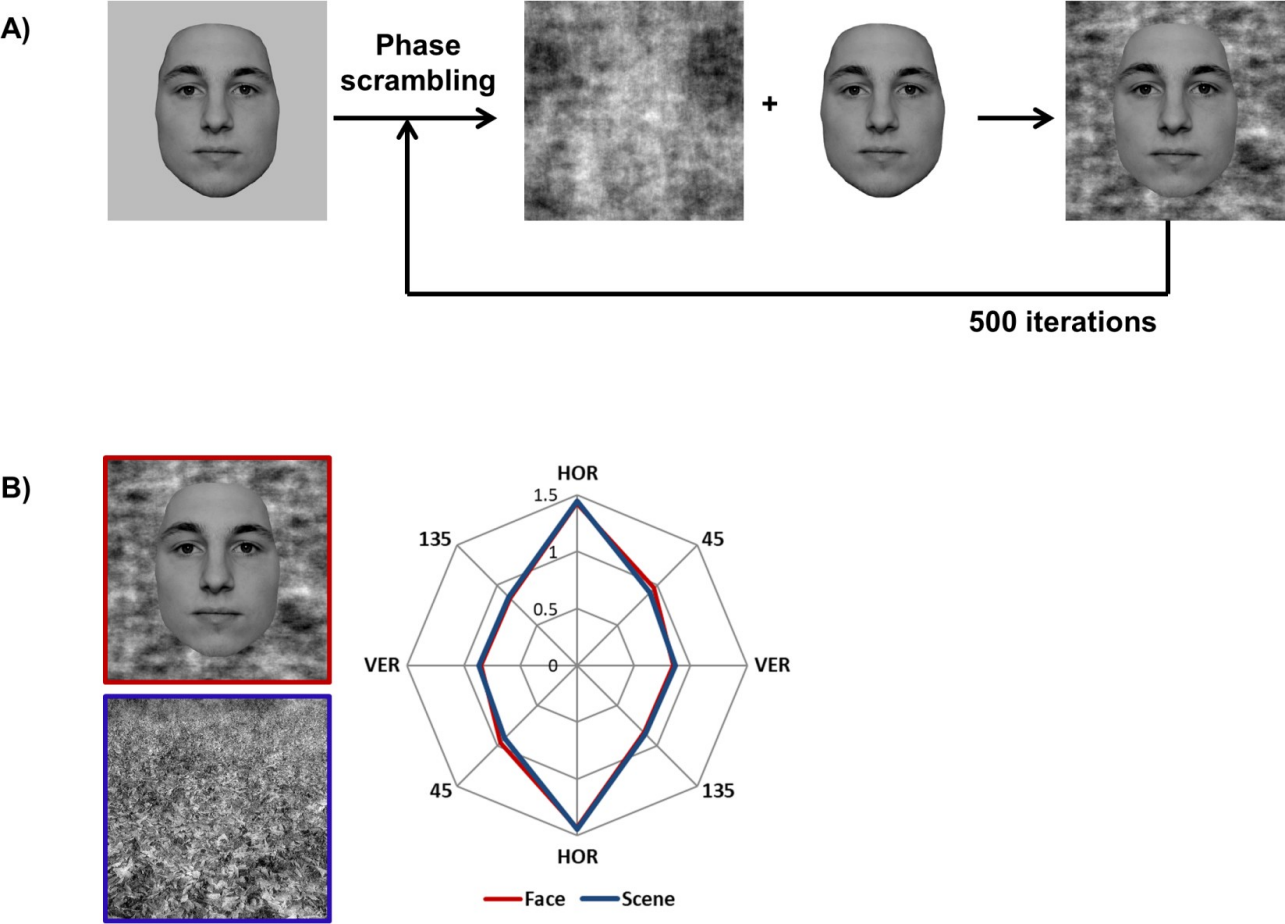
A) Face stimulus creation. The face image was iteratively phase-scrambled, re-combined with non-scrambled face pixels, and scrambled again (number of iterations was 500). B) Matching of face and scene images. The relative amplitude at vertical, 45° oblique, horizontal, and 135° oblique angles was compared across the face and scene categories. Through method of least squares the scene image with the best-matching amplitude profile was selected to be included in the stimulus set. Left panel: example of matched face and scene stimulus. Right panel: the amplitude profile plots (in arbitrary units) for these two images. The red line represents the face image, whereas the blue line represents the scene image.

We created the experimental stimuli for our contrast detection experiment in the same manner as [28]. Face stimuli consisted of 40 greyscale images of male and female faces cropped to remove hair, neck, ears, clothes etc. and pasted onto a uniform grey background. To ensure that the image background shared the face’s spectral properties, the whole image was phase-scrambled in Fourier space, the original face was pasted back onto the now-scrambled background, and then the entire image was phase-scrambled again [37]. This procedure of pasting the face-related pixels back onto the background and phase-scrambling the resultant image was repeated 500 times (Fig 1A). By making the spectral properties of face and background pixels more similar, this procedure prevented the stimuli in scrambled conditions from being contaminated by the uniform background.

Natural scene images were selected to match face images for orientation content. The orientation profile, i.e. the relative amplitude across four orientation bands centered on 0° (i.e. vertical), 45°, 90° (i.e. horizontal), and 135° with a width of 45° for each of the face images was calculated, as well as for each of the images in a database of 2000 natural scene images (courtesy of Bruce Hansen). Using the method of least squares (i.e. minimizing the sum of squared errors), best fits with each individual face were computed and the corresponding scene images were included in the experiment (Fig 1B). If two faces shared a best-matched scene, for one of the faces, the scene with the second smallest squared error was added to the stimulus set. Images were not matched for spectral slope; therefore spectral slopes were steeper for faces as compared to scenes (Fig 1B, right panel), because natural images typically have a spectral slope that can be described by a 1/f function, whereas face images are better characterized by a 1/f^2^ spectral slope [38–40]. For the participants who completed the scene conditions, phase-scrambled version of the scenes were included, in order to be able to test for spectral slope as an explanation for any differences observed between scenes and faces.

In order to replicate the numbers of presentations of each exemplar in [28], 20 from the set of 40 matched face-scene combination were selected randomly for each participant and these pairs formed their individual stimulus set, respectively. Inverted versions of the face stimuli, and versions, which were phase-scrambled in Fourier space, were also included.

After image selection, we created isotropic versions of all stimuli, meaning that all orientations in the image share the same amount of energy. This was achieved by replacing in the Fourier domain the individual amplitudes for each orientation and spatial frequency with the average value across orientations within that same spatial frequency (i.e. rotational average). In order to create orientation increments (i.e. an amplification of the energy in a particular orientation band [27,28], the amplitude spectrum was multiplied with a filter centered on the 0°, 45°, 90° or 135° orientation with a bandwidth of 45° including all spatial frequencies [28]. The weighing function dropped of linearly from maximal at center orientation to zero at the outer orientation boundaries (i.e. triangular filter). The magnitude of the amplitude increase was set to 30% of the original amplitude [28]. Finally, these modified amplitude spectra resulted were inverse Fourier-transformed. We ensured that the stimulus procedures altogether did not lead to pixel clipping in over 0.1% of pixels.

For the face identification task, we adapted the design of several studies previously run in our laboratory [5,6,35,36]. We presented the same upright faces and inverted faces as in the contrast detection task. They had been *a priori* filtered to contain information in a limited orientation band. Prior to filtering, the luminance of each face image was first normalized to obtain a mean of 0 and a root-mean square (RMS) contrast of 1. Filtered stimuli were generated by Fast Fourier transforming the normalized image and multiplying the Fourier energy with orientation filters allowing all spatial frequencies to pass but had a wrapped Gaussian energy profile in the orientation domain, centered on either vertical, 45° oblique, horizontal, or 135° oblique orientations with a particular bandwidth specified by the standard deviation parameter (cf e.g., 4,5). We used a standard deviation of 14° to agree with the orientation properties of neurons in the primary visual cortex (e.g., [41,42]). For sake of comparison with the contrast increment stimuli, the filtered face images were pasted onto the background with the spectral slope properties of the original image (i.e., the phase-scrambled version of the original face image).

**Fig 2:**
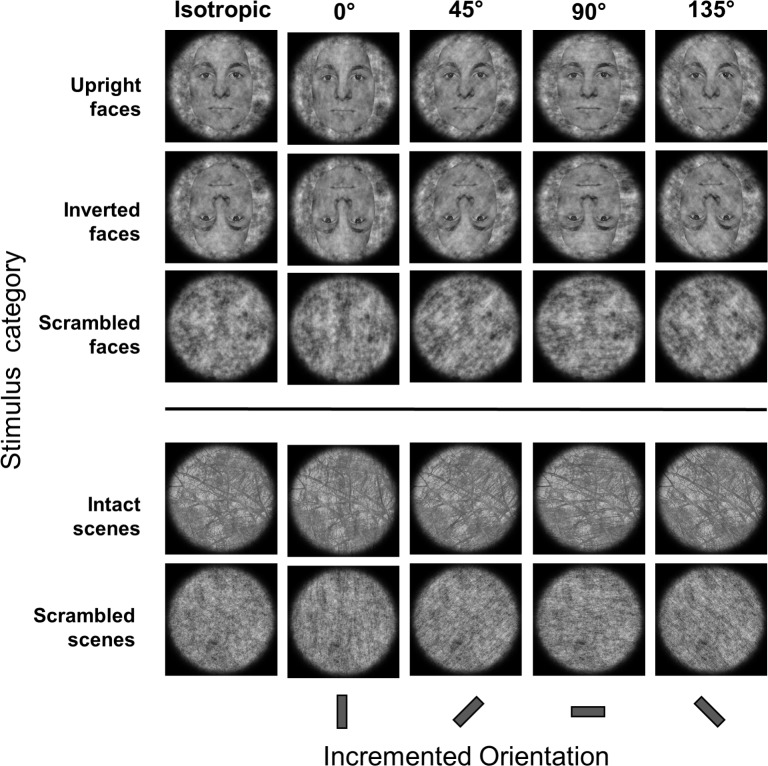
Example stimuli. Stimuli of each of the five different stimulus categories were first made isotropic, i.e. all orientations carry equal amplitude. An orientation increment was created by increasing the relative amplitude in a 45°-wide band centered on one of four possible orientations: vertical, 45° oblique, horizontal, and 135° oblique. For illustration purposes, the intensity of the increments in the figure is magnified. Participants were to indicate on each trial whether the presented stimulus was isotropic or contained an orientation increment.

In both experiments, all images were equalized for luminance (0.5) and RMS contrast (0.1) and were combined with a circular edge-blurred aperture to avoid interference on processing due to border orientation (Fig 2). Final image size was 10.5° visual angle. Image manipulations were executed with custom-written scripts in Matlab 2014a (Mathworks Inc, Natick, MA). All stimuli were presented on a Viewpixx monitor (VPixx Technologies Inc., Saint-Bruno, Canada) with a 1920 x 1080 pixel resolution and a 70Hz refresh rate. Scanning back-light option was switched on, and the maximal luminance set to 80 cd/m^2^. At the start of the experiment lighting was switched off and the testing area of the lab was closed off separately with light-draining black curtains.

### Tasks and procedure

Participants visited the laboratory on seven (N = 14) or eleven (N = 10) different occasions, with at least a half hour of rest in between sessions. Each session lasted about 45 minutes. In the first sessions they performed a *contrast detection task*, and in the last session a *face identification task*. This order was fixed, so that the amount of passive exposure to the face stimuli in the contrast detection task had been equal for all participants before starting the face identification task. At the beginning of each session, the participants were seated comfortably at 70cm distance from the monitor with their head resting in a chinrest.

#### Contrast detection task

**Fig 3:**
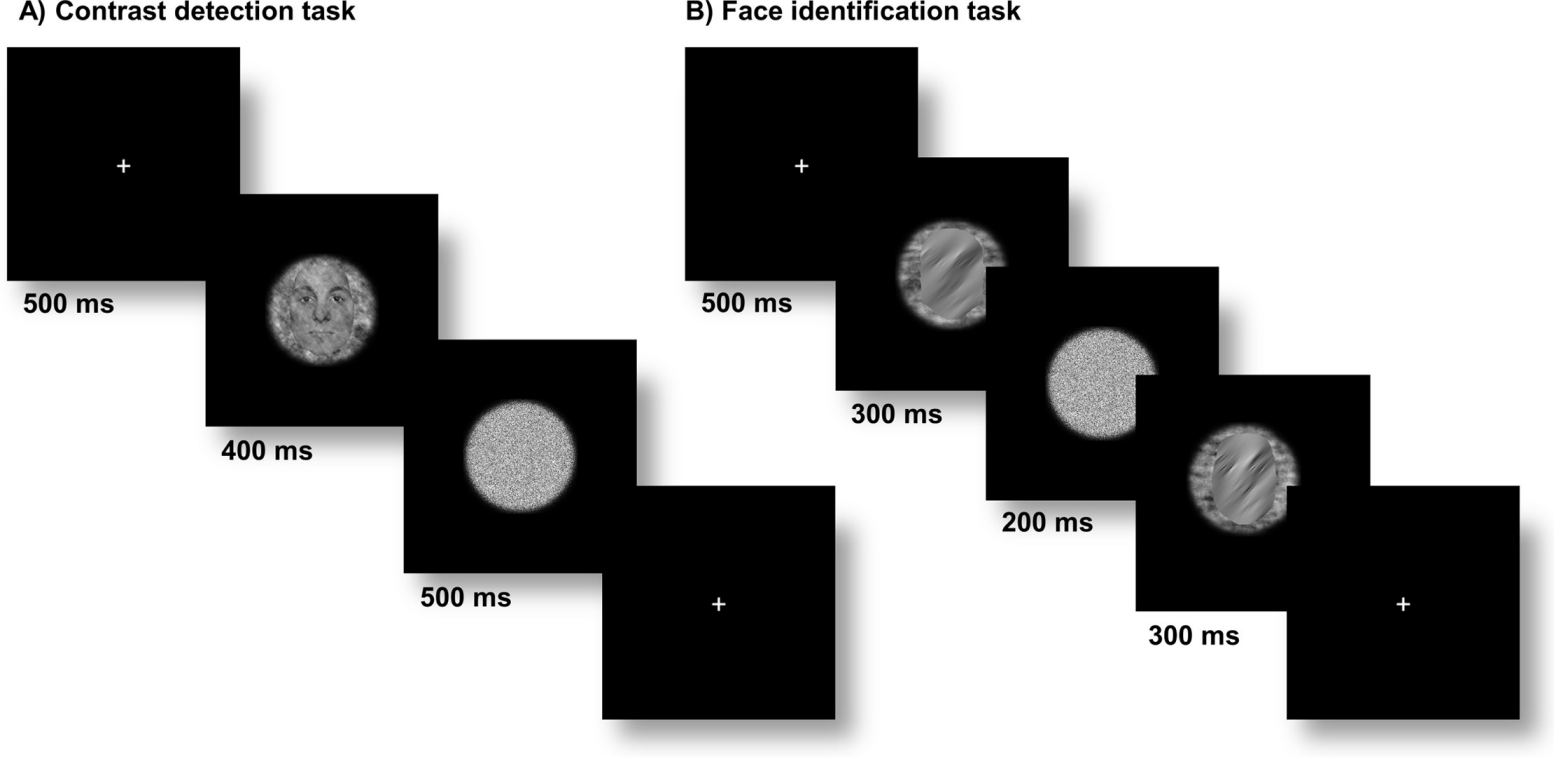
Example trials for all tasks. A) Contrast detection task. Each trial started with a 500 ms fixation period. A target stimulus (here: upright face) was presented for 400 ms followed by a 500 ms random noise mask. The participants indicated by button press whether they perceived an increment of a particular orientation in the target image (here: present). B) High-level face identification task. Each trial started with a 500 ms fixation period. An initial face stimulus, filtered to contain information in a limited orientation band (here: centered on 135° oblique) was presented for 300 ms. After a brief noise mask was shown for 200 ms, a second face stimulus was presented for 300 ms. The participants indicated by button press whether the identity of the two faces was identical or different (here: different). The location of the second stimulus was varied slightly relative to the first one, in order to reduce the influence of local low-level visual properties.

Each session consisted of four blocks, one block per orientation. At the start of each block, participants were instructed about the orientation at which they were requested to detect the contrast increment in the upcoming block. The order of the blocks was randomized. Each block contained 160 trials. Every trial started with a 500ms fixation period, after which the target stimulus appeared on screen for 400ms, followed by a white noise mask presented for 500ms (Fig 3A). The position of both target stimulus and mask was jittered in both x and y directions with a maximum of 20 pixels in either direction. On 50% of trials the target stimulus contained a contrast increment at the pre-cued orientation. There was no contrast increment in the other trials. The task was a 2AFC in which participants indicated by button press whether the presented experimental stimulus contained an increment of contrast at the given orientation, or as it was phrased to them, whether ‘the indicated orientation was predominant in the image’. Per stimulus condition, two sessions were collected, leading to a total of 320 trials per orientation condition. All participants thus completed at least 6 sessions of the contrast detection task. Those participants (N = 10) who also performed the task on intact and scrambled natural scene conditions completed 10 sessions in total.

Participants started each session by training for four blocks of eight practice trials, one for each orientation condition. Stimuli were identical to the ones used in the main experiment, except that the orientation increment was set to 175% of the original orientation amplitude, in order to make the increments clearly visible to participants. Participants received feedback on a trial-by-trial basis as well as overall feedback at the end of the practice run. If their performance level was below 75%, task comprehension was checked, after which participants would complete the practice run once more.

#### Face identification task

All participants returned to the lab for a final session, in which they performed an identification task on upright and inverted faces. The stimuli were filtered to only contain information in a particular orientation band centered on vertical, 45° oblique, horizontal, or 135° oblique. Each trial started with a 500ms fixation period followed by the appearance of a face, which lasted for 300ms. After a random noise mask was presented for 200ms, a second face stimulus appeared for 300ms (Fig 3B). The location of the second stimulus was jittered randomly with a maximal displacement of 100 pixels in x and y direction to avoid matching on local low-level visual properties. Participants indicated by button press whether they perceived the first and second face to be identical or not. On 50% of trials, the two faces were identical and in 50% they were not. As in [5] the filter orientation and stimulus category was identical for both stimuli presented during a trial (e.g. a horizontally-filtered upright face stimulus is always followed by another horizontally-filtered upright face stimulus with either matching or non-matching face identity; Fig 3B). Filter orientation was randomized within blocks, whereas stimulus category (upright vs inverted faces) was blocked and block order was counterbalanced across participants. Thirty-two trials were collected per condition resulting in 256 trials in total.

Before the start of the experimental trials, participants trained for two blocks of 20 trials each (five trials per orientation condition), once with upright face stimuli and once with inverted face stimuli. During practice, participants were provided with trial-by-trial feedback.

### Analyses

Our aim was to investigate whether and how the profile of orientation selectivity is modulated by task and stimulus category. Therefore, we analyzed the relative performance differences across the four tested orientations to estimate the orientation selectivity profile of each individual participant and in both tasks. As the contrast detection task was set-up as a classical signal detection task, we computed d’. Since the identification task was not strictly speaking designed as a signal detection task (there was a response time limit and therefore ‘no response’ trials were possible), we relied on the percentage of accurate responses as a performance measure [43]. It turns out that there were not many “time-out” responses, and so in hindsight d’ might have been an appropriate measure as well. However, we decided to be most conservative about our measures, and to stick with accuracies in this case.

**Fig 4:**
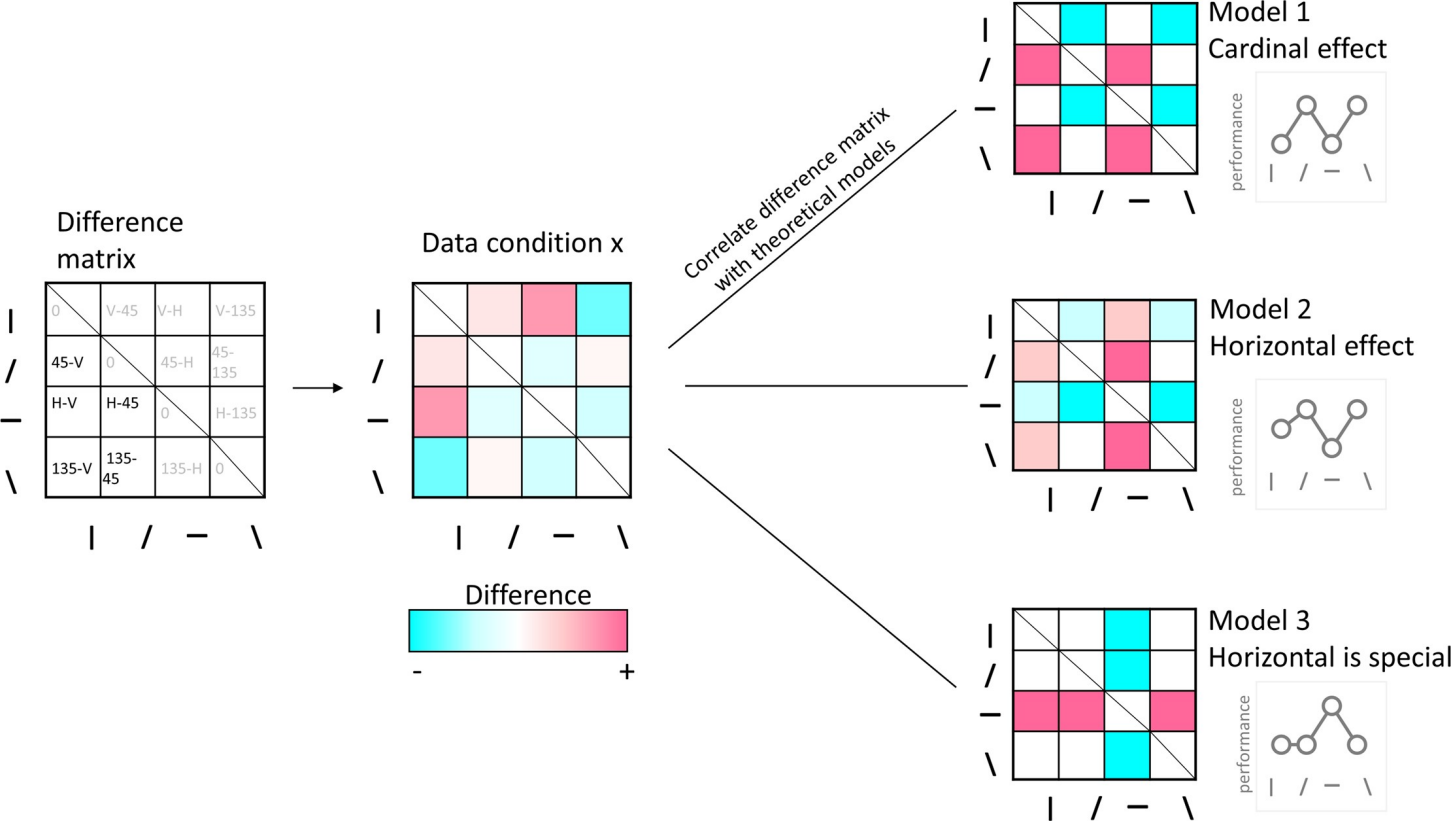
Performance difference matrix calculation and analyses. The left matrix indicates how cell values are computed. Equations in black represent values in cells that are included in subsequent correlation analyses. In these equations, the difference of performance between orientations is computed. Equations in grey represent values that were not included in subsequent analyses because they either replicate values in one the other half of the matrix or because they by definition will have a value of zero (diagonal). Right panel. Individual difference matrix of a given participant in a particular condition was either correlated with their matrix for another condition, or with matrices constructed based on *a priori* models of orientation selectivity. For each *a priori* model, the line plot insets illustrate the predicted performance as a function of orientation.

We subtracted the performance measure (i.e. d’ for the contrast detection task, and accuracy score for the face identity task) within one orientation from the performance measure acquired within another orientation (Fig 4). All binary comparisons of orientations were represented twice in the matrix: (a-b) and (b-a). We selected one of these comparisons and excluded the diagonal, resulting in individual vectors of six values. These vectors captured the orientation selectivity profile in each individual, each stimulus category and each task.

We then calculated the correlation between performance difference vectors in different conditions or between data and model vectors. All the correlation analyses presented here relied on the use of non-parametric Spearman rank correlation coefficients.

We tested three *a priori* models of orientation selectivity (Fig 4):

1. Model 1 (‘cardinal effect’): in line with the cardinal effect previously reported at primary stages of visual processing, Model 1 predicts that performance is similar for cardinal orientations, similar for oblique orientations, but lower for cardinal than oblique orientations. Any negative correlation with this model could be interpreted as reflecting the presence of an oblique effect (i.e., lower performance for oblique compared to cardinal orientations).
2. Model 2 (‘horizontal effect’): identical to Model 1, except that here we introduced a small performance decrement for the horizontal orientation to model the horizontal effect.
3. Model 3 (‘horizontal is special’): performance is superior in the horizontal range but similar across other orientations. This model represents the typical horizontal tuning for high-level face identification tasks.

This resulted in a single correlation value per participant for every matrix correlation of interest. These matrix correlations were then Fisher Z-transformed and tested against zero. Because transforming perfect correlations leads to infinite values, we set any Spearman rank correlations with a value of 1 to 0.99 before performing the Fisher transformation. We submitted the Fisher z transformed model correlations to a repeated-measure ANOVA with the factors of Task (2 levels: Contrast Detection, Face Identification), Stimulus category (2 levels: Upright Faces, Inverted Faces), and Model (3 Levels: Model 1, Model 2, Model 3). For all ANOVAs, we corrected the degrees of freedom using a Greenhouse-Geisser correction when the assumption of sphericity was not met (tested using a Mauchly test). Using the JASP statistical software (JASP Team (2018), JASP, Version 0.9) we additionally ran Bayesian analyses. We report the Bayes factors, which represent the relative consistency of the data compared to the data predicted by the statistical models under consideration [44]. In a Bayesian repeated measures ANOVA we investigated the evidence in favor of the absence (H0) or presence (H1) of any effects of Task (2 levels: Contrast Detection, Face Identification), Stimulus (2 levels: Upright Faces, Inverted Faces), Model (3 Levels: Model 1, Model 2, Model 3) and all their respective interactions. Dependent on the outcome of this analysis, we conducted appropriate follow-up analyses.

Some of the above models are correlated and therefore will partly explain the same variance within the data. In order to address whether the amount of variance explained by a given *a priori* model differed from the other two, we compared the absolute correlation coefficients across models in each condition.

The variability between the individual data matrices in the sample was used to estimate the maximally achievable data-model correlation. We computed the upper boundary of these noise ceilings by calculating the mean correlation of each individual’s data vector as acquired from their performance difference matrix (see above) with the average data vector across all participants. For the lower boundary, performance difference vectors as acquired from individual difference vectors were correlated with the average difference matrix of all other participants, but excluding the current individual ([44],[45], see Table 3).

Next, we tested the relationship between the orientation selectivity profiles across stimulus categories by correlating the vectors for upright and inverted faces for each individual participant. These analyses were conducted for the contrast detection task and face identification task separately. The correlation values were Fisher-Z transformed, and tested against zero and compared across tasks.

Finally, we compared the orientation selectivity profiles across tasks. Hereto, we computed the correlations of orientation selectivity vectors between the contrast detection and face identification tasks. After a Fisher Z-transform, both were tested against zero. These analyses were conducted for upright and inverted face conditions separately. The individual correlations between the contrast detection task and face identification task were compared across stimulus conditions.

Our approach shares commonalities with the Representational Similarity Analysis (RSA) framework in that we summarize the empirical data and *a priori* models in a similar format (i.e., using performance difference vectors) to analyze the similarity between the patterns observed in the data and the ones predicted by the *a priori* models [46–49]. A fundamental distinction however is that our correlation analyses are performed on differences in performance, whereas RSA is typically applied to measures of similarity (e.g., perceptual similarity ratings or correlations across brain response patterns). Although our approach resembles RSA to some extent, it is important to note that we do not assume that the performance difference measures analyzed here reflect the similarity of the underlying representations, as would be the case in the RSA framework. Performance could indeed be similar between two orientation conditions even in the case of a substantial difference in their internal representations.

For each comparison performed, we first verified the normality of the data (i.e., correlation coefficients or their difference) in each condition using diagnostic QQ plots. The assumption was met in all conditions. We therefore compared the coefficients against zero using parametric one-sample t tests and paired t tests to compare coefficients across models or conditions. All conducted tests were two-tailed and carried on Fisher-Z transformed coefficients. Critical alpha values for all post-hoc comparisons were Holm-Bonferroni corrected, which in the case of three comparisons means that critical alphas are .017, .025, and .05 for tests generating lowest to highest p-values, respectively.

Using Bayesian analysis, we tested the presence of correlation (H1) against the absence of correlation (H0), with correlation either referring to the correlation between our participants’ data matrices and the postulated models, or to the correlation between individual difference vectors in different conditions. We also ran Bayesian paired-samples t-test to directly compare between model correlations. For these comparisons, the prior on the effect size (i.e. Cohen’s d) was a normal distribution with a mean of 0 and standard deviation of 2. We opted for this prior distribution as centering it on zero implies that an effect in either direction is equally plausible. Furthermore, a prior SD of 2 implies about 68% probability of effect sizes varying between -2 and 2, and about 95% probability of effect sizes varying between -4 and 4. Most prior mass is thus allocated to commonly observed effect sizes, while we allow for more extreme effect sizes to be observed as well.

**Fig 5:**
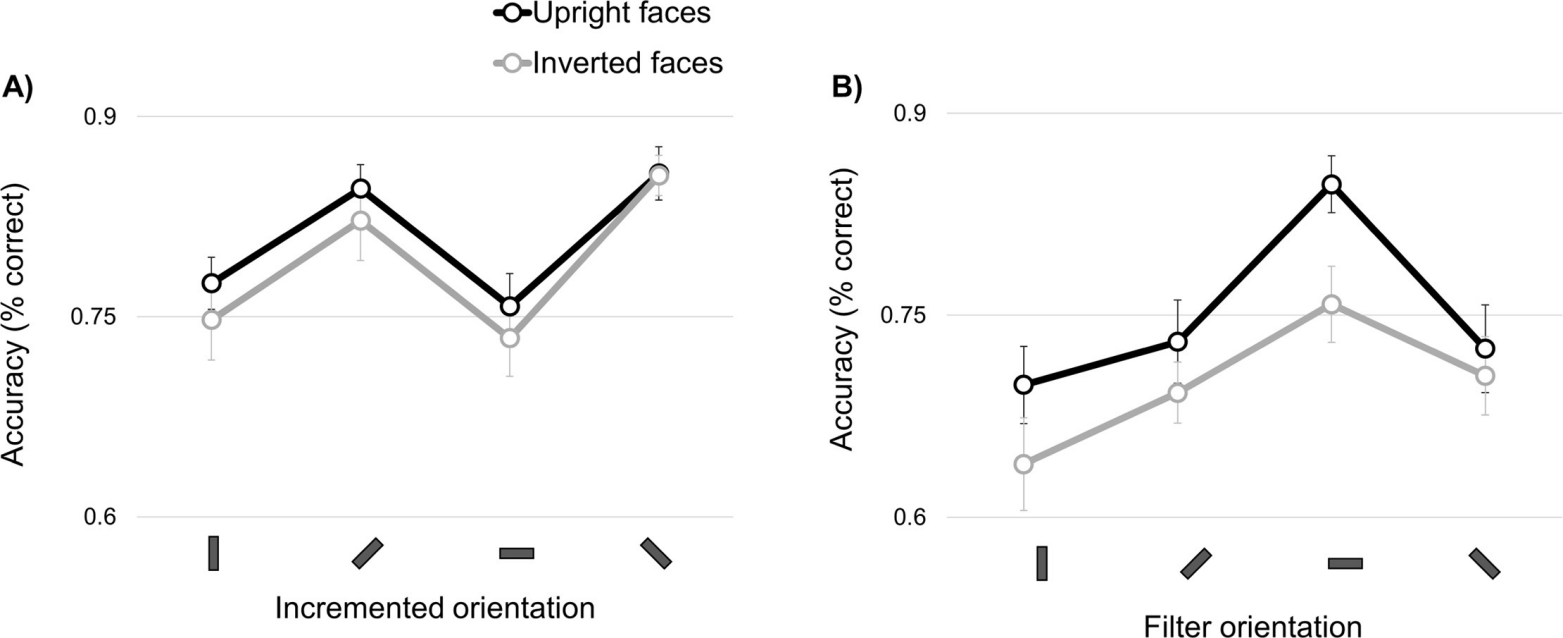
Results. A) Accuracy scores for the contrast detection task with the face conditions (i.e. upright faces, inverted faces). B) Accuracy scores of face identification task across filter orientations. The black lines represents the data for upright face stimuli, the gray lines represents the data for inverted face stimuli. Error bars indicate within-subject-corrected 95% confidence intervals [50,54].

Fig 5 shows the accuracy data from both experiments. For confidence intervals to be informative about the difference between conditions when observations are dependent (within-subject design), we applied the correction procedure as described in [50]. In the contrast detection task, the orientation selectivity profiles show a zig-zag pattern with higher sensitivity for the oblique as compared to the cardinal orientations. In contrast, sensitivity to identity is tuned to the horizontal orientation; this horizontal tuning is more pronounced in the upright as compared to inverted face conditions. In order to eliminate the influence of any baseline performance difference between individuals, we first normalized the data to the participant mean [51–53], before subjecting them to a repeated–measures analyses-of-variance testing for differences in the mean performance level across orientations. Since our main interest is the *relative* orientation selectivity *pattern*, rather than in the *absolute* performance difference between conditions, the results of these analyses are presented as Supporting Information. In these analyses, results are also compared to the scene data as acquired in a subset of 10 participants (see Methods).

## Results

First, we determined which of the *a priori* models of orientation selectivity best accounted for the sensitivity to contrast and identity in face images. To this aim, we compared the observed orientation selectivity profiles from the contrast detection and identification tasks to each of the three *a priori* models (see *Analyses*) and determined which of the models accounted for a significant portion of variance in the data.

The model correlations of the individual orientation selectivity profiles in each task and stimulus categories were then submitted to a repeated-measure ANOVA with Task, Stimulus and Model as within-subject factors. This analyses revealed a significant main effect of Task (F(1,23) = 10.99, p = .003). Also, the interaction between Task and Model was significant (F(1.26, 28.96) = 74.36, p< .0001) indicating that orientation selectivity profiles differ depending on the task carried out on the orientation content of face images. Our Bayesian ANOVA corroborates this finding as it assigns the greatest likelihood to the statistical model comprised of the main effects of Task and Model including their interaction (Task + Model + Task*Model) (P(M/data) = .814). There is little evidence that adding Stimulus has any effect on the model.

Separate ANOVAs were conducted for each task to explore this interaction further and showed that the main effect of Model was significant in both (contrast detection: F(1.3, 29.370) = 28.6, p< .0001; face identification: F(1.14, 23.48) = 44.66, p < .001). All other effects were not significant (Fs< 3.5, ps> .075). The non-significant effect of stimulus category suggests that upright and inverted faces involved similar orientation selectivity profiles. Bayesian analysis confirmed that statistical models consisting of a main effect of Model had the highest posterior likelihood, both in the contrast detection task (P(M/data) = .83) and in the face identification task (P(M/data) = .722).

### Observed versus *a priori* models of orientation selectivity–sensitivity to contrast

**Fig 6:**
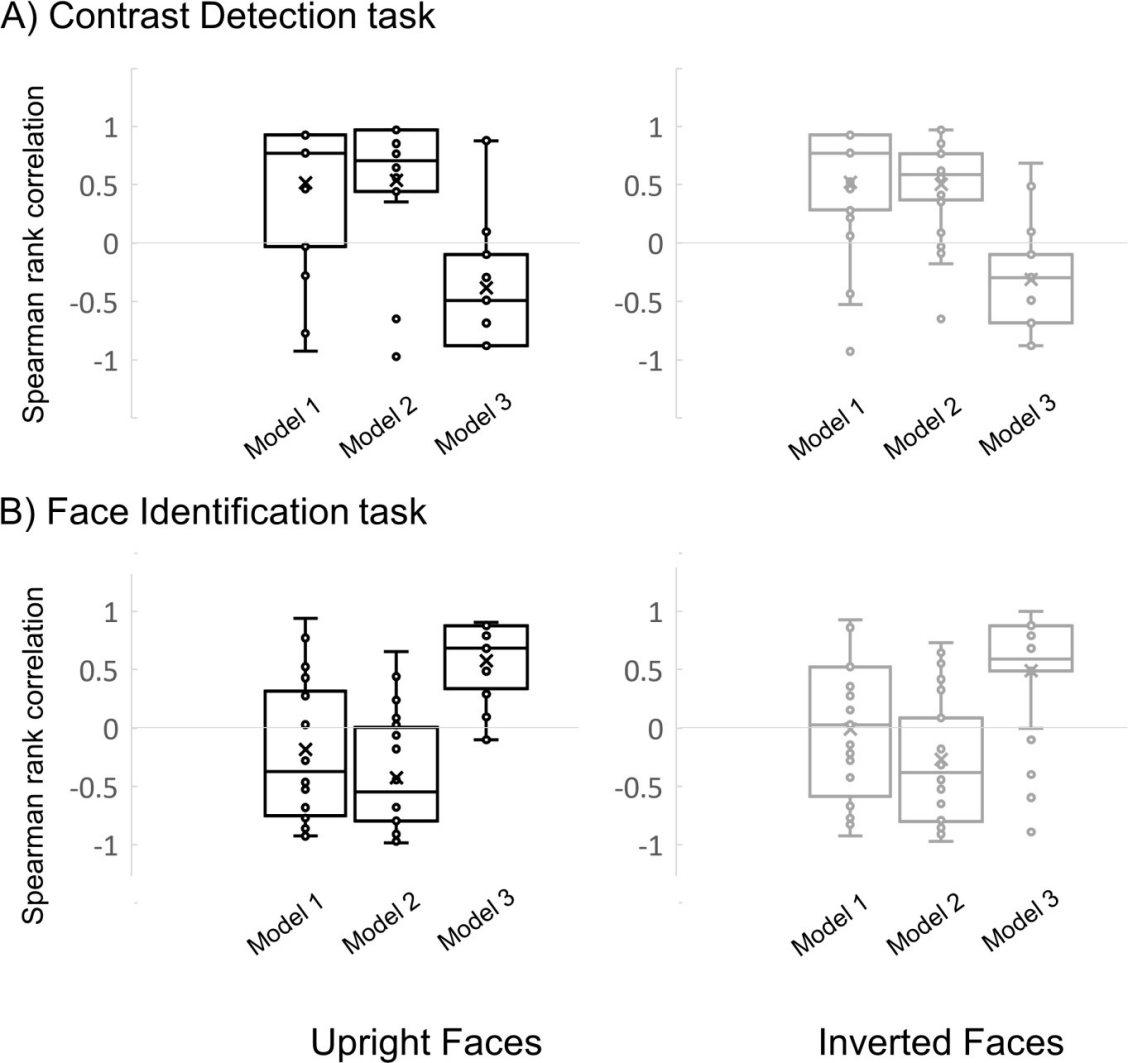
Comparing individual difference vectors to *a priori model*s. Box plots of the Spearman rank correlations between individual difference vectors and *a priori model*s split for different levels of task and stimulus category. The left column shows the model correlations for upright faces, whereas the right column shows the model correlations for inverted faces. The upper row shows the model correlations for the contrast detection task, whereas the lower row shows the model correlations for the face identification task. Model 1 represents the cardinal effect, Model 2 the horizontal effect, and Model 3 the idea that horizontal orientations lead to better performance from the other orientations (‘horizontal is special’). Horizontal lines within the boxes represents the median values, Xs represent the condition mean, and circles are the individual data points. Higher and lower edges of the boxes represent the borders of the third and first quartile, respectively.

The orientation selectivity pattern when the participants detected contrast increment in upright faces revealed positive correlations with Models 1 and 2 (Model 1: average r = .52; t_(23)_ = 4.38; P < .001, Model 2: average r = .54; t_(23)_ = 4.11; P < .001) and a significantly negative correlation with Model 3 (averaged r = -.38; t_(23)_ = -3.21; P = .004; Fig 6, upper left panel). Our Bayesian analyses show that in the upright face condition, the evidence in favor of Model 1 (‘cardinal effect’) over the null model is strong (BF_10_ = 131.1). There is also evidence in the data in favor of Model 2 (‘horizontal effect’, BF_10_ = 68.2) and Model 3 (‘horizontal-is-special’, BF_10_ = 8.2) (see Table 1). Inspection of the original data (Fig 5A) showed that overall performance was highest for the two oblique orientations, followed by vertical, and it was lowest for the horizontal orientation. The positive correlation with Models 1 and 2 reflects this cardinal/horizontal effect, and the additional negative correlation with Model 3 results from worse detection of contrast in the horizontal range. Sensitivity to contrast in upright faces was found to be better accounted for by Model 2 compared to Model 3 (t_(23)_ = 3.025, p = .006, BF_10_ = 5.38; other comparisons: ps>.11, BF_10_ < 0.38).

**Table 1:** Bayesian factors for the *a priori* model correlations. BF10 values represent the strength of the evidence in favor of a positive model correlation over the alternative of no correlation.

		BF_10_
**Contrast detection**		
Upright faces	M1	131.1
	M2	68.2
	M3	8.16
Inverted faces	M1	190.8
	M2	558.5
	M3	14.7
**Face identification**		
Upright faces	M1	.031
	M2	55.3
	M3	91912
Inverted faces	M1	.10
	M2	2.03
	M3	103.3

The contrast detection data for inverted face stimuli correlated positively with Model 1 (averaged r = .52; t_(23)_ = 4.53; P < .001) and Model 2 (averaged r = .5; t_(23)_ = 4.97; P < .001), and negatively with Model 3 (averaged r = -.31; t_(23)_ = -3.46; P = .002; Fig 6, upper right panel). Models 1 and 2 were both found to result in larger and comparable absolute correlation coefficients than Model 3 (t_(23)_ = 2.82, p = .01, BF_10_ = 3.4 and t_(23)_ = 2.66, p = .014, BF_10_ = 2.4, respectively). The strongest evidence favors Model 2 (BF_10_ = 558.5), followed by Model 1 (BF_10_ = 190.8) and last Model 3 (BF_10_ = 14.7; see Table 1).

In summary, the orientation selectivity profile of the sensitivity to primary contrast in face images was accounted by all three *a priori* models for both upright and inverted face stimulus conditions. Bayesian analysis indicated that evidence in favor of both horizontal effect model and cardinal effect model were stronger compared to the horizontal is special model. In the upright face condition, evidence was strongest for the cardinal effect model, whereas in the inverted face condition, it was strongest for the horizontal effect model. Sensitivity in this task additionally correlated negatively with the ‘horizontal is special’ model, in line with the selectively worse sensitivity to contrast in the horizontal orientation range.

### Observed versus *a priori* models of orientation selectivity–sensitivity to identity

The orientation selectivity profile for upright face identification demonstrated a positive correlation with Model 3 (averaged r = .58; t_(23)_ = 7.13; P< .001) and a negative correlation with Model 2 (averaged r = -.42; t_(23)_ = -4.02; P < .001; Fig 6, lower left panel), as also evidenced by the large corresponding Bayes Factors (Model 2: Bf_10_ = 55.3, Model 3: BF_10_ = 9.19 *10^4^).

The positive correlation with the ‘horizontal is special’-model for upright faces is caused by the better identification performance in the horizontal band (Fig 5B). The negative correlation with Model 2 reflects that performance tended to improve in the horizontal range whereas Model 2 predicts a lower performance.

Akin to upright faces, inverted face sensitivity correlated positively with Model 3 (averaged r = .49, t_(23)_ = 4.28, p < .001, BF_10_ = 103.3), and negatively with Model 2 (averaged r = -.26, t_(23)_ = -2.57, p = .017, BF_10_ = 2), although Bayesian analyses do not provide strong support for this latter relationship.

Bayesian analyses supports the absence of correlation between Model 1 and task performance for both upright and inverted faces (BF_01_ values > 3.3).

Direct comparison of the correlation coefficients did not reveal any difference in the extent to which the models explain the observed sensitivity to upright and inverted face identity (all Ps > .043, BF_10_ < 17.8). Furthermore, the coefficients of *a priori* model correlations were comparable for upright and inverted face identification (two-tailed t tests for paired samples: ps> .32).

In sum, the identification of both upright and inverted faces was accounted by the two *a priori* models, which depict a distinct processing regime for horizontal orientations. The positive correlation with the ‘horizontal is special effect’ model corroborates past evidence that horizontal cues are most diagnostic when processing face identity. Unsurprisingly, the correlation with the model predicting worse performance in the horizontal range (‘horizontal effect’) is negative. Bayesian analyses indicate that face identification evidence was more in favor of the ‘horizontal is special ‘.

### Orientation selectivity profiles across stimulus categories and tasks

**Fig 7:**
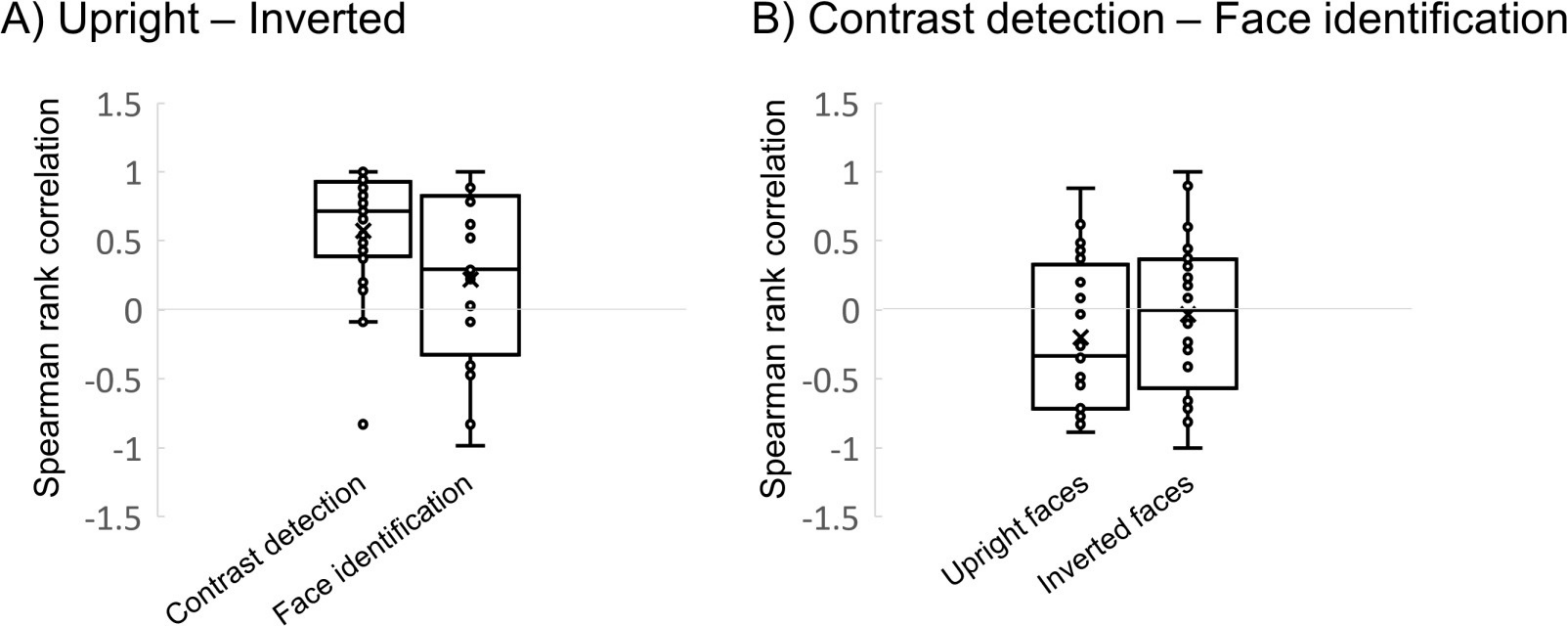
Comparing individual difference vectors across experimental conditions. A) Spearman rank correlation of individuals’ difference vectors for upright and inverted faces. The left bar depicts the correlation for the contrast detection task. The right bar depicts the correlation for the high-level identification task. B) Spearman rank correlation of individuals’ difference vectors for contrast detection and face identification tasks. The left bar depicts the correlation for the upright face condition. The right bar depicts the correlation for the inverted face condition. Black horizontal lines within the boxes represents the median values, Xs represents the condition mean. Higher and lower edges of the boxes represent the borders of the third and first quartile, respectively.

The above analyses did not reveal any differences in the way performance with upright and inverted faces loaded on *a priori* models of orientation selectivity. To further investigate the functional relation between upright and inverted orientation selectivity profiles, we correlated the individual difference vectors of both conditions in each task (see Fig 7). Significant positive correlation appeared between the individual difference vectors of upright and inverted faces for the contrast detection performance (averaged r = .57; t_(23)_ = 5.1; P < .0001). The evidence in favor of a significant correlation between upright and inverted contrast detection orientation selectivity profiles is very strong (BF_10_ = 835.6, see Table 2).

**Table 2:** Bayesian factors for the difference matrix correlations across tasks and across stimulus categories. BF10 values represent the strength of the evidence in favor of a correlation between the tested patterns of orientation selectivity over the alternative of no correlation.

	BF_10_
**Stimulus correlations**	
Contrast detection	835.6
Face identification	.29
**Task correlations**	
Upright faces	.0.45
Inverted faces	.0.10

No such correlation existed for the face identification performance (averaged r = .22; t_(23)_ = 1.46; P = .16; BF_10_ = .29; Fig 7, left panel). In fact, the Bayesian analyses showed that the null hypothesis of no correlation between upright and inverted face data was three times more likely (BF_01_ = 3.44). Direct comparison of the upright-inverted correlation across the two tasks revealed no significant difference (t_(23)_ = 1.9, p = .069, BF_10_ = .58).

These results indicate that at the individual level, similar orientation preferences operate on upright and inverted faces when the task is to detect contrast increments in the face stimulus. However, when asked to match identity, we note a relative dissociation of the orientation selectivity profiles for upright and inverted faces.

As a final step, the data from both tasks were directly compared. For neither upright (averaged r = -.2, t_(23)_ = -1.76, P = .09) nor inverted faces (averaged r = -.033, t_(23)_ = -.18, P = .86; Fig 7, right panel) was the correlation between orientation selectivity profiles for contrast and identity higher than zero. The difference in task correlation between upright and inverted proved non-significant (t_(23)_ = -0.83, p = .41). The complementary Bayesian analyses provided evidence in favor of the absence of correlation, mostly for the inverted face conditions (BF_01_ = 9.69) and to some extent for the upright face conditions (BF_01_ = 2.21) (see Table 3). This data further demonstrates the dissimilarities in orientation selectivity at primary and high-level stages of processing, even when operating on the same stimulus category.

**Table 3:** Noise ceilings for the *a priori* model correlations. Noise ceilings indicate the maximal correlation achievable by a given model with the experimental data, given the data variability across participants (see Analyses section for details on calculation).

	Noise Ceiling	
	Min	Max
**Contrast Detection**		
Upright faces	0.54	0.57
Inverted faces	0.56	0.6
**Face Identification**		
Upright faces	0.54	0.58
Inverted faces	0.51	0.54

## Discussion

The experience of our visual environment primarily grounds on the orientation-selective encoding of contrast. Little is known on how primary orientation-selective mechanisms operate upon naturalistic and complex input such as the faces of our peers, and on how they drive higher-level computational stages. Our work narrows this gap by studying the orientation-selective encoding of contrast in face images and investigating its link to the orientation biases for face identification, a presumably high-level visual process. In each condition and for each individual, we derived a profile of orientation selectivity, i.e. a relative measure that is suitable for the comparison across tasks and stimuli.

Our analyses showed that when instructed to detect an increment of contrast at a specific orientation, humans were better with oblique as compared to cardinal orientations, with an additional horizontal suppression. This cardinal\horizontal suppression appeared unspecific to the face category (i.e. upright vs inverted). These results resemble the cardinal\horizontal suppression observed previously with other categories of broadband stimuli (20,23; see S1 File of Fig 1A). It has been suggested that primary tuning away from horizontal orientations serves to counteract natural anisotropies in the visual environment (i.e. whitening), an explanation which could apply to faces as well, as they are also dominated by horizontal information [4,6,8,35]. Hansen and Essock [28] further showed that the horizontal effect is especially strong for horizontally sparse images. Face images are horizontally sparse: most of their energy is concentrated at the level of the horizontally-structured brows, eyes, and mouth cues [4,35,55]. Whitening is therefore a plausible explanation for the horizontal effect occurring for face images.

In order to address the functional link between primary and higher-level orientation biases more directly, we first compared the primary orientation selectivity profile for upright and inverted face stimuli. When presented in a canonical upright plane orientation, human faces trigger robust and selective responses in certain areas of high-level visual cortex. Inversion in the picture plane drastically reduces the high-level responses to faces. In contrast, inversion leaves the overall V1 response unchanged [13], probably because upright and inverted faces are comparable in terms of their global orientation and spatial frequency content. The individual orientation selectivity profiles of contrast detection in upright versus inverted face stimuli correlated positively. From this, we conclude that at primary visual stages, the processing of oriented contrast share commonalities among upright and inverted faces. In line with these results, we previously found lower activation in primary visual cortex (V1) in response to horizontally-filtered face images than to vertical or oblique orientations [13]. Again, this effect was not specific to upright faces, but was observed for inverted and scrambled faces. These results suggest that the primary tuning away from cardinal\horizontal orientations originates from V1 with little impact of higher-order image structure. It might reflect the general normalization mechanism that operates on broadband images and that has developed to adaptively deal with anisotropies in natural image statistics.

Our approach may have been insensitive to subtler differences in orientation selectivity between upright and inverted faces, in line with the marginally different orientation decoding patterns observed in V1 BOLD response for upright versus inverted faces [13]. These subtle differences in V1 orientation selectivity for upright and inverted faces might reflect modulatory influences by higher-level processes. Alternatively, they may reflect differences in orientation selectivity across the visual field. Indeed, while the upright face is a top-heavy stimulus, i.e. it contains most of its contrast in the upper part [56–58], the inverted face is bottom-heavy. Because early visual regions are retinotopic, they activate different populations of neurons for upright and inverted faces, populations that may differ in their orientation selectivity profile. Nevertheless, since behavioral data reflects neural processing at the system level, it is not suited to address such fine-scale differences in V1 orientation selectivity profiles for upright and inverted faces. Moreover, the observation that the orientation biases of the contrast detection task for upright and inverted faces were positively correlated supports the notion that the processing of oriented contrast share some commonalities among upright and inverted faces.

The orientation selectivity profile for face identification differed from the cardinal\horizontal suppression observed for contrast detection. The identification of both upright and inverted faces correlates positively with the ‘horizontal is special’-model we predefined based on these previous studies. As a matter of fact, our past evidence showed that inversion varies the peak amplitude and bandwidth of the psychometric curve relating identification performance to orientation, but preserves the horizontal peak; in other words, inversion is not expected to qualitatively change the overall orientation selectivity profile of face identification and this is what we observe here. Yet, the most probably null correlation between upright and inverted orientation selectivity profiles in the face identification task suggests a relative dissociation of orientation biases when processing upright versus inverted face identity. Accordingly, we have previously demonstrated an upright face-specific horizontal tuning in Fusiform Face Area (FFA). Furthermore, we showed that brain activity patterns in FFA allow classification of upright and inverted faces only when the presented images are horizontally-filtered.

Although we carefully designed our experiment to prevent pixel-based matching of the images, participants might have adopted unanticipated strategies when executing the face identification task, for example by matching faces based on the large-scale contrast distribution across the image. If this were the case however, it should have led to a greater similarity in orientation selectivity profiles, and thus increased the likelihood of finding a significant correlation across stimulus categories and tasks.

We further investigated the link between primary and high-level orientation dependencies by addressing whether the orientation selectivity profile for the detection of oriented contrast is related to orientation preferences for the identification of faces. Correlation analyses indicated that there is most likely no correlation between the orientation selectivity at primary and higher levels of processing for neither of the two categories. The Bayes factor provides evidence favoring the absence of such a correlation, making this result a meaningful aspect of our data. These findings agree with a recent study by Duncan et al. (2019). The authors show that the horizontal tuning for the processing of face identity is positively linked to individual face recognition performance but cannot be explained by the general sensitivity to horizontal contrast as measured with Gabor stimuli [59].

The present findings are not in line with recent evidence that primary visual encoding depends on the higher-level image content. For example, access to a higher-level representation has been shown to boost or hamper the processing of basic attributes, depending on whether a stimulus is near or supra- threshold [60–63]. Two recent works showed that the orientation-selective processing of Gabor patches sharpens under the influence of the higher-level representation of the natural scene in which it is inserted [33,34]. While Teufel and colleagues [33] suggest that such modulations stem from the high-level semantic interpretation of the image, Neri [34] interprets his findings as arising from mid-level stimulus analyses, as they were not modulated by manipulations hampering stimulus semantic interpretation. However, we need to be cautious while comparing our findings to those previous works. Indeed several major aspects of their methodology may explain the empirical divergences and point to the factors potentially influencing the balance between bottom-up versus top-down influences in vision. First, the scene stimuli used in these previous studies were diverse, cluttered and unpredictable. In contrast, our face stimuli were highly homogeneous, sparse, and predictable. It is plausible that higher-level influences on primary visual computations are strongest with noisier, less predictable images therefore hampering the access to local shape contours at primary processing stages and relying on mid- or high-level representations to disambiguate figure from ground.

Another important aspect is that in both the Neri [34] and Teufel et al. [33] studies, participants were instructed to detect and extract the orientation of a local narrowband Gabor patch inserted either on the contour of a shape, or on an irrelevant contour. This stimulus-task combination involved processing of shape boundaries, likely encouraging higher-level influences. In the work by Teufel et al. [33] this was implicitly so, while in Neri [34] participants explicitly judged the alignment of a Gabor patch to a local contour. In contrast, the energy increments we applied to face images were broadband and diffuse and therefore less tied to the underlying shape.

Image analyses have shown that variability across face identities is largest for horizontal components around the eye and mouth region, and that the horizontal tuning links to a preferential reliance on the eyes when processing face emotion [4,8,35,64]. Accordingly, face identification may not entail a global tuning towards horizontal orientation, but rather a spatially specific horizontal tuning, taking into account the spatial order of facial features (see 51). Such a spatial integration of primary orientation information may explain why we did not find any face-specific tuning with our global contrast increments.

The current study shows the primary orientation selectivity profile for contrast detection in face stimuli. It can be characterized by the cardinal\horizontal effects that have been previously found with broadband natural scenes. Individual primary orientation profiles were not related to the horizontal tuning for face identification, which triggers new questions on how the horizontal tuning to face identity arises in the visual system. If horizontal tuning to identity is in fact independent of primary orientation processing biases, then this implies that at some stage in the processing course the orientation selectivity profile transforms from a general cardinal\horizontal suppression to a spatially specific horizontal preference for the processing of face identity. We aimed to provide potential answers as well as considerations for future studies into this topic. A topic, which we consider to be of great importance, especially within the broader context of how visual experience emerges from its primary building blocks.

## Supporting information

S1 File Analyses of Variance (ANOVA). (DOCX)

S2 File Mat-files containing relevant data. (ZIP)

S3 File Stimuli. (ZIP)
