# Supplementary material for: Contrast versus identity encoding in the face image follow distinct orientation selectivity profiles
Source: PLoS One. 2020 Mar 18;15(3):e0229185. doi: 10.1371/journal.pone.0229185 (PMC7080280; doi:10.1371/journal.pone.0229185)
Supplement: S1 File — (DOCX) [file pone.0229185.s001.docx]

# Supporting information: Analyses-of-Variance (ANOVAs)

# Analyses

For the contrast detection task d’ values were calculated as a measure of sensitivity for each condition. Data were standardized to the participant’s individual mean and these normalized values served as input for all further analyses. We decided to normalize the data so that further analyses were conducted on the variance around the individual mean. We were not interested in absolute differences between orientation conditions and therefore we eliminated the influence of any baseline performance difference between individuals through normalization. A repeated-measures ANOVA was conducted to test the effect of *Incremented Orientation* (4 levels: vertical, 45° oblique, horizontal, 135° oblique). The face-specificity of detected orientation effects was tested through the *Incremented Orientation* by *Stimulus Category* (3 levels: upright, inverted, and scrambled faces) interaction.

In the group of ten participants who also completed the contrast detection task with scene stimuli, the presence of a horizontal effect for scene stimuli, as previously reported in the literature (Essock et al., 2003; Hansen & Essock, 2004, see Introduction), was tested by conducting a one-way repeated-measures ANOVA with factor *Incremented Orientation.* Again, we checked for face-specificity of the results with upright faces by directly comparing the orientation selectivity profile across these two stimulus categories as an *Incremented Orientation* by *Stimulus Category* (2 levels: upright faces and scenes) interaction. If any differences between these two categories were observed, we proceeded to test the contrast detection performance for scrambled scenes and scrambled faces to investigate whether these differences can be explained by the difference in spectral slope across the two images. Natural scenes tend to have a 1/f spectral slope (i.e. energy distribution across spatial frequencies), whereas face images tend to have a 1/f^2^ spectral slope. In our stimulus set, scenes and faces were matched for orientation, and global luminance and contrast. The only remaining difference between the phase-scrambled versions of both stimulus types that can explain any possible difference in contrast detection performance is then the spectral slope.

On the standardized face identification accuracies, a two-way repeated-measures ANOVA with factors *Filter Orientation* (4 levels: vertical, 45° oblique, horizontal, 135° oblique) and *Stimulus Category* was conducted to test for the Face Inversion Effect, i.e. the drop in face identification performance from upright to inverted faces and the upright face-specific horizontal preference as reported in the literature previously (e.g. Goffaux & Greenwood, 2016, see Introduction).

For all ANOVAs, a Greenhouse-Geisser correction was applied to the degrees of freedom if equal variances could not be assumed. If there were any previous hypothesis on directionality of effects, one-tailed p-values were reported, which is explicitly reported in the Results section. Critical alpha values (.05) for all post-hoc comparisons were Holm-Bonferroni corrected.

# Results

## Sensitivity to contrast across orientations for upright, inverted, and scrambled faces

The one-way repeated measures ANOVA testing the performance for contrast detection in upright faces across the different orientations showed a significant effect (F_(3,69)_ = 18.94, p <.001). Sensitivity scores were significant for all post-hoc comparisons (all Ps< .022), except for the horizontal versus vertical comparison, where they only showed a trend (P = .007, Cohen’s D = .457). These results show that sensitivity indeed varies with the orientation of the amplified contrast, and that sensitivity is higher for oblique as compared to cardinal orientations.

The two-way repeated-measure ANOVA showed a main effect of *Stimulus Category* (F_(2, 46)_ = 10.63; P < .001; partial η^2^ = .316), a main effect of *Incremented Orientation* (F_(2.3, 53.0)_ = 27.9; P < .001; partial η^2^ = .548), but no interaction effect between these two factors (F_(6, 138)_ = 1.89; P = .11; partial η^2^ = .071; Suppl. Fig. 1A). Performance was significantly better for scrambled faces versus inverted faces (P < .001; Cohen’s D = .976). Post-hoc comparisons for the different levels of *Incremented Orientation* actually showed significant differences between all levels (mean absolute d’: vertical = 1.94, 45° = 2.27, horizontal = 1.69, and 135° = 2.46; all Ps < .032). These analyses thus indicate that the cardinal effect is not specific to upright, intact face stimuli, but also occurs when tested with inverted faces or with phase-scrambled faces.

## Comparison of contrast increment sensitivity between faces and natural scenes

In the subset of participants (N = 10) who performed the contrast detection task also with scene stimuli, there was a significant effect of *Incremented Orientation* (F_(3,27)_ = 12.7; P < .001; partial η^2^ = .59). This was driven by differences between horizontal increments and both oblique increments (P = .014; Cohen’s D = -1.238, P =.001; Cohen’s D = -1.889) and between vertical increments and both oblique increments (P = .039; Cohen’s D = -0.976, P =.001; Cohen’s D = -1.803). There was no difference in detection performance of horizontal and vertical orientations (P = .503). Thus, we found a cardinal effect for natural scene stimuli, but did not manage to replicate the horizontal effect as previously reported with natural scene images (Hansen & Essock, 2004). This could be due to the horizontal effect generally being quite small and our relative small sample size not delivering the necessary power to show such small effects.

Comparing upright faces and scenes directly in this participant group revealed a higher sensitivity to contrast increments of any orientation when tested with faces as compared to scenes (F_(1,9)_ = 10.5; P = .01; partial η^2^ = .54), but orientation selectivity did not differ between the two categories (F_(3,27)_ = .323; P = .81; Suppl. Fig 1B). Interestingly, the overall sensitivity to contrast increments tended to reverse for the scrambled versions of the face and scene stimuli with higher detection accuracies for scrambled scenes compared to scrambled faces (F_(1,9)_ = 3.64; P = .089; partial η^2^ = .29; Suppl. Fig. 1C). From this, we can conclude that differences in spectral slope between faces and scenes do not explain the differences in contrast sensitivity that we observe here when faces and scenes are intact.

*Identification sensitivity for orientation-filtered (upright and inverted) faces.*

The face identification data showed a face inversion effect meaning higher performance levels with upright as compared to inverted faces (F_(1, 23)_ = 26.0; P < .001; partial η^2^ = .531; Figure 5B). As expected, performance was best with horizontally filtered stimuli and indeed, all post-hoc contrasts with the other three orientation filter conditions were significant (all Ps < .001). However, inversion did not significantly modulate the pattern of performance across orientations (F_(3,69_) = 1.71; P = .17). Post-hoc comparisons revealed that the effect of face inversion was larger in the horizontal as compared to the oblique conditions (both Ps < .042). However, there was no difference between the horizontal and vertical conditions (P = .31). As the difference in face inversion effect for horizontal and vertical conditions was the crucial comparison in our previous work on orientation tuning to face identity ( Goffaux & Dakin, 2010; Goffaux, van Zon, & Schiltz, 2011), we concentrated our further analyses on these two orientation conditions. Identification performance was significantly higher for horizontally versus vertically filtered upright faces (t_(23)_ = 8.28, P<.001, Cohen’s D = 1.69). This was also the case for inverted faces, although the size of this effect was smaller (t_(23)_ = 4.91, P<.001, Cohen’s D = 1.0).

**Supplementary figures**

**
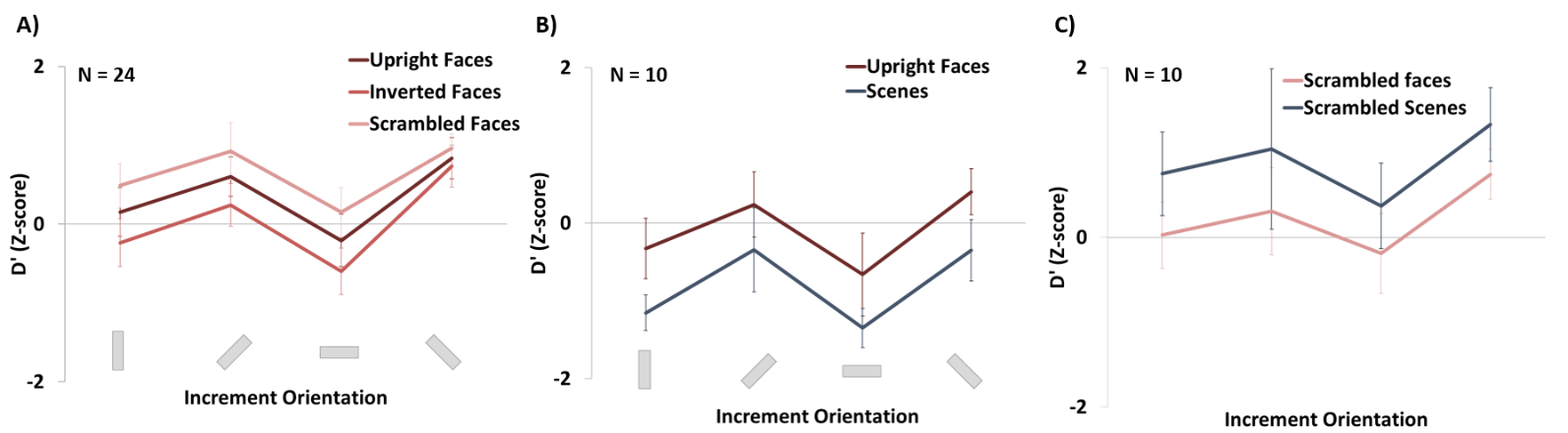
**

**S1 Fig 1. Group-averaged contrast detection performance.** A) Mean normalized d’ scores of all 24 participants for the three versions of the face stimuli: upright faces, inverted faces, and phase scrambled faces. B) Mean normalized d’ scores for upright faces and scenes in the subset of 10 participants who also performed the contrast detection task with natural scenes. C) Mean normalized d’ scores for scrambled faces and scrambled scenes in the subset of 10 participants that also performed the contrast detection task with natural scenes. Error bars indicate 95% confidence intervals expressing the variation in normalized D=prime scores across participants.

**
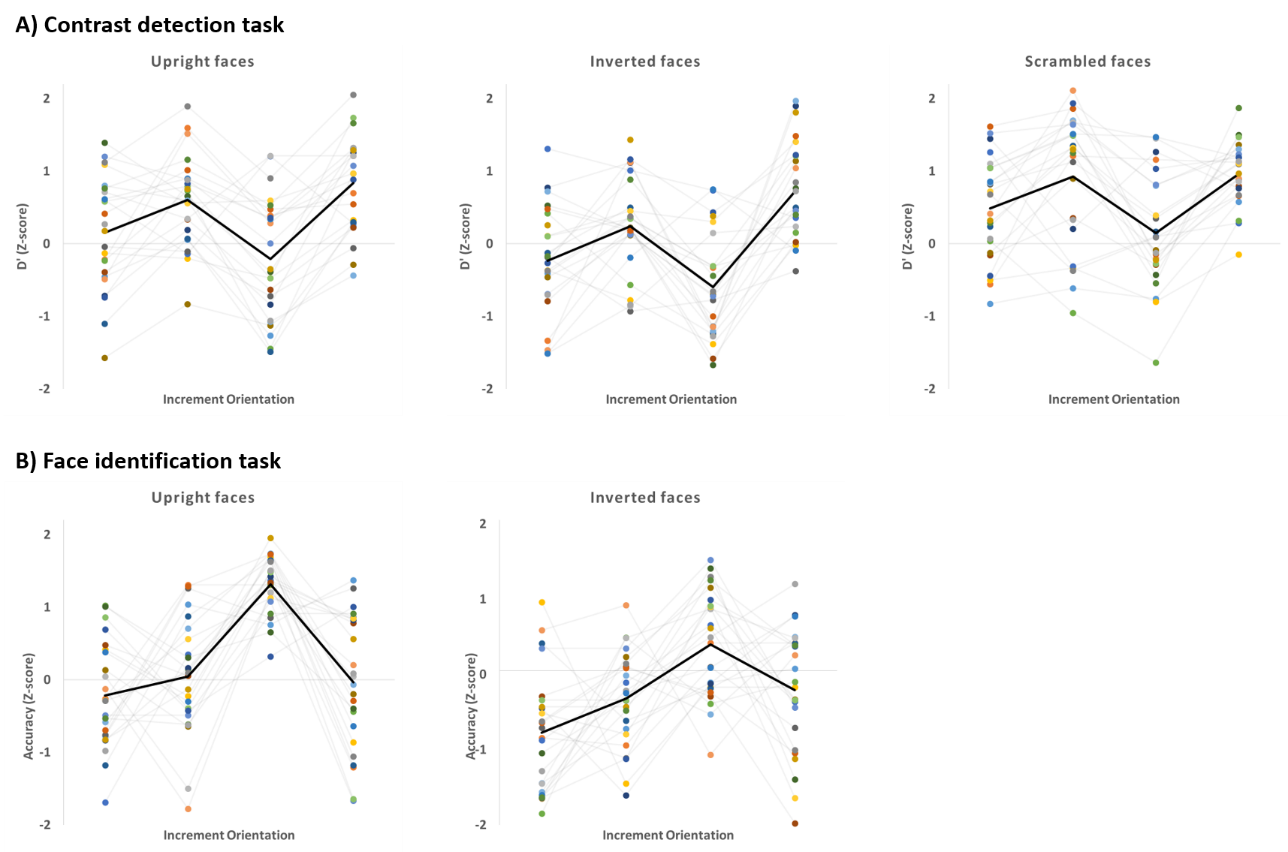
**

**S1 Fig 2. Individual performance in the contrast detection and face identification tasks.** A) Individual normalized d’ scores of all 24 participants for the three versions of the face stimuli: upright faces (left panel), inverted faces (middle panel), and phase scrambled faces (right panel) in the contrast detection task. B) Individual normalized d’ scores of all 24 participants for the upright faces (left panel) and inverted faces (right panel) in the face identification task. Light grey lines connect data points of individual participants, black lines connect the mean d’.
