# Supplementary figures and images for: Contrast versus identity encoding in the face image follow distinct orientation selectivity profiles (part 2 of 2)
Source: PLoS One. 2020 Mar 18;15(3):e0229185. doi: 10.1371/journal.pone.0229185 (PMC7080280; doi:10.1371/journal.pone.0229185)

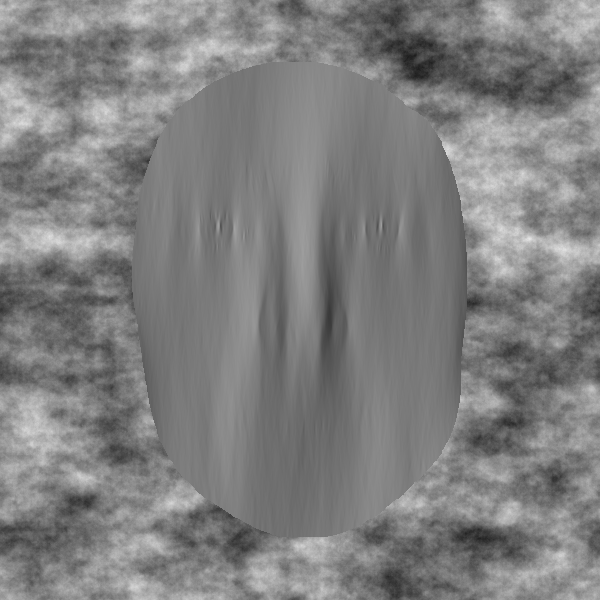

Supplement: S3 File — (ZIP) [file pone.0229185.s003.zip › eq_m14_a.bmp_0.bmp]

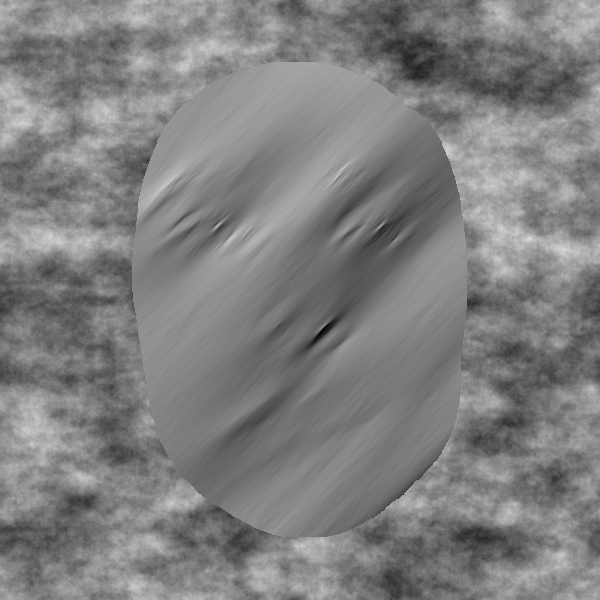

Supplement: S3 File — (ZIP) [file pone.0229185.s003.zip › eq_m14_a.bmp_135.bmp]

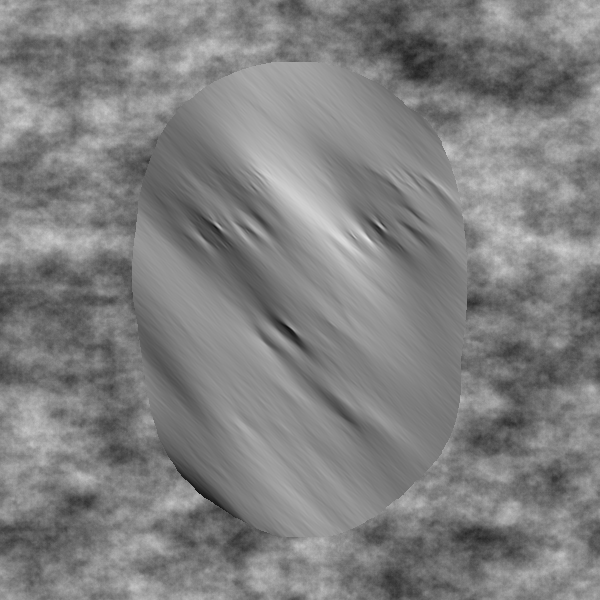

Supplement: S3 File — (ZIP) [file pone.0229185.s003.zip › eq_m14_a.bmp_45.bmp]

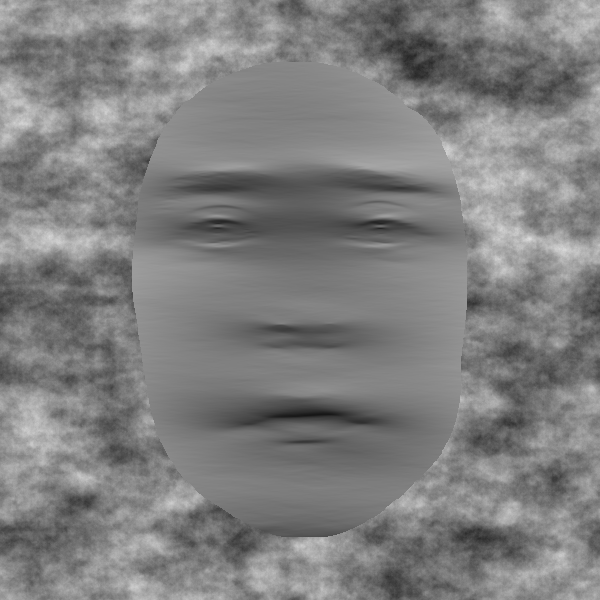

Supplement: S3 File — (ZIP) [file pone.0229185.s003.zip › eq_m14_a.bmp_90.bmp]

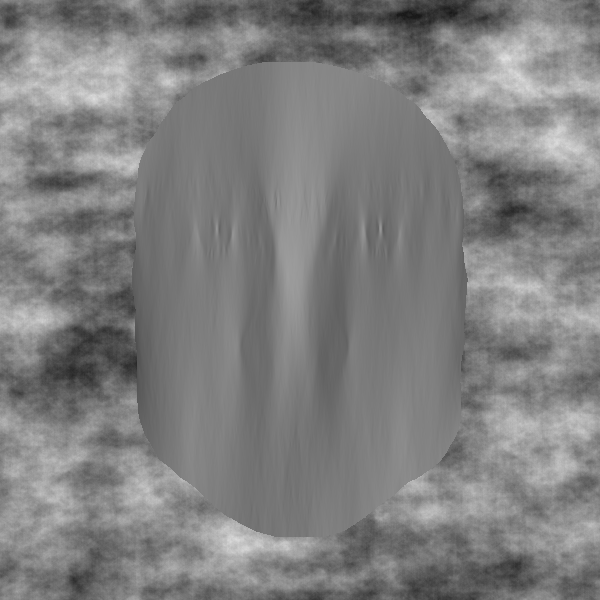

Supplement: S3 File — (ZIP) [file pone.0229185.s003.zip › eq_m16_a.bmp_0.bmp]

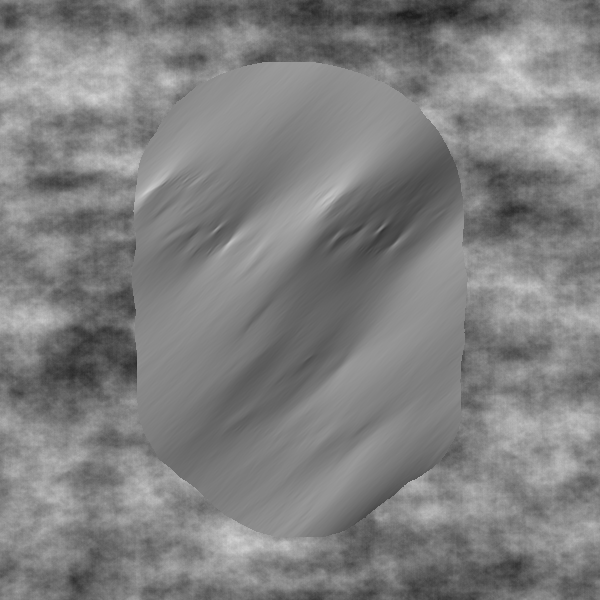

Supplement: S3 File — (ZIP) [file pone.0229185.s003.zip › eq_m16_a.bmp_135.bmp]

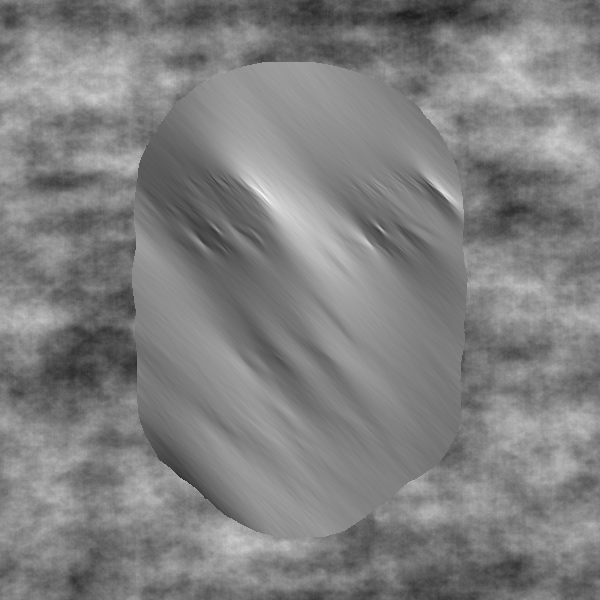

Supplement: S3 File — (ZIP) [file pone.0229185.s003.zip › eq_m16_a.bmp_45.bmp]

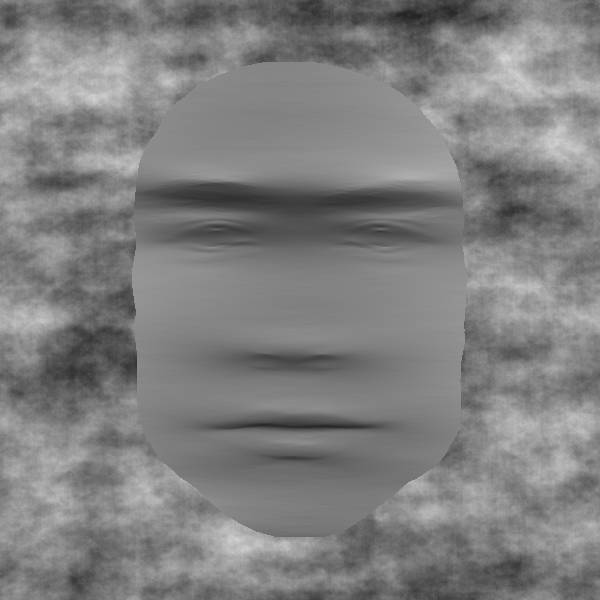

Supplement: S3 File — (ZIP) [file pone.0229185.s003.zip › eq_m16_a.bmp_90.bmp]

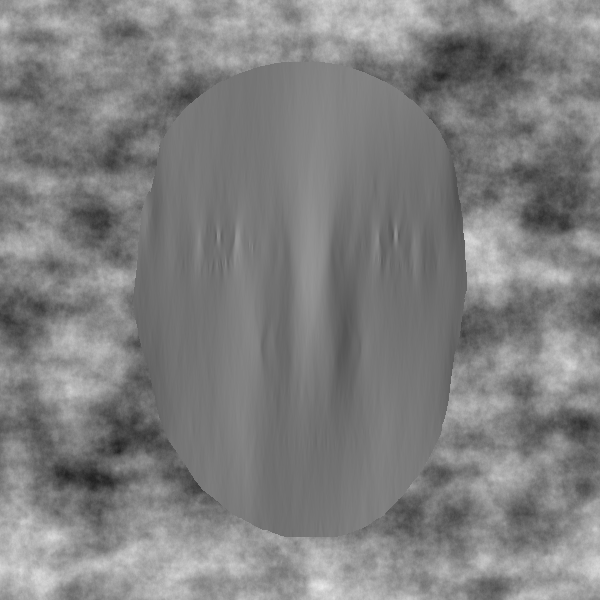

Supplement: S3 File — (ZIP) [file pone.0229185.s003.zip › eq_m17_a.bmp_0.bmp]

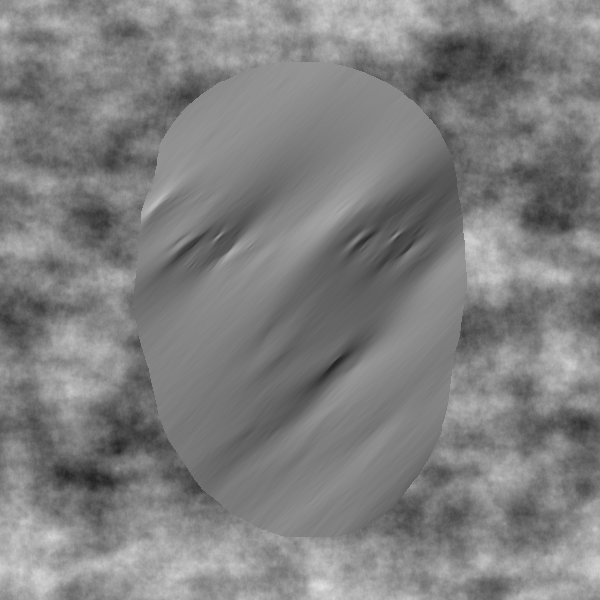

Supplement: S3 File — (ZIP) [file pone.0229185.s003.zip › eq_m17_a.bmp_135.bmp]

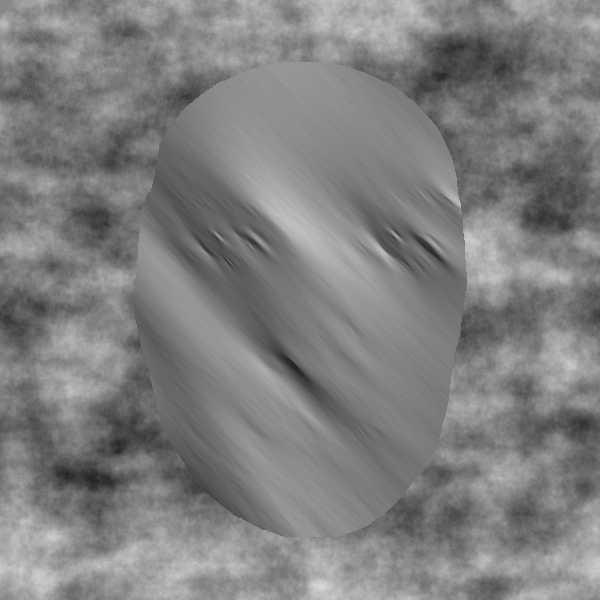

Supplement: S3 File — (ZIP) [file pone.0229185.s003.zip › eq_m17_a.bmp_45.bmp]

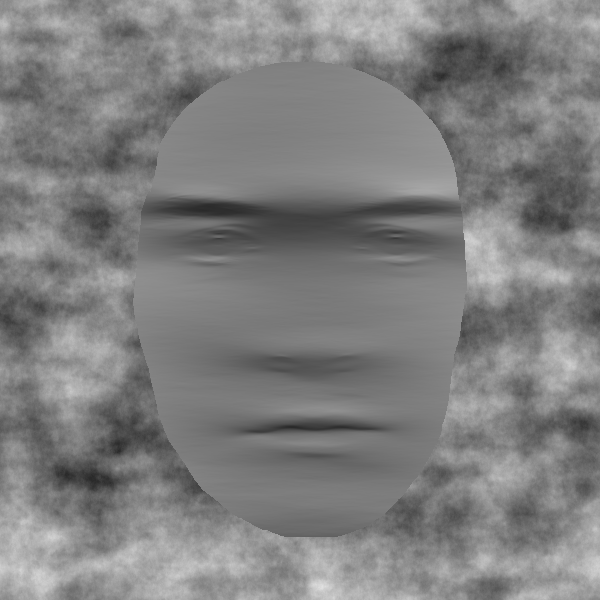

Supplement: S3 File — (ZIP) [file pone.0229185.s003.zip › eq_m17_a.bmp_90.bmp]

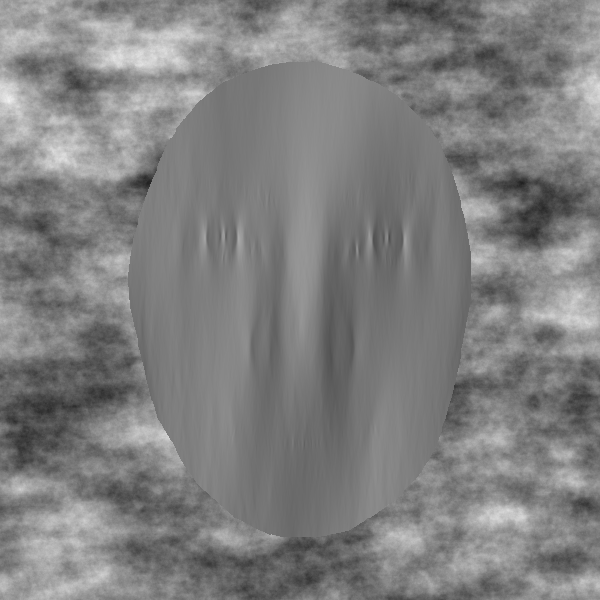

Supplement: S3 File — (ZIP) [file pone.0229185.s003.zip › eq_m18_a.bmp_0.bmp]

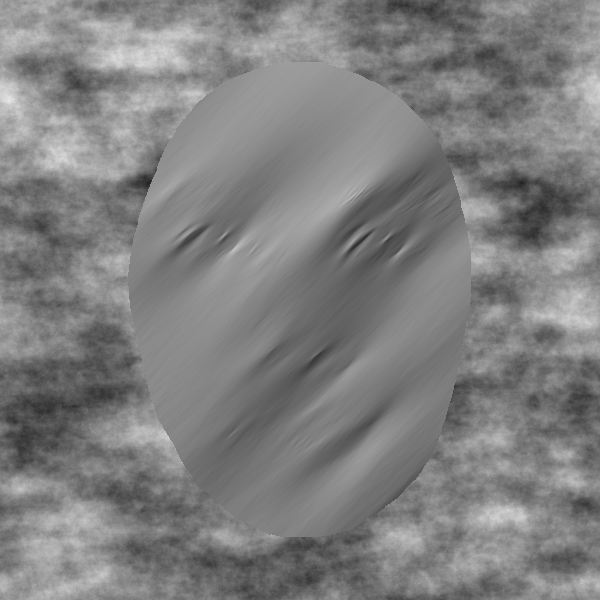

Supplement: S3 File — (ZIP) [file pone.0229185.s003.zip › eq_m18_a.bmp_135.bmp]

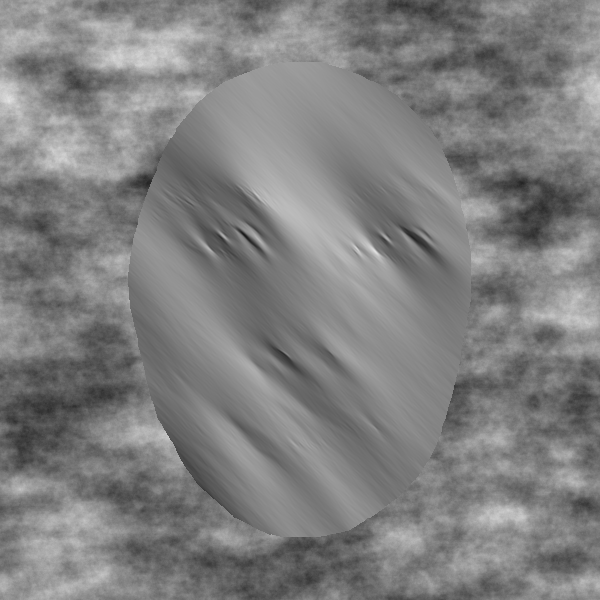

Supplement: S3 File — (ZIP) [file pone.0229185.s003.zip › eq_m18_a.bmp_45.bmp]

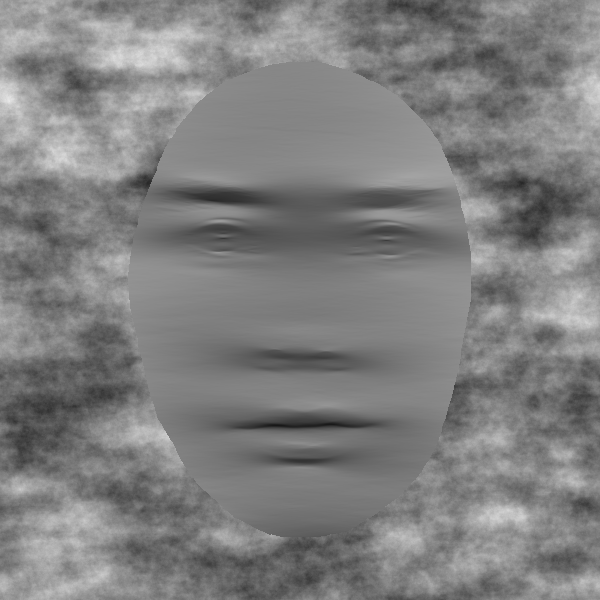

Supplement: S3 File — (ZIP) [file pone.0229185.s003.zip › eq_m18_a.bmp_90.bmp]

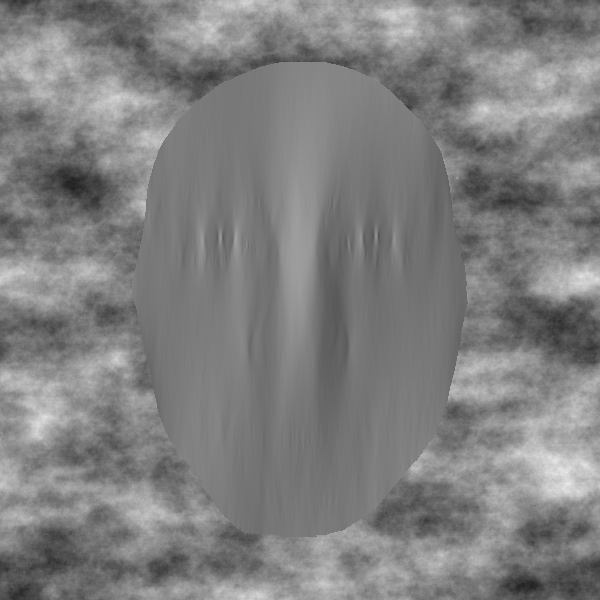

Supplement: S3 File — (ZIP) [file pone.0229185.s003.zip › eq_m19_a.bmp_0.bmp]

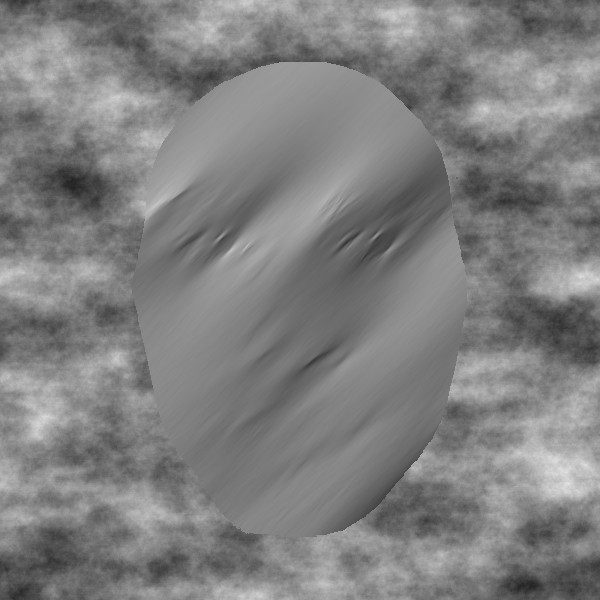

Supplement: S3 File — (ZIP) [file pone.0229185.s003.zip › eq_m19_a.bmp_135.bmp]

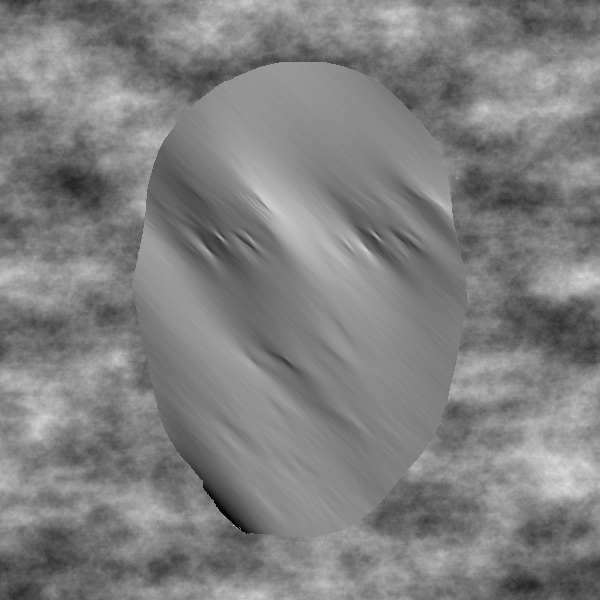

Supplement: S3 File — (ZIP) [file pone.0229185.s003.zip › eq_m19_a.bmp_45.bmp]

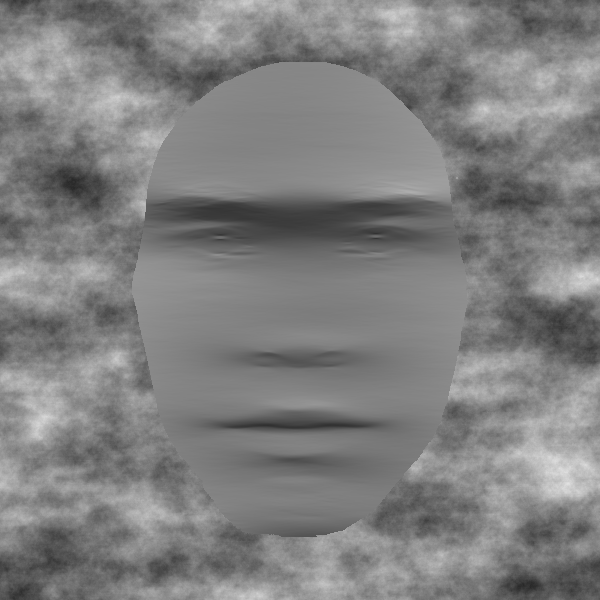

Supplement: S3 File — (ZIP) [file pone.0229185.s003.zip › eq_m19_a.bmp_90.bmp]

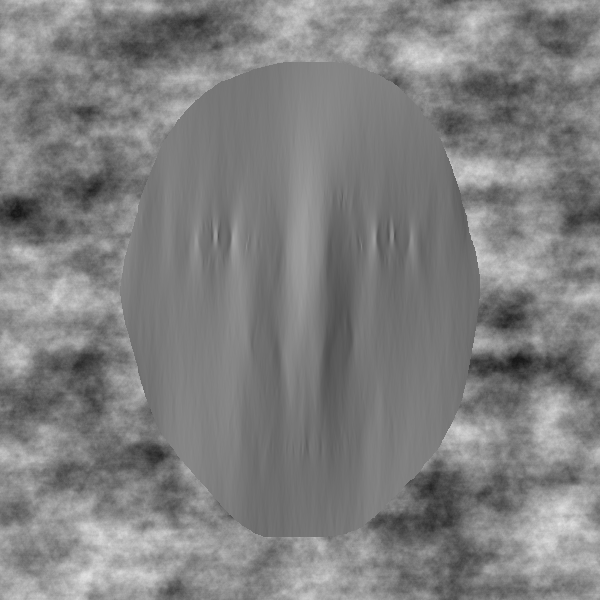

Supplement: S3 File — (ZIP) [file pone.0229185.s003.zip › eq_m20_a.bmp_0.bmp]

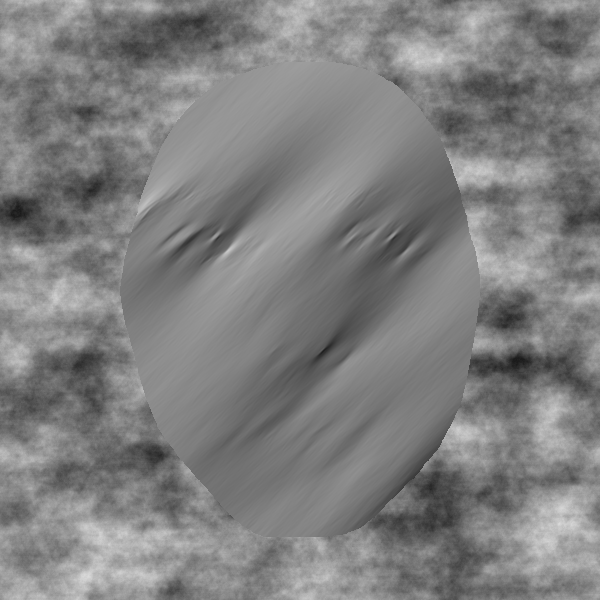

Supplement: S3 File — (ZIP) [file pone.0229185.s003.zip › eq_m20_a.bmp_135.bmp]

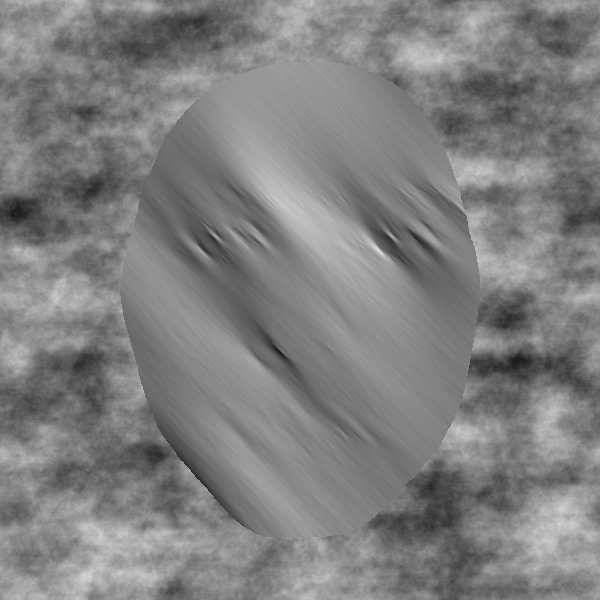

Supplement: S3 File — (ZIP) [file pone.0229185.s003.zip › eq_m20_a.bmp_45.bmp]

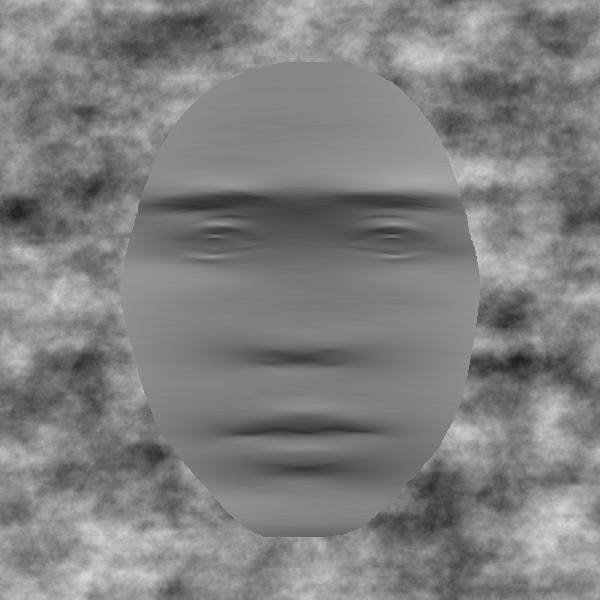

Supplement: S3 File — (ZIP) [file pone.0229185.s003.zip › eq_m20_a.bmp_90.bmp]

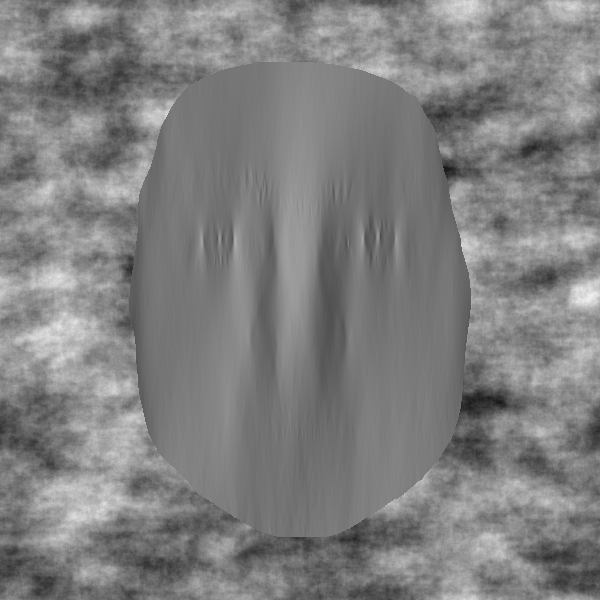

Supplement: S3 File — (ZIP) [file pone.0229185.s003.zip › eq_m21_a.bmp_0.bmp]

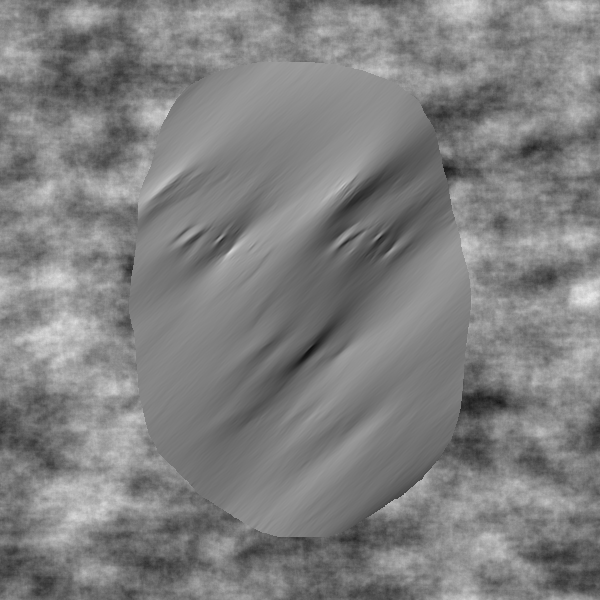

Supplement: S3 File — (ZIP) [file pone.0229185.s003.zip › eq_m21_a.bmp_135.bmp]

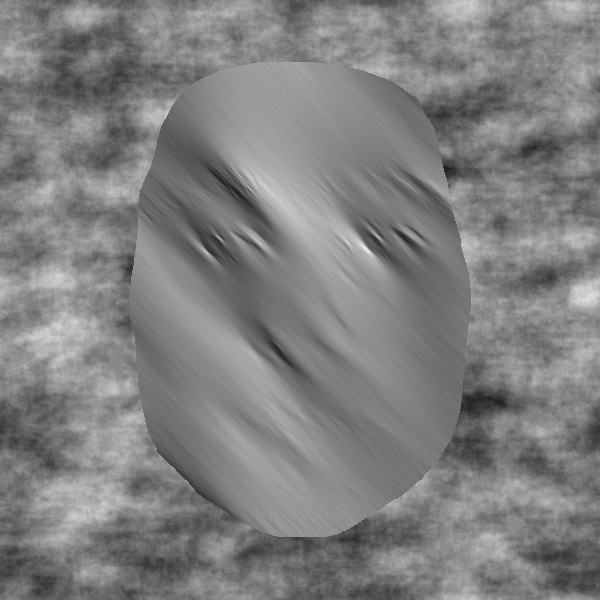

Supplement: S3 File — (ZIP) [file pone.0229185.s003.zip › eq_m21_a.bmp_45.bmp]

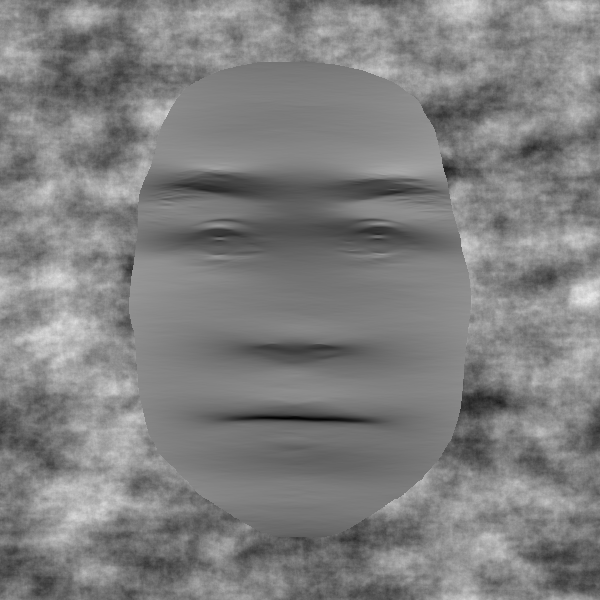

Supplement: S3 File — (ZIP) [file pone.0229185.s003.zip › eq_m21_a.bmp_90.bmp]
