# Supplementary figures and images for: Contrast versus identity encoding in the face image follow distinct orientation selectivity profiles (part 1 of 2)
Source: PLoS One. 2020 Mar 18;15(3):e0229185. doi: 10.1371/journal.pone.0229185 (PMC7080280; doi:10.1371/journal.pone.0229185)

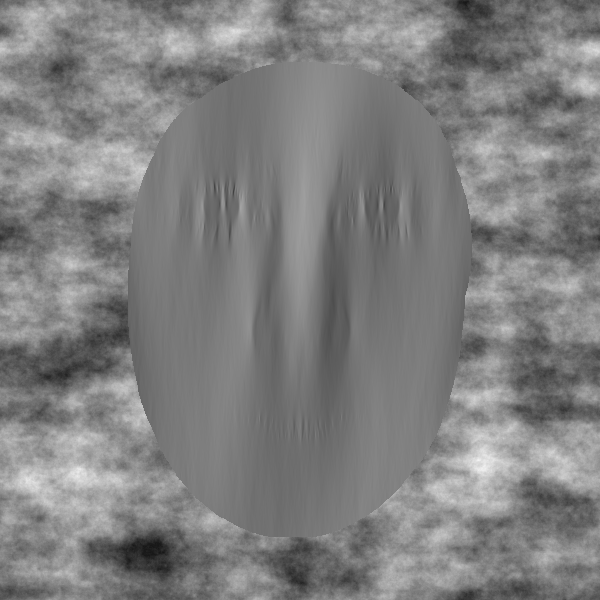

Supplement: S3 File — (ZIP) [file pone.0229185.s003.zip › eq_f02_a.bmp_0.bmp]

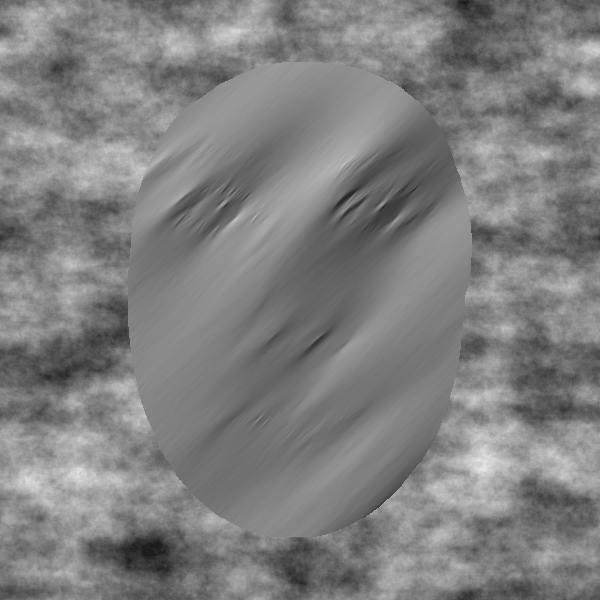

Supplement: S3 File — (ZIP) [file pone.0229185.s003.zip › eq_f02_a.bmp_135.bmp]

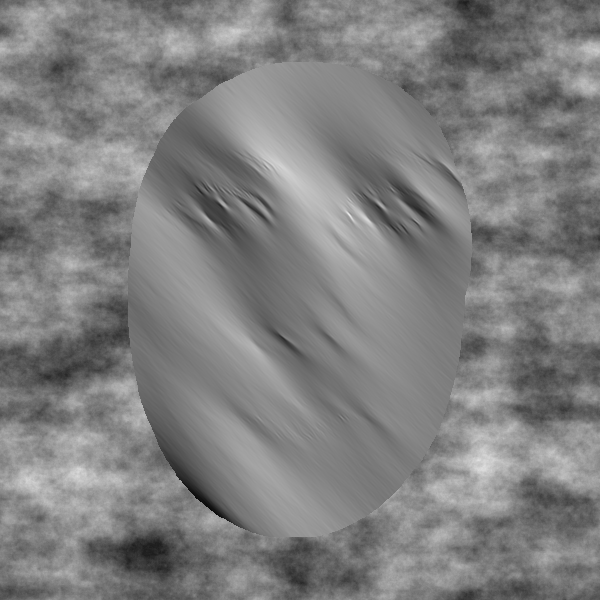

Supplement: S3 File — (ZIP) [file pone.0229185.s003.zip › eq_f02_a.bmp_45.bmp]

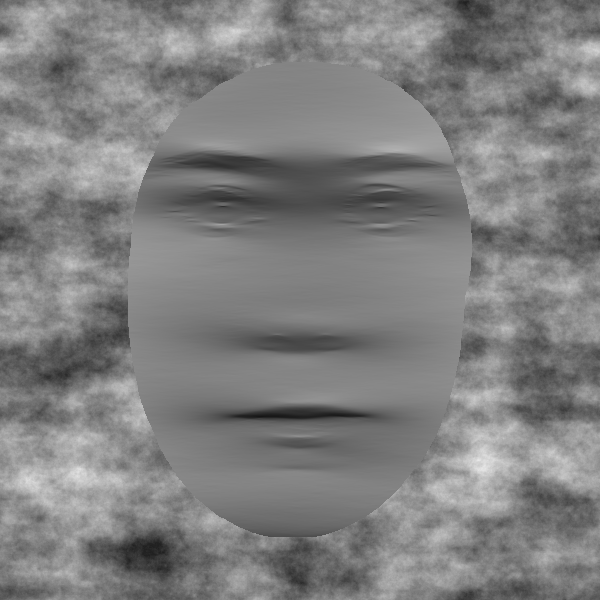

Supplement: S3 File — (ZIP) [file pone.0229185.s003.zip › eq_f02_a.bmp_90.bmp]

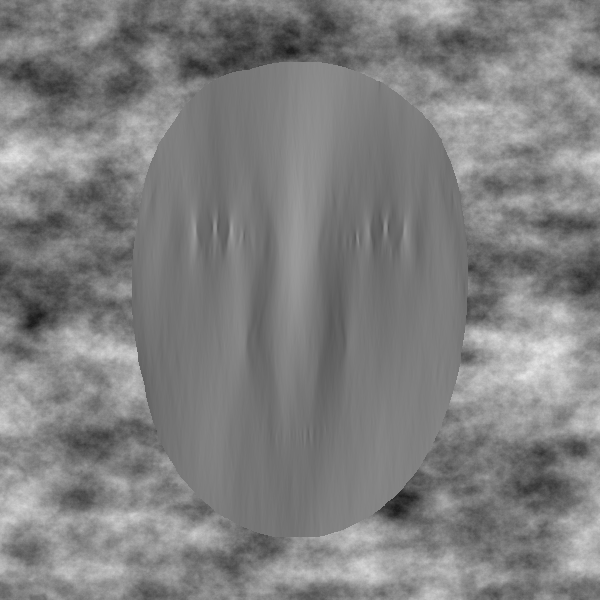

Supplement: S3 File — (ZIP) [file pone.0229185.s003.zip › eq_f04_a.bmp_0.bmp]

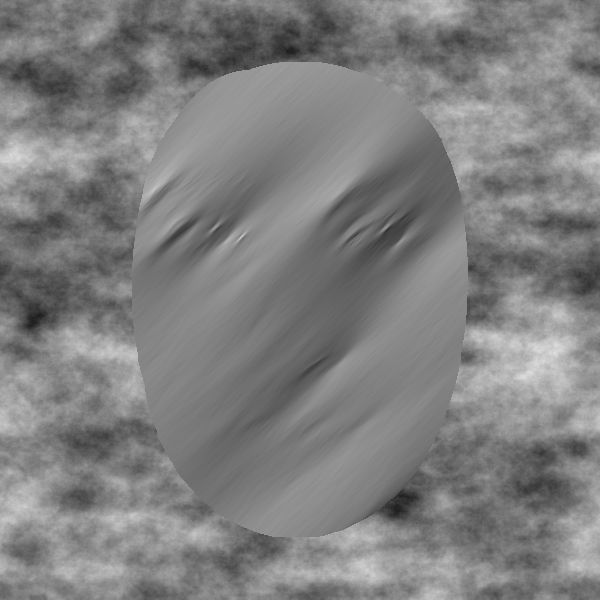

Supplement: S3 File — (ZIP) [file pone.0229185.s003.zip › eq_f04_a.bmp_135.bmp]

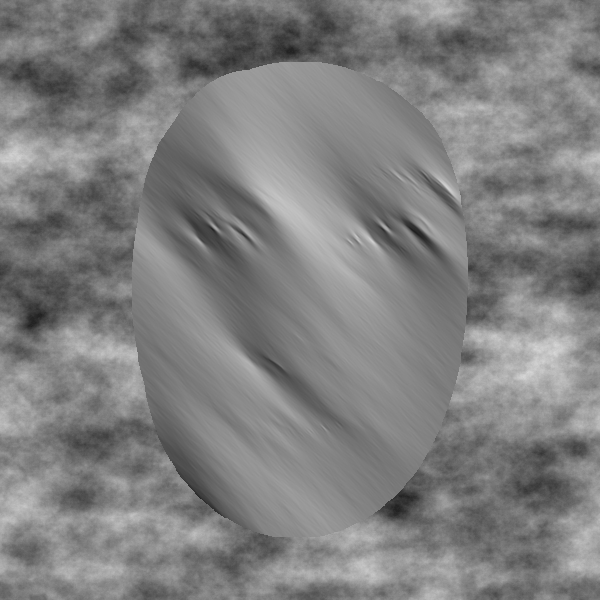

Supplement: S3 File — (ZIP) [file pone.0229185.s003.zip › eq_f04_a.bmp_45.bmp]

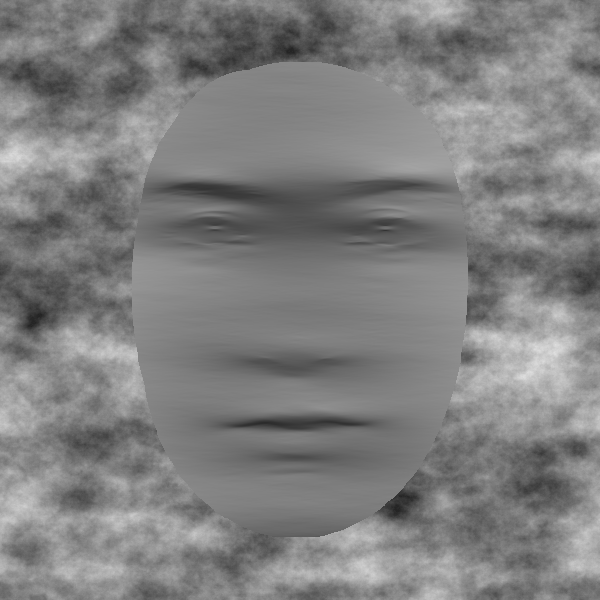

Supplement: S3 File — (ZIP) [file pone.0229185.s003.zip › eq_f04_a.bmp_90.bmp]

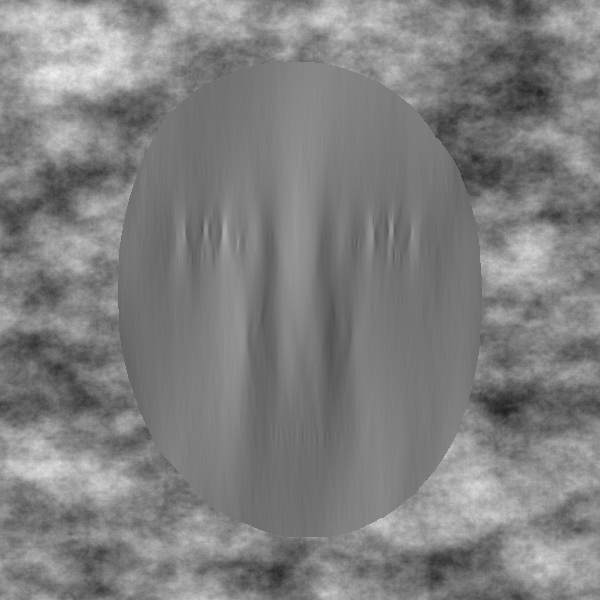

Supplement: S3 File — (ZIP) [file pone.0229185.s003.zip › eq_f06_a.bmp_0.bmp]

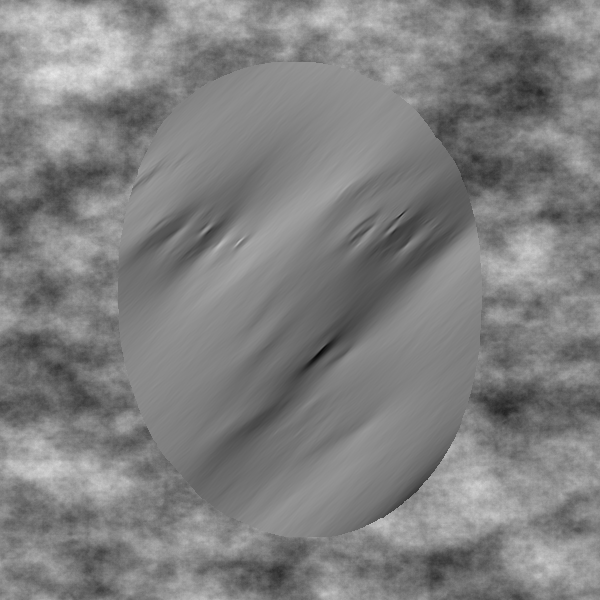

Supplement: S3 File — (ZIP) [file pone.0229185.s003.zip › eq_f06_a.bmp_135.bmp]

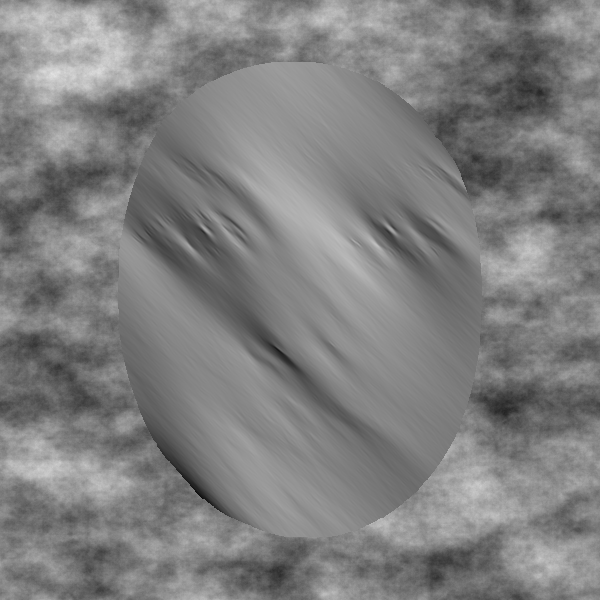

Supplement: S3 File — (ZIP) [file pone.0229185.s003.zip › eq_f06_a.bmp_45.bmp]

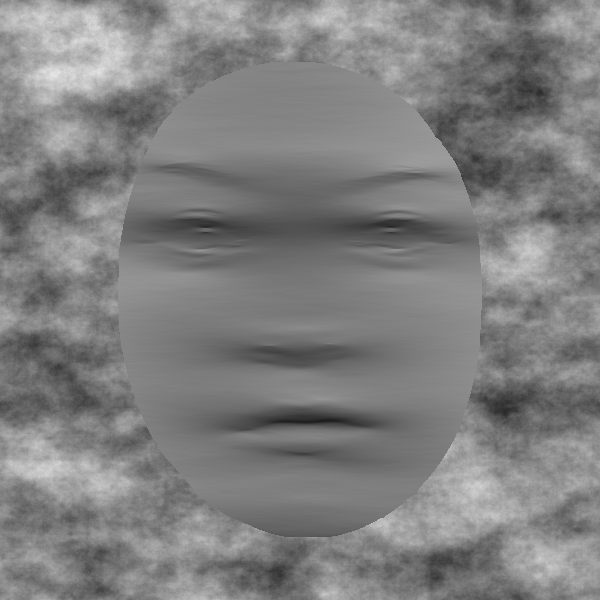

Supplement: S3 File — (ZIP) [file pone.0229185.s003.zip › eq_f06_a.bmp_90.bmp]

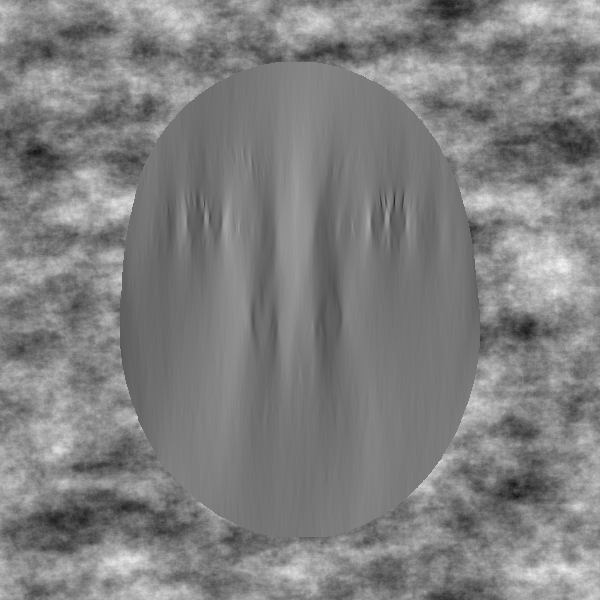

Supplement: S3 File — (ZIP) [file pone.0229185.s003.zip › eq_f07_a.bmp_0.bmp]

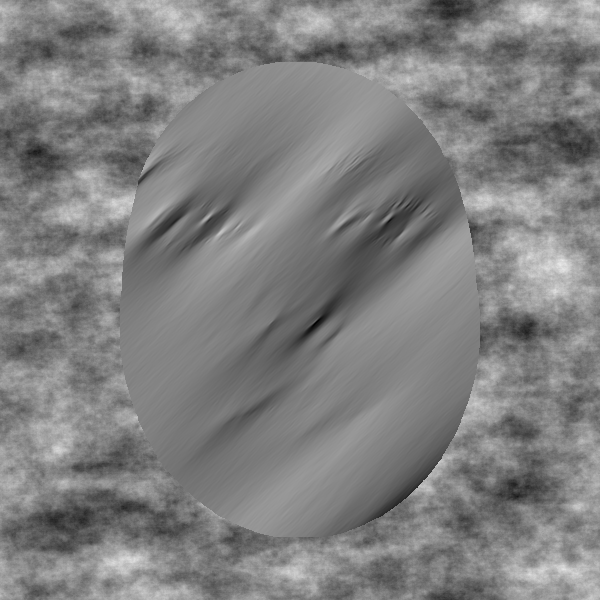

Supplement: S3 File — (ZIP) [file pone.0229185.s003.zip › eq_f07_a.bmp_135.bmp]

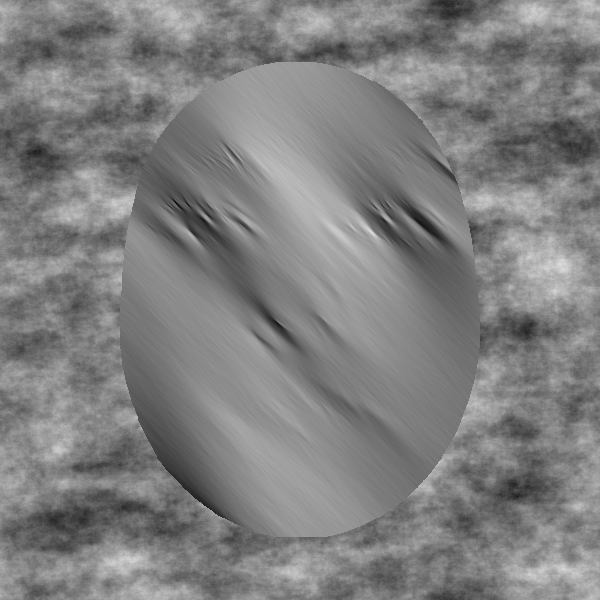

Supplement: S3 File — (ZIP) [file pone.0229185.s003.zip › eq_f07_a.bmp_45.bmp]

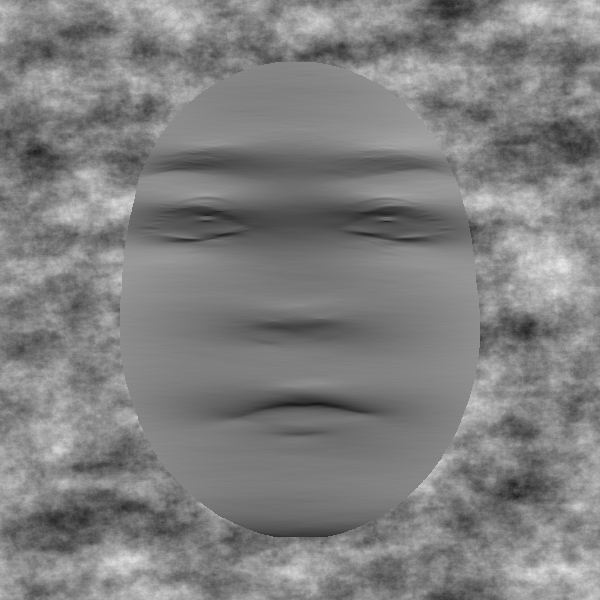

Supplement: S3 File — (ZIP) [file pone.0229185.s003.zip › eq_f07_a.bmp_90.bmp]

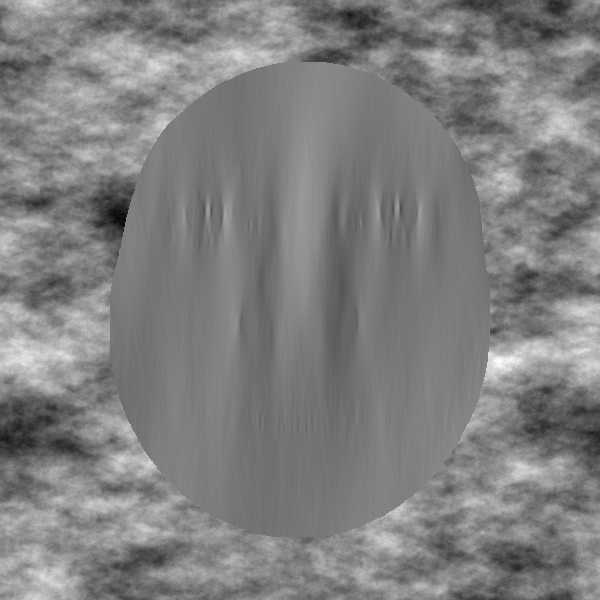

Supplement: S3 File — (ZIP) [file pone.0229185.s003.zip › eq_f08_a.bmp_0.bmp]

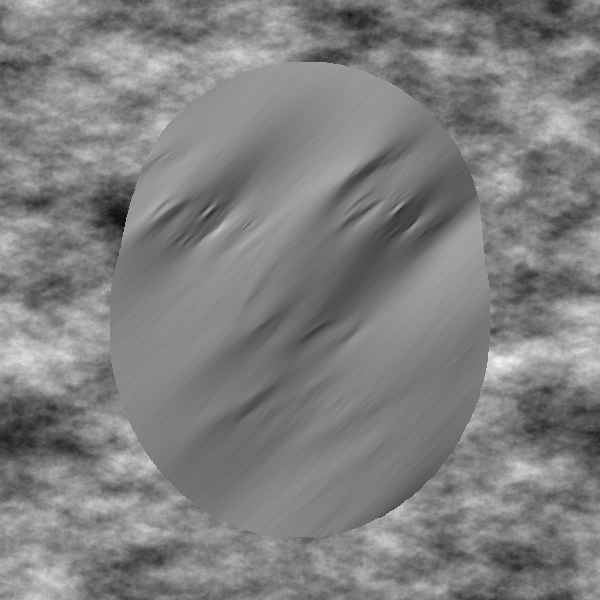

Supplement: S3 File — (ZIP) [file pone.0229185.s003.zip › eq_f08_a.bmp_135.bmp]

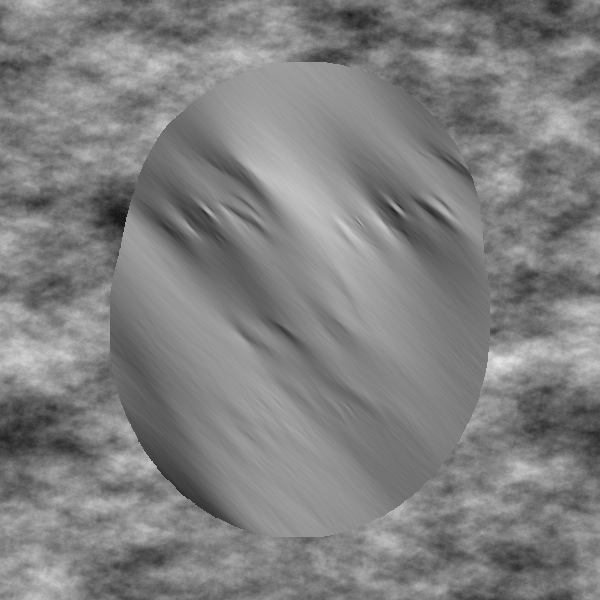

Supplement: S3 File — (ZIP) [file pone.0229185.s003.zip › eq_f08_a.bmp_45.bmp]

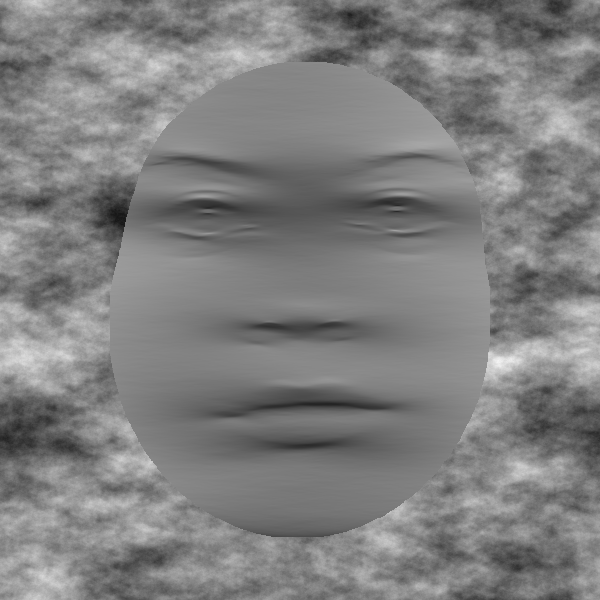

Supplement: S3 File — (ZIP) [file pone.0229185.s003.zip › eq_f08_a.bmp_90.bmp]

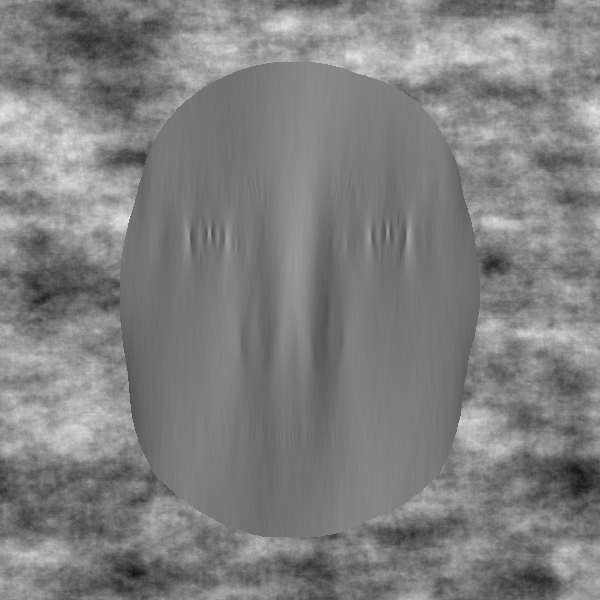

Supplement: S3 File — (ZIP) [file pone.0229185.s003.zip › eq_f10_a.bmp_0.bmp]

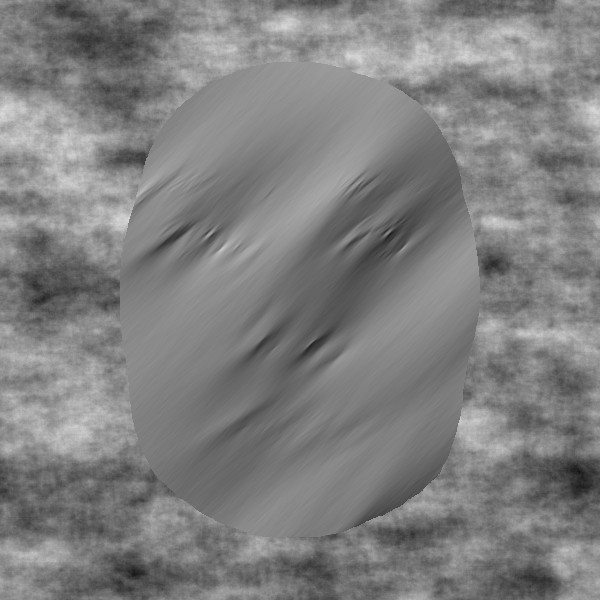

Supplement: S3 File — (ZIP) [file pone.0229185.s003.zip › eq_f10_a.bmp_135.bmp]

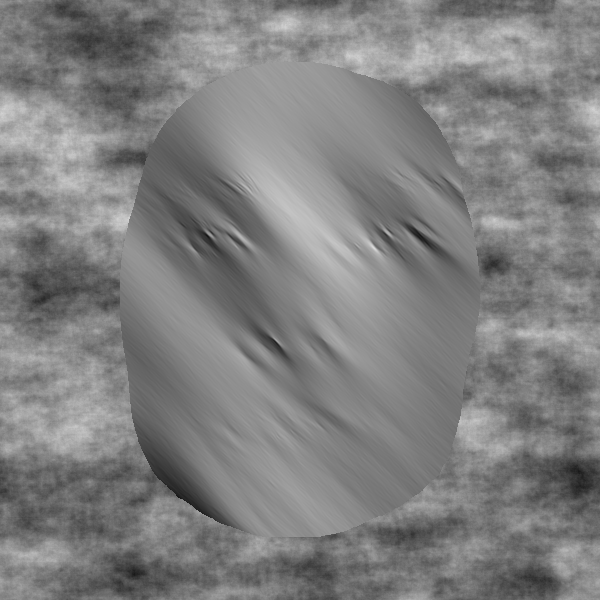

Supplement: S3 File — (ZIP) [file pone.0229185.s003.zip › eq_f10_a.bmp_45.bmp]

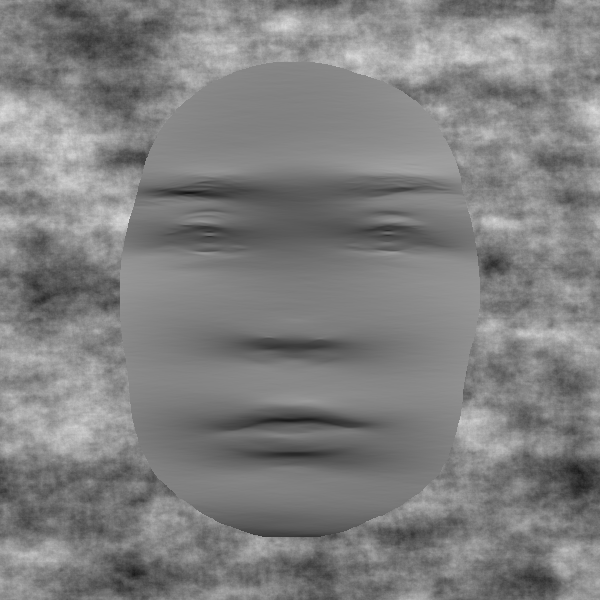

Supplement: S3 File — (ZIP) [file pone.0229185.s003.zip › eq_f10_a.bmp_90.bmp]

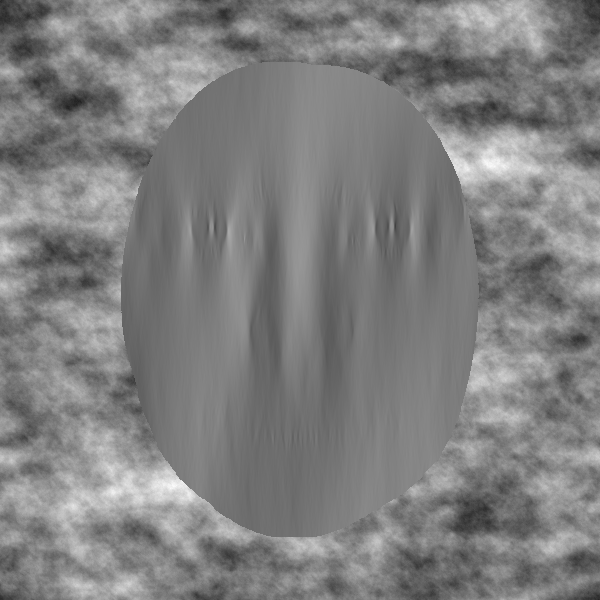

Supplement: S3 File — (ZIP) [file pone.0229185.s003.zip › eq_f12_a.bmp_0.bmp]

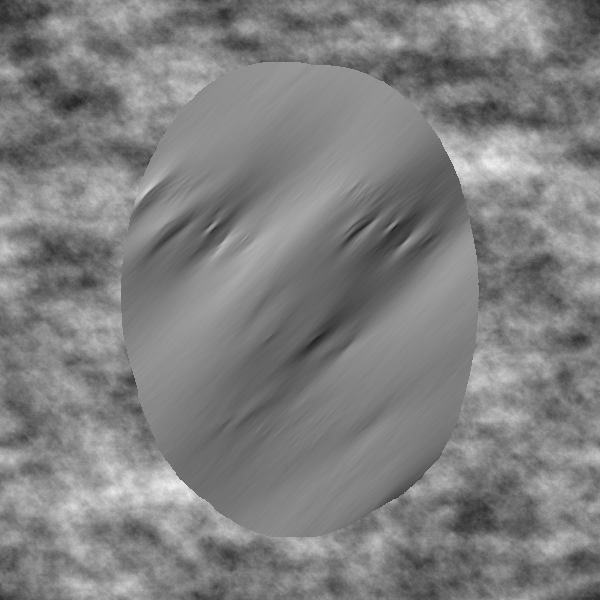

Supplement: S3 File — (ZIP) [file pone.0229185.s003.zip › eq_f12_a.bmp_135.bmp]

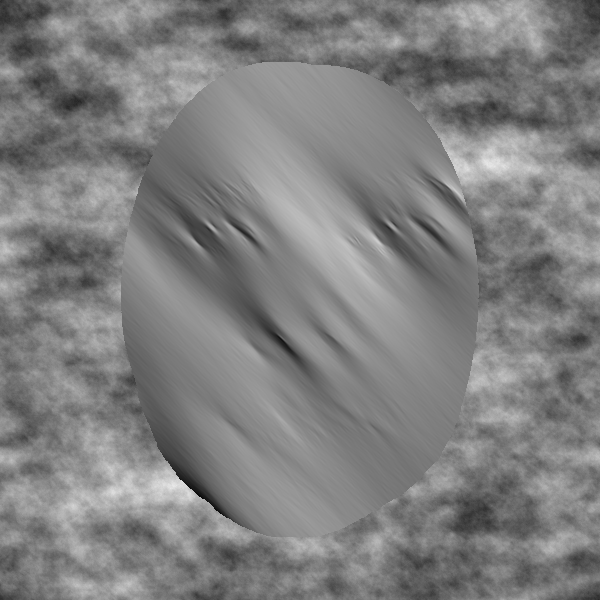

Supplement: S3 File — (ZIP) [file pone.0229185.s003.zip › eq_f12_a.bmp_45.bmp]

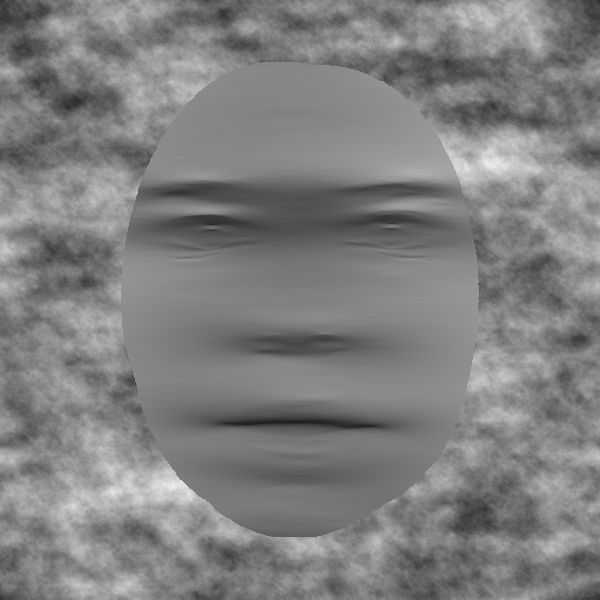

Supplement: S3 File — (ZIP) [file pone.0229185.s003.zip › eq_f12_a.bmp_90.bmp]

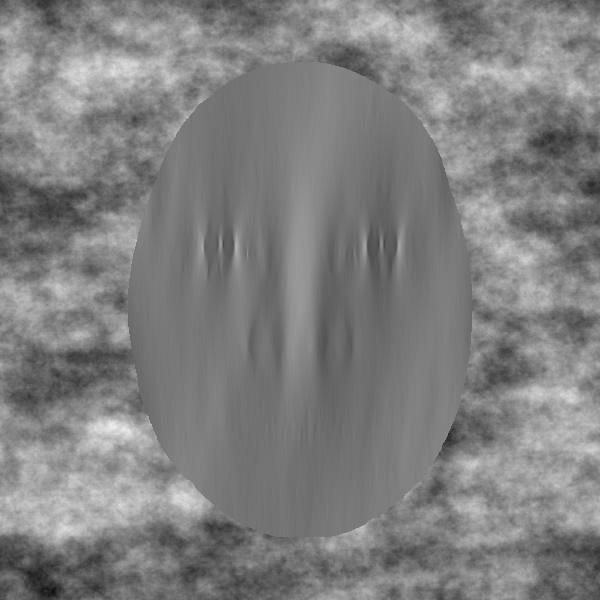

Supplement: S3 File — (ZIP) [file pone.0229185.s003.zip › eq_f13_a.bmp_0.bmp]

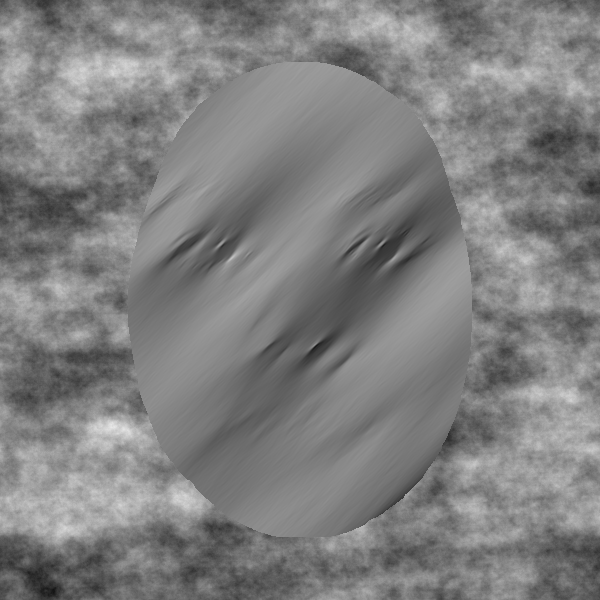

Supplement: S3 File — (ZIP) [file pone.0229185.s003.zip › eq_f13_a.bmp_135.bmp]

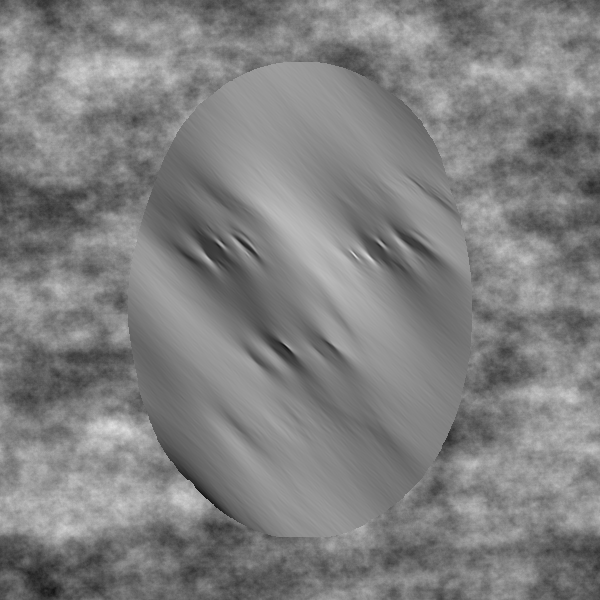

Supplement: S3 File — (ZIP) [file pone.0229185.s003.zip › eq_f13_a.bmp_45.bmp]

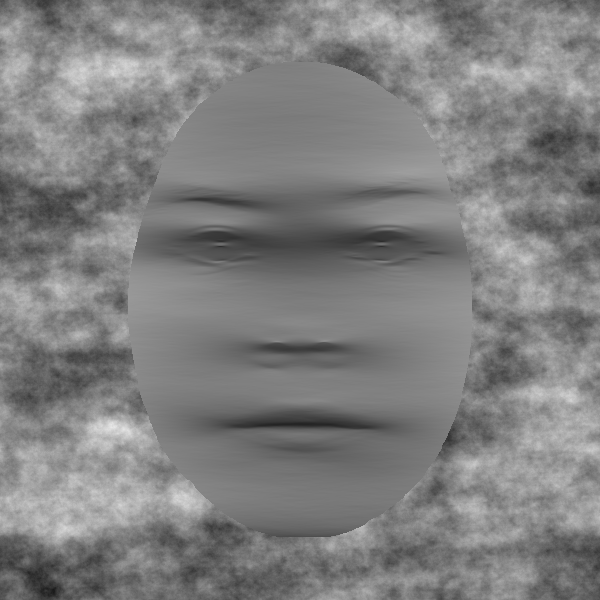

Supplement: S3 File — (ZIP) [file pone.0229185.s003.zip › eq_f13_a.bmp_90.bmp]

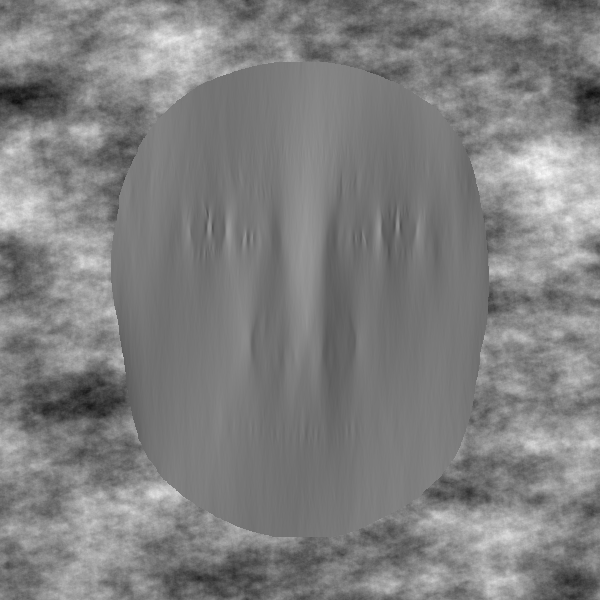

Supplement: S3 File — (ZIP) [file pone.0229185.s003.zip › eq_f14_a.bmp_0.bmp]

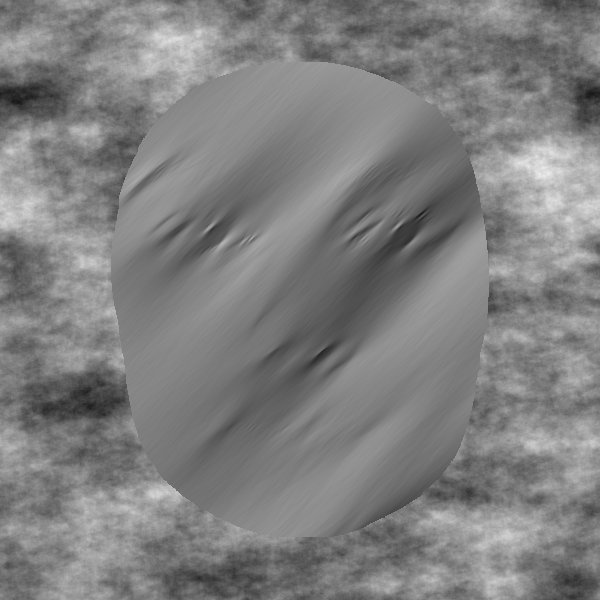

Supplement: S3 File — (ZIP) [file pone.0229185.s003.zip › eq_f14_a.bmp_135.bmp]

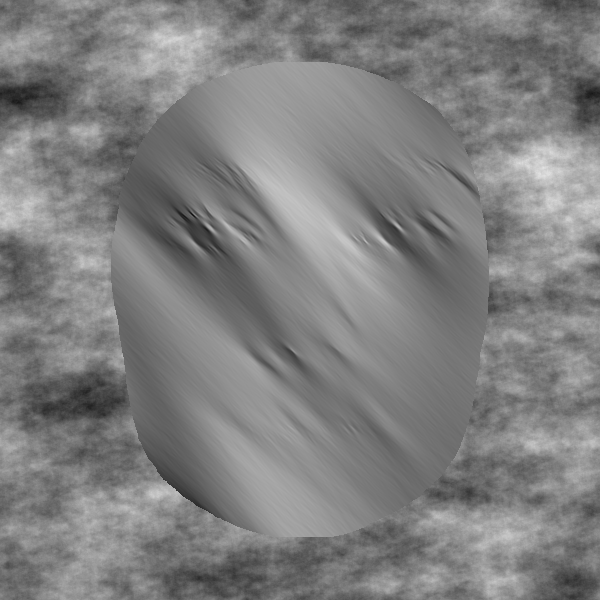

Supplement: S3 File — (ZIP) [file pone.0229185.s003.zip › eq_f14_a.bmp_45.bmp]

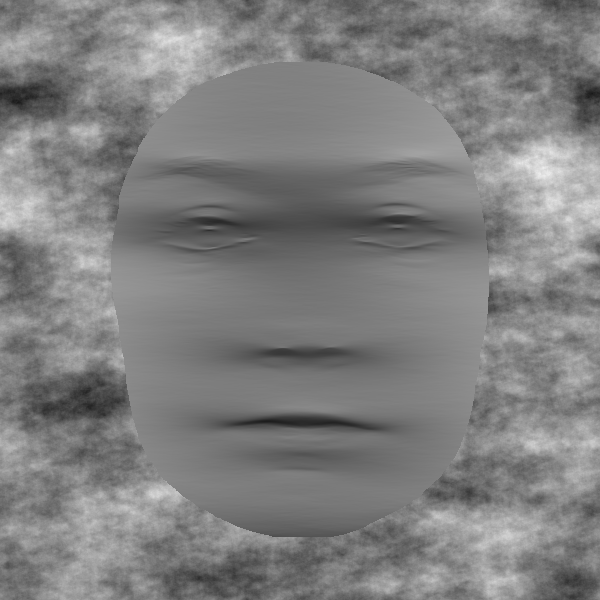

Supplement: S3 File — (ZIP) [file pone.0229185.s003.zip › eq_f14_a.bmp_90.bmp]

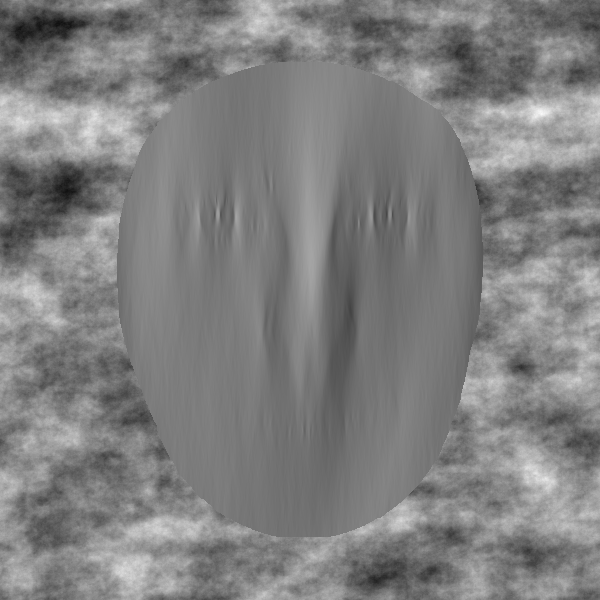

Supplement: S3 File — (ZIP) [file pone.0229185.s003.zip › eq_f15_a.bmp_0.bmp]

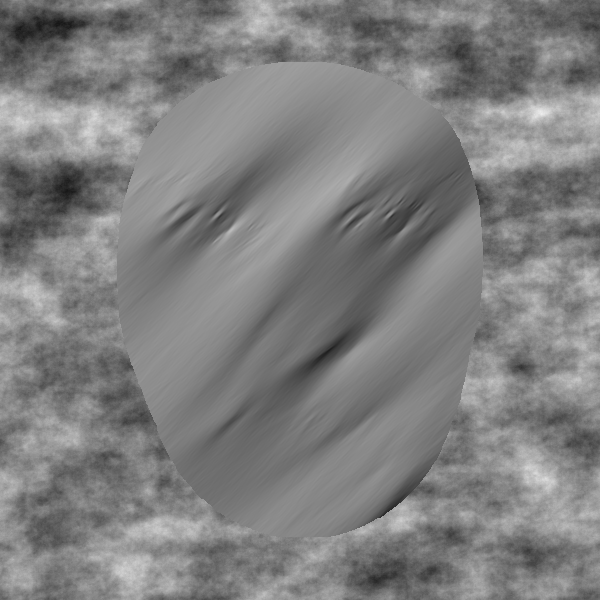

Supplement: S3 File — (ZIP) [file pone.0229185.s003.zip › eq_f15_a.bmp_135.bmp]

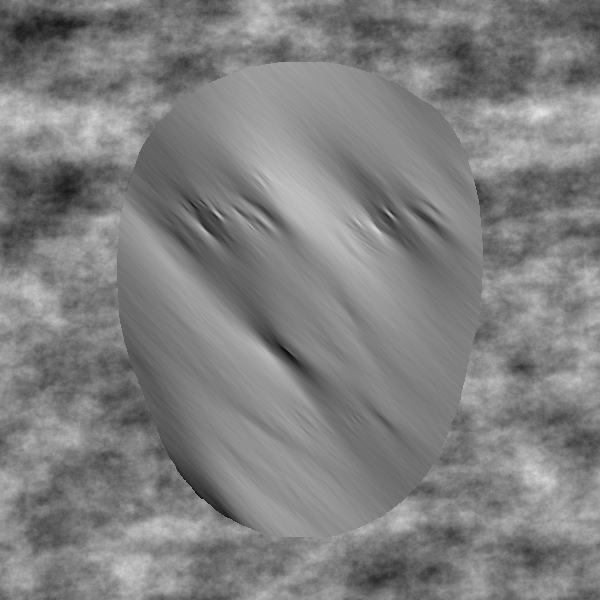

Supplement: S3 File — (ZIP) [file pone.0229185.s003.zip › eq_f15_a.bmp_45.bmp]

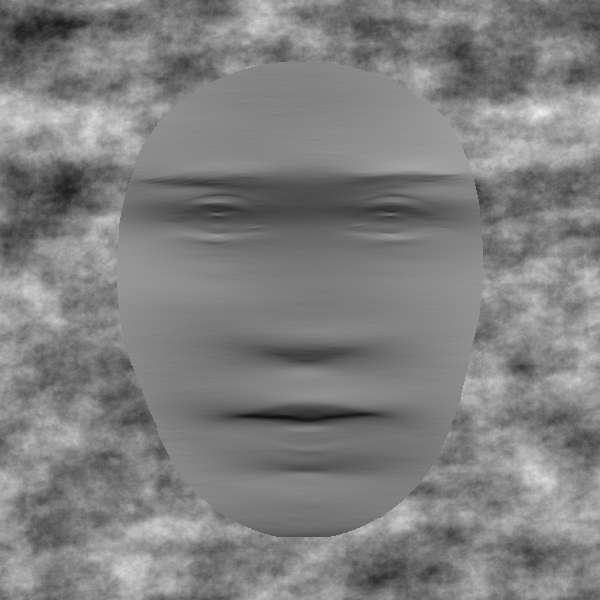

Supplement: S3 File — (ZIP) [file pone.0229185.s003.zip › eq_f15_a.bmp_90.bmp]

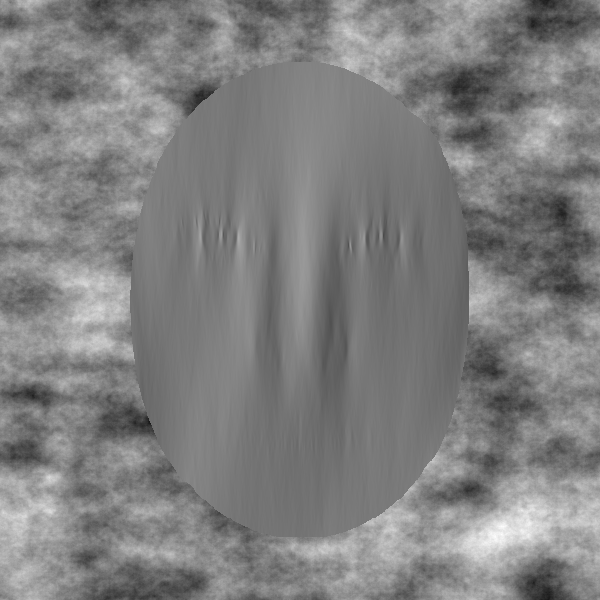

Supplement: S3 File — (ZIP) [file pone.0229185.s003.zip › eq_f16_a.bmp_0.bmp]

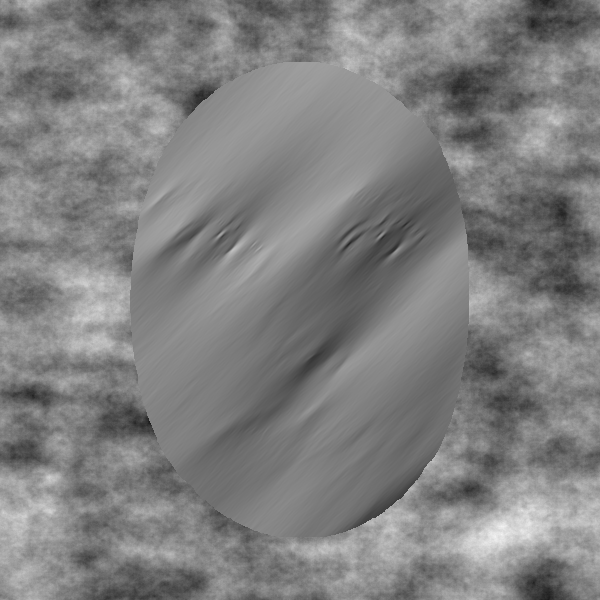

Supplement: S3 File — (ZIP) [file pone.0229185.s003.zip › eq_f16_a.bmp_135.bmp]

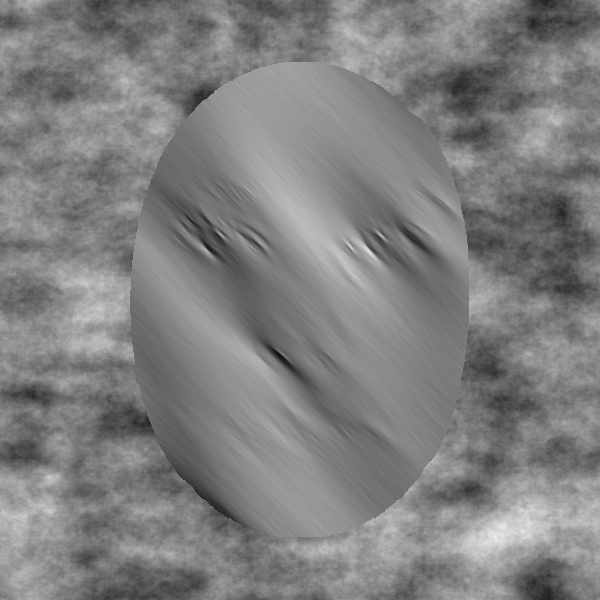

Supplement: S3 File — (ZIP) [file pone.0229185.s003.zip › eq_f16_a.bmp_45.bmp]

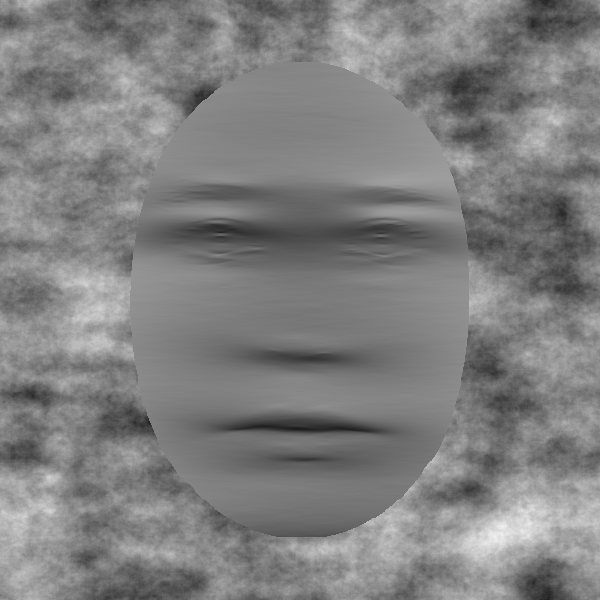

Supplement: S3 File — (ZIP) [file pone.0229185.s003.zip › eq_f16_a.bmp_90.bmp]

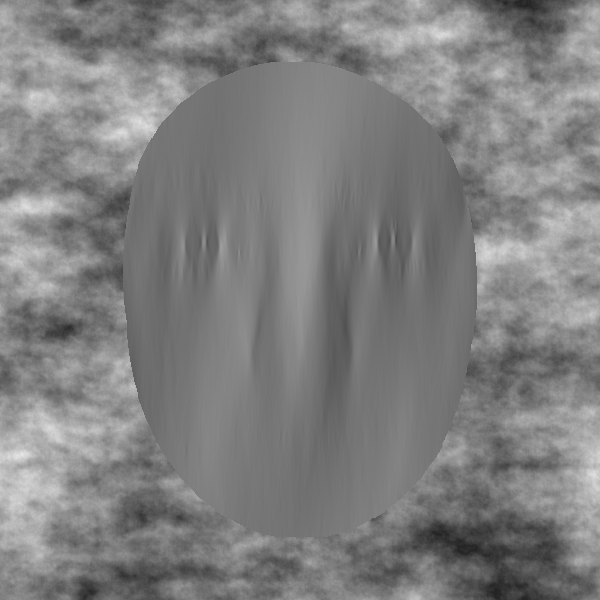

Supplement: S3 File — (ZIP) [file pone.0229185.s003.zip › eq_f17_a.bmp_0.bmp]

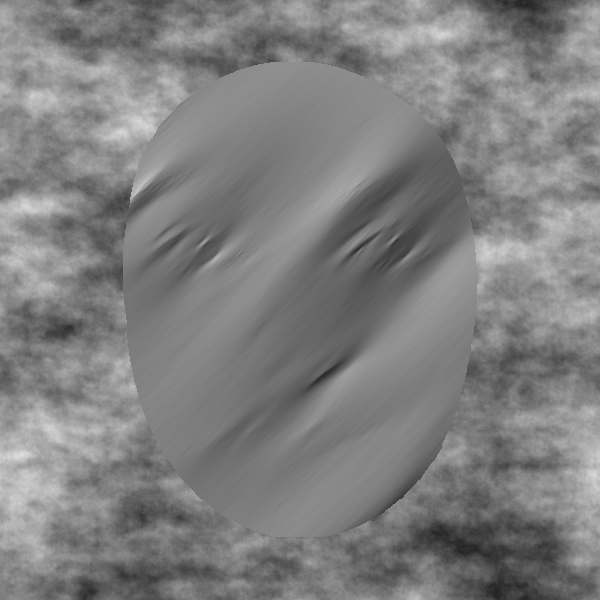

Supplement: S3 File — (ZIP) [file pone.0229185.s003.zip › eq_f17_a.bmp_135.bmp]

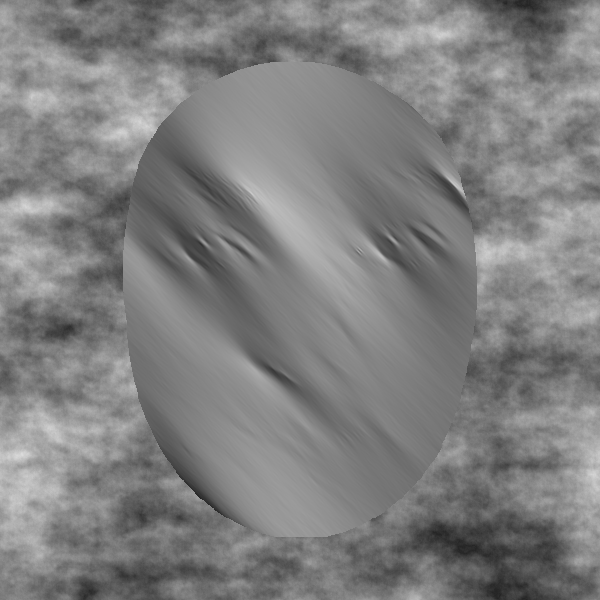

Supplement: S3 File — (ZIP) [file pone.0229185.s003.zip › eq_f17_a.bmp_45.bmp]

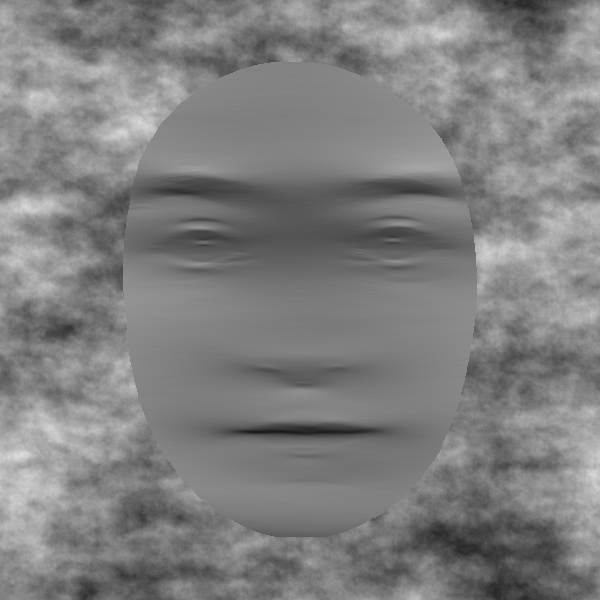

Supplement: S3 File — (ZIP) [file pone.0229185.s003.zip › eq_f17_a.bmp_90.bmp]

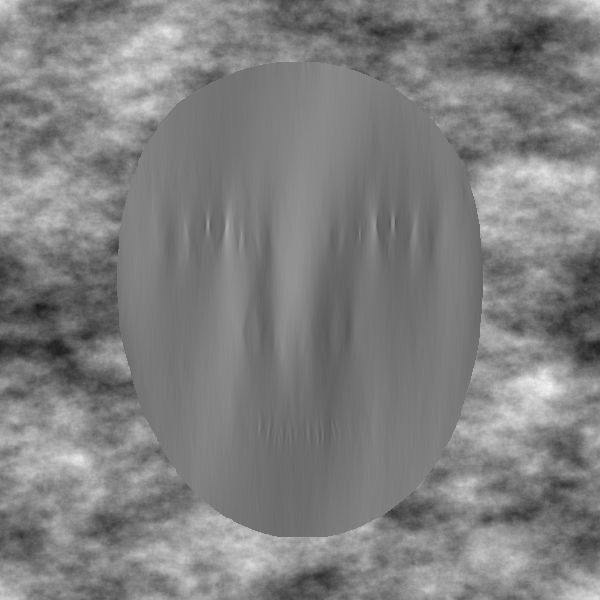

Supplement: S3 File — (ZIP) [file pone.0229185.s003.zip › eq_f19_a.bmp_0.bmp]

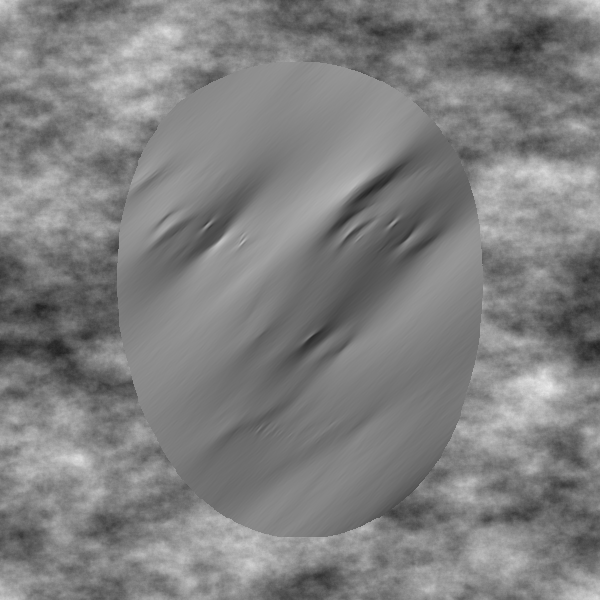

Supplement: S3 File — (ZIP) [file pone.0229185.s003.zip › eq_f19_a.bmp_135.bmp]

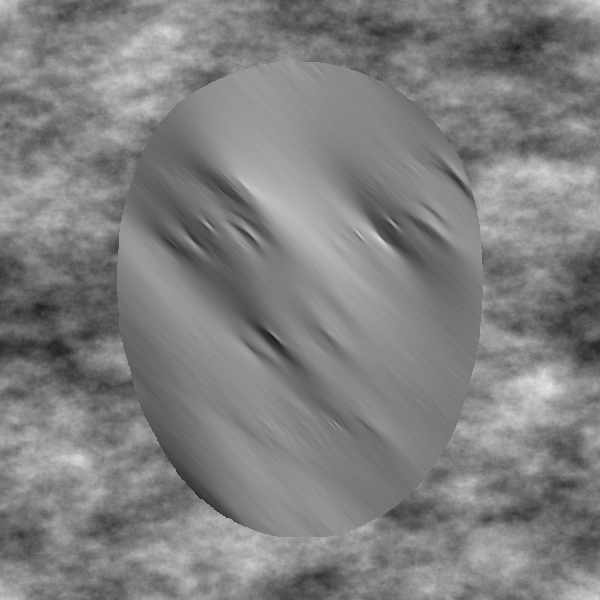

Supplement: S3 File — (ZIP) [file pone.0229185.s003.zip › eq_f19_a.bmp_45.bmp]

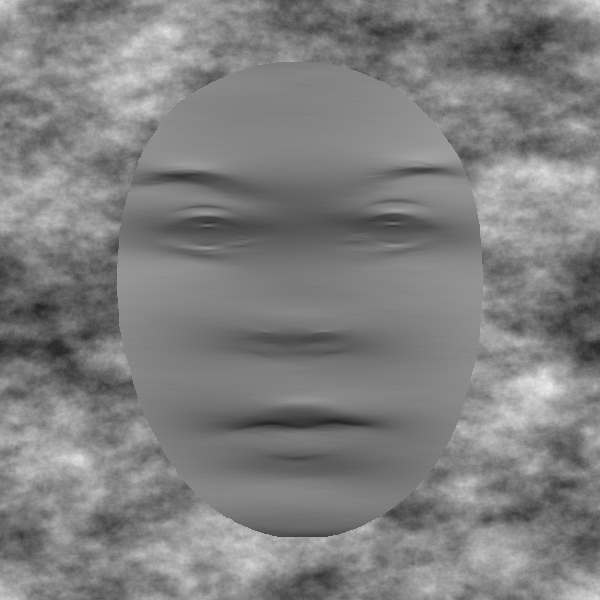

Supplement: S3 File — (ZIP) [file pone.0229185.s003.zip › eq_f19_a.bmp_90.bmp]

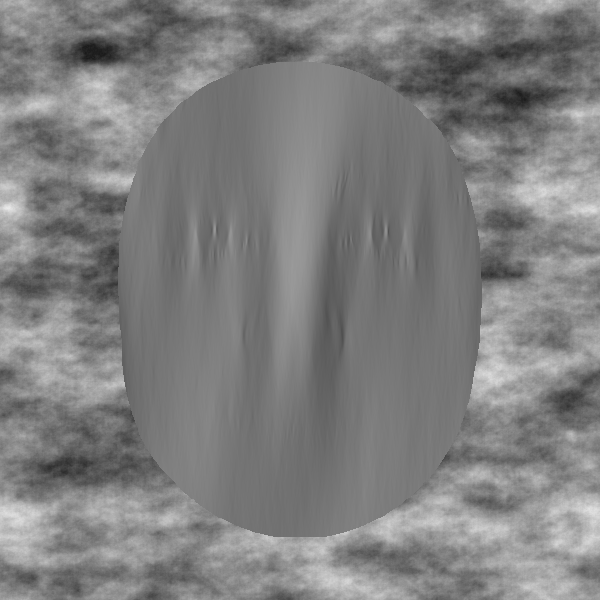

Supplement: S3 File — (ZIP) [file pone.0229185.s003.zip › eq_f20_a.bmp_0.bmp]

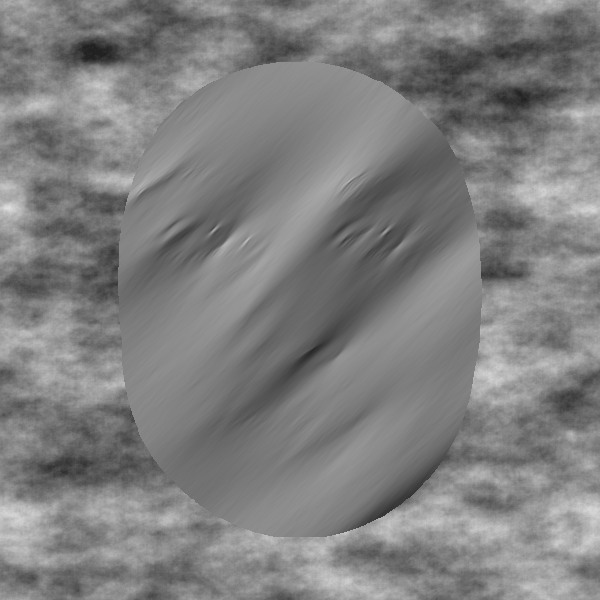

Supplement: S3 File — (ZIP) [file pone.0229185.s003.zip › eq_f20_a.bmp_135.bmp]

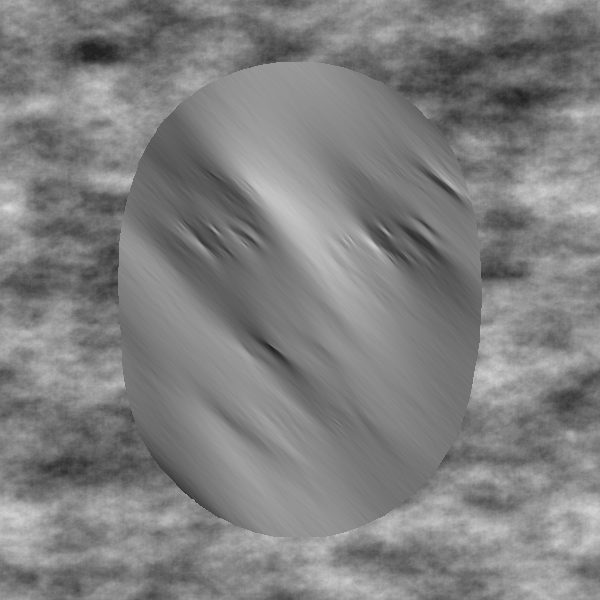

Supplement: S3 File — (ZIP) [file pone.0229185.s003.zip › eq_f20_a.bmp_45.bmp]

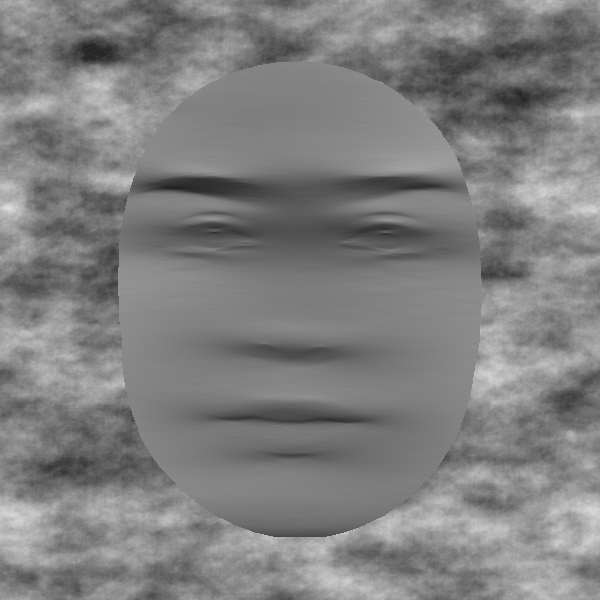

Supplement: S3 File — (ZIP) [file pone.0229185.s003.zip › eq_f20_a.bmp_90.bmp]

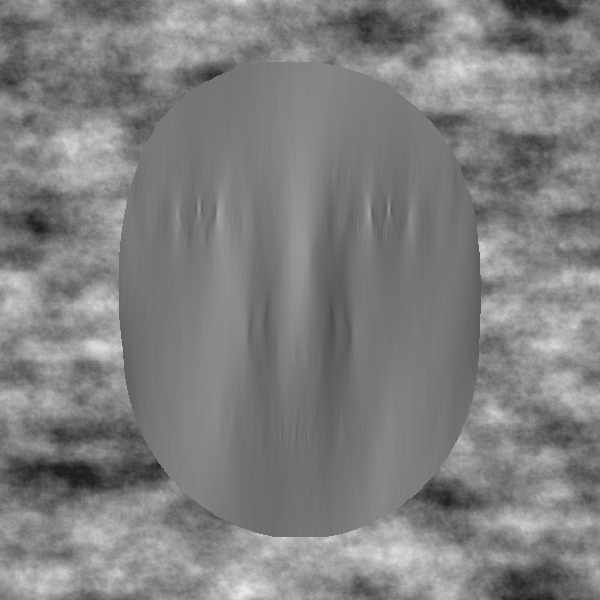

Supplement: S3 File — (ZIP) [file pone.0229185.s003.zip › eq_f21_a.bmp_0.bmp]

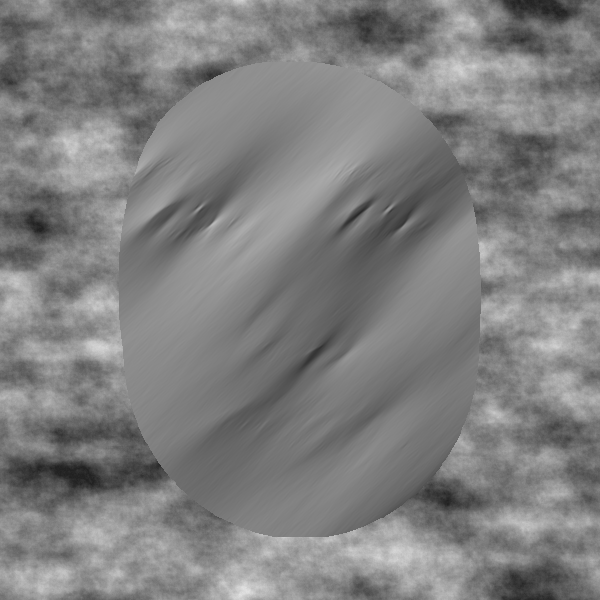

Supplement: S3 File — (ZIP) [file pone.0229185.s003.zip › eq_f21_a.bmp_135.bmp]

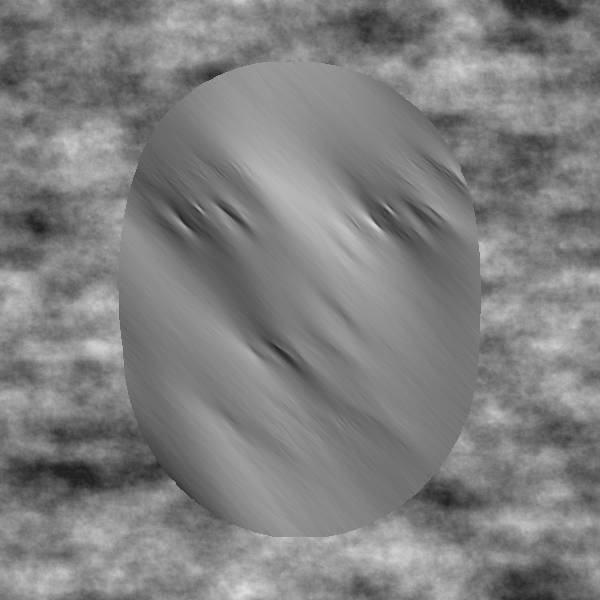

Supplement: S3 File — (ZIP) [file pone.0229185.s003.zip › eq_f21_a.bmp_45.bmp]

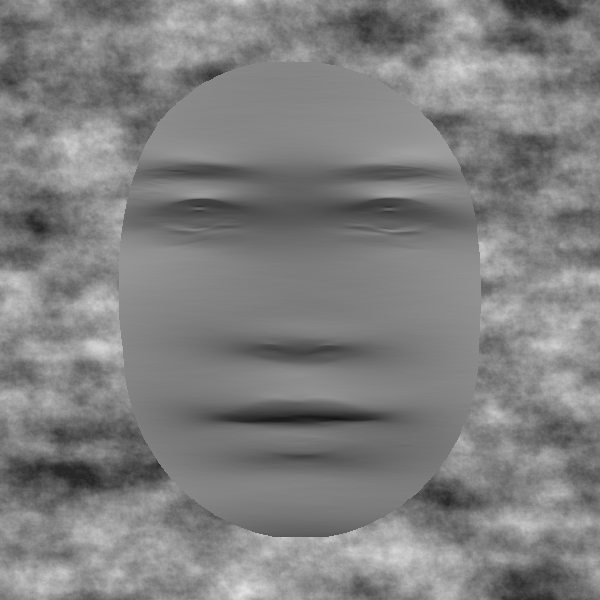

Supplement: S3 File — (ZIP) [file pone.0229185.s003.zip › eq_f21_a.bmp_90.bmp]

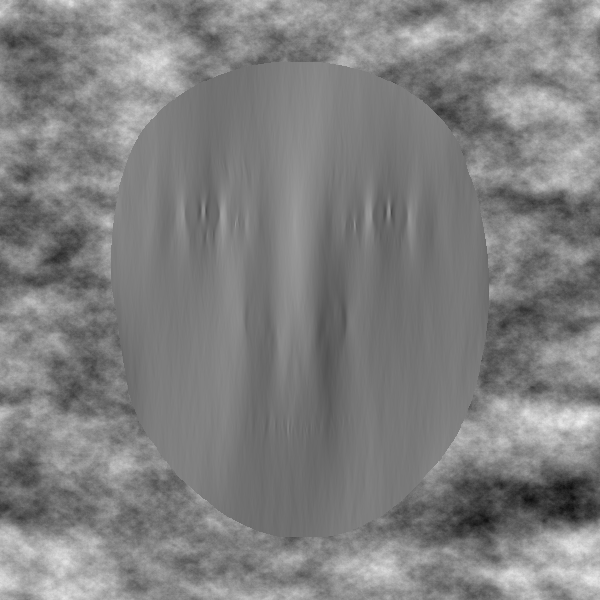

Supplement: S3 File — (ZIP) [file pone.0229185.s003.zip › eq_f22_a.bmp_0.bmp]

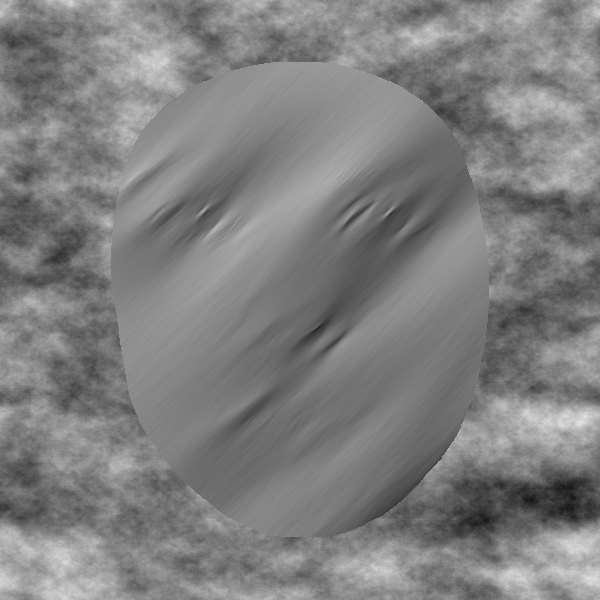

Supplement: S3 File — (ZIP) [file pone.0229185.s003.zip › eq_f22_a.bmp_135.bmp]

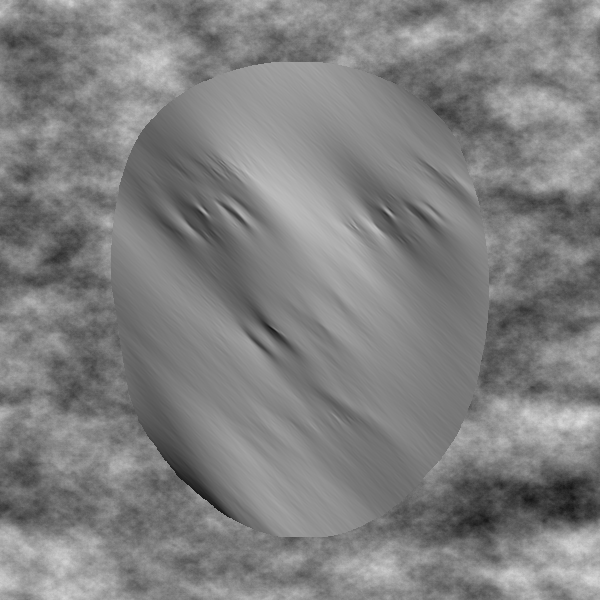

Supplement: S3 File — (ZIP) [file pone.0229185.s003.zip › eq_f22_a.bmp_45.bmp]

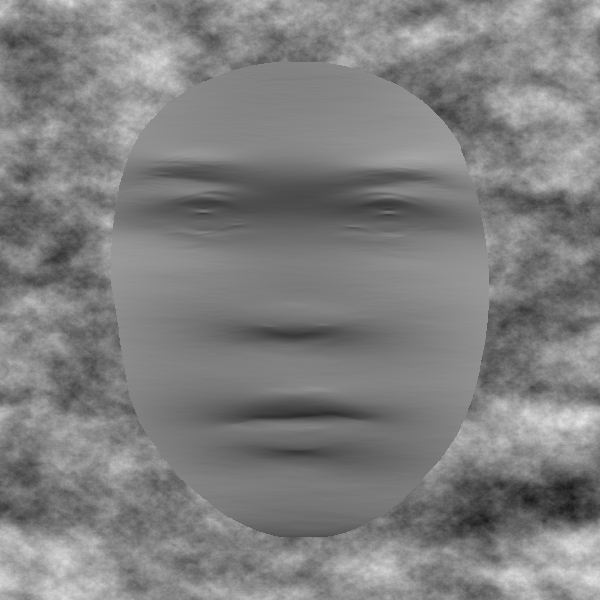

Supplement: S3 File — (ZIP) [file pone.0229185.s003.zip › eq_f22_a.bmp_90.bmp]

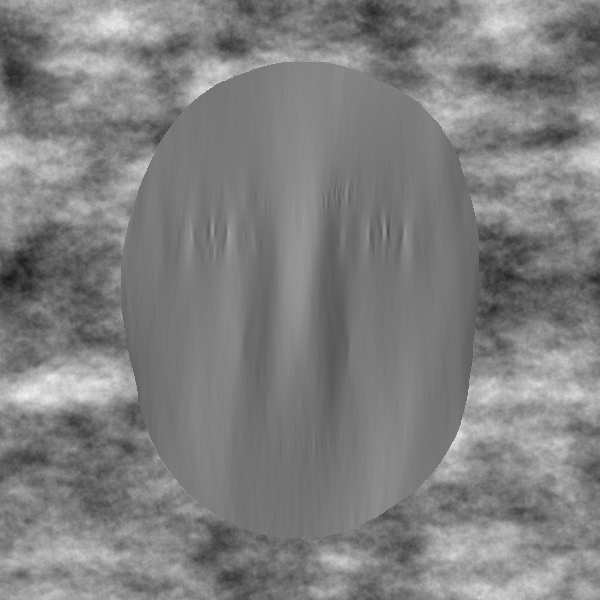

Supplement: S3 File — (ZIP) [file pone.0229185.s003.zip › eq_m04_a.bmp_0.bmp]

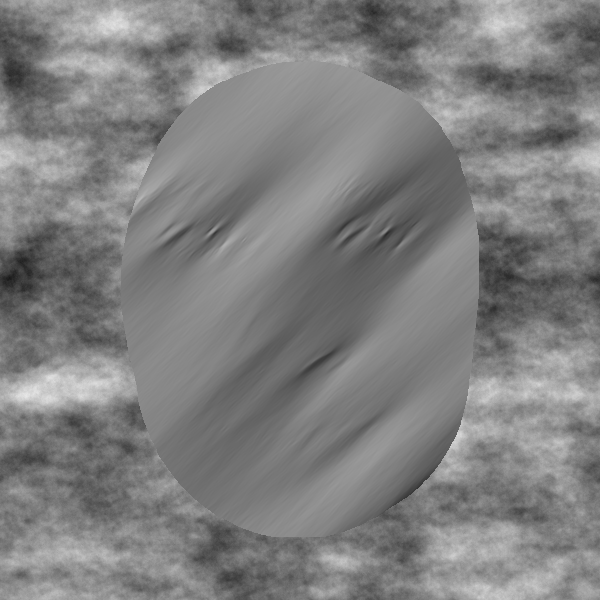

Supplement: S3 File — (ZIP) [file pone.0229185.s003.zip › eq_m04_a.bmp_135.bmp]

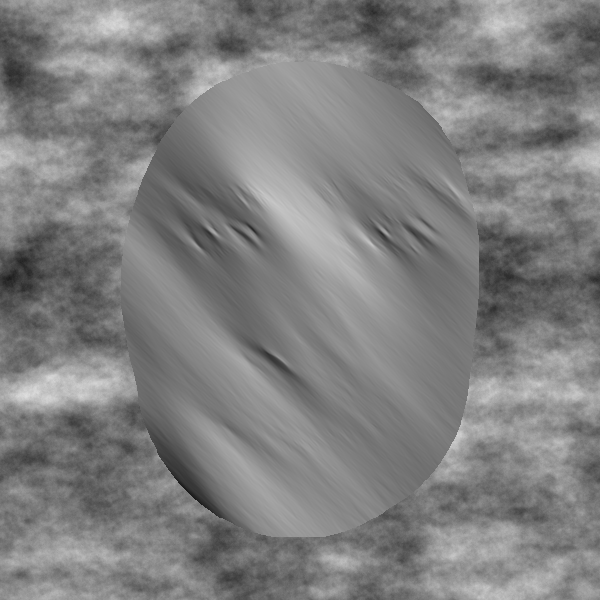

Supplement: S3 File — (ZIP) [file pone.0229185.s003.zip › eq_m04_a.bmp_45.bmp]

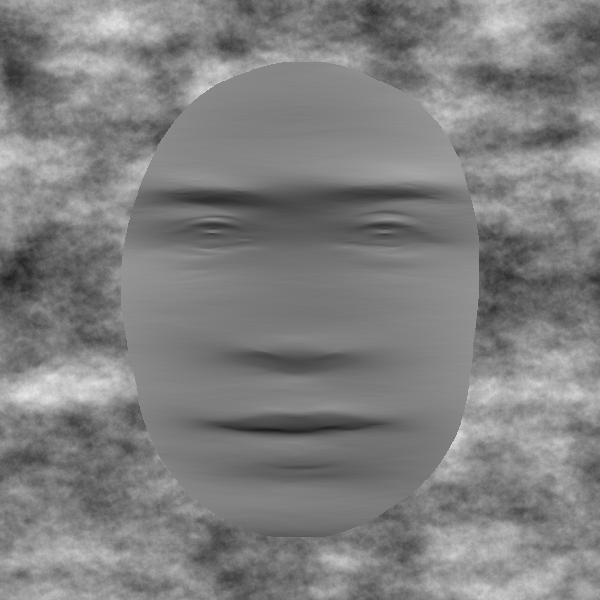

Supplement: S3 File — (ZIP) [file pone.0229185.s003.zip › eq_m04_a.bmp_90.bmp]

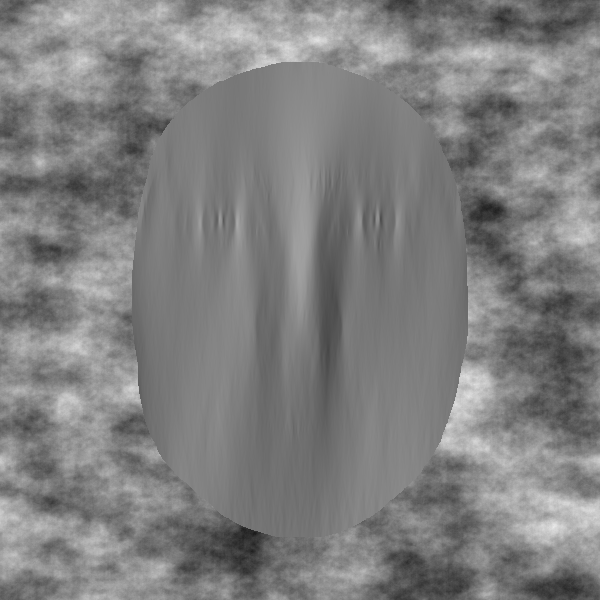

Supplement: S3 File — (ZIP) [file pone.0229185.s003.zip › eq_m05_a.bmp_0.bmp]

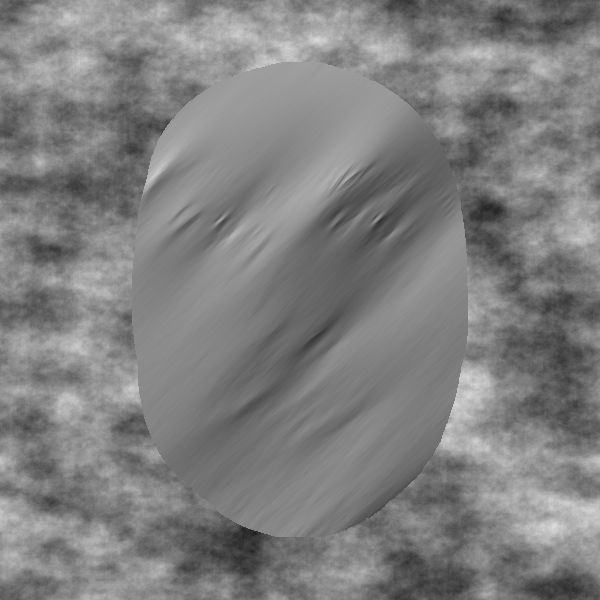

Supplement: S3 File — (ZIP) [file pone.0229185.s003.zip › eq_m05_a.bmp_135.bmp]

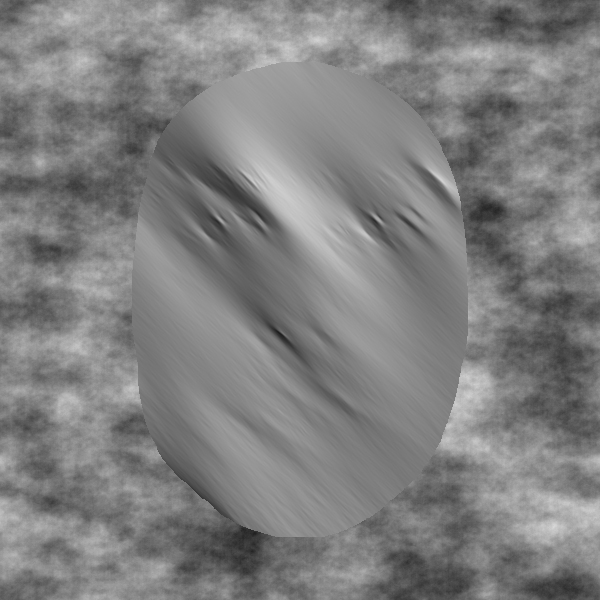

Supplement: S3 File — (ZIP) [file pone.0229185.s003.zip › eq_m05_a.bmp_45.bmp]

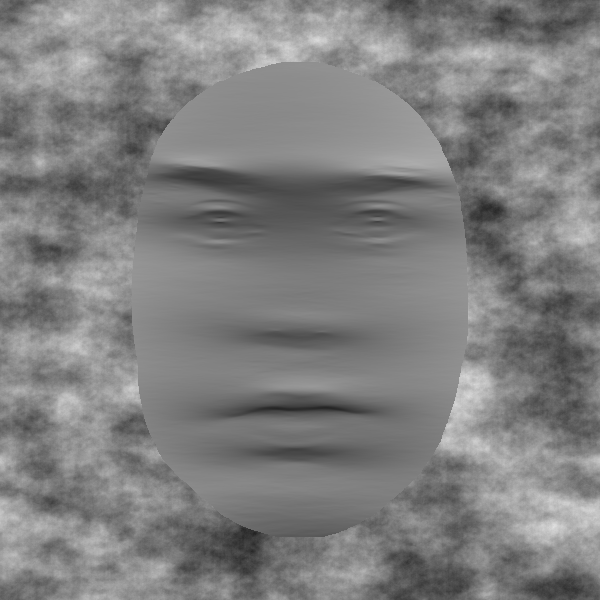

Supplement: S3 File — (ZIP) [file pone.0229185.s003.zip › eq_m05_a.bmp_90.bmp]

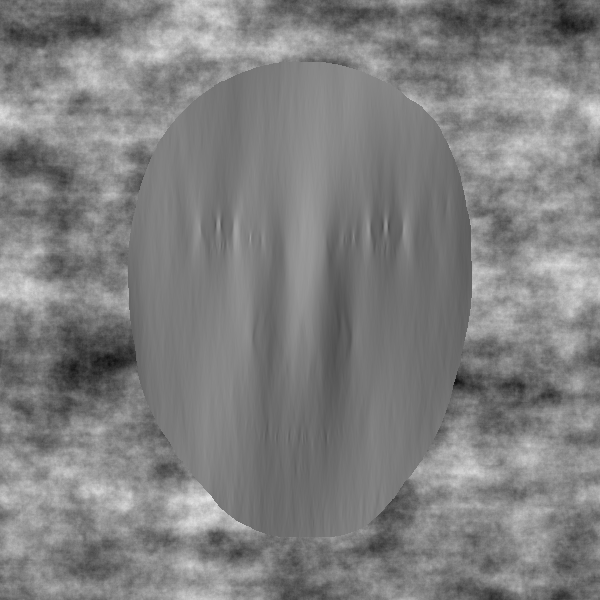

Supplement: S3 File — (ZIP) [file pone.0229185.s003.zip › eq_m06_a.bmp_0.bmp]

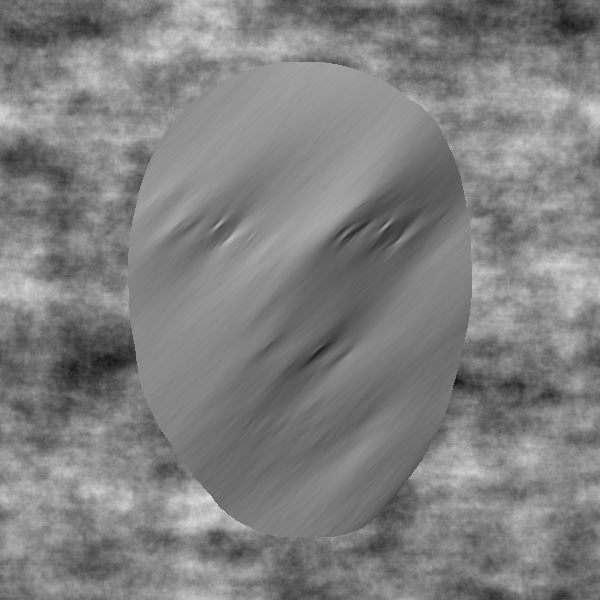

Supplement: S3 File — (ZIP) [file pone.0229185.s003.zip › eq_m06_a.bmp_135.bmp]

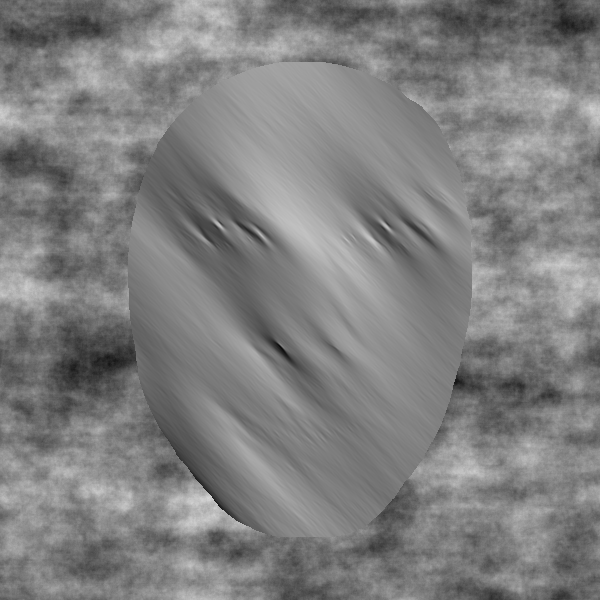

Supplement: S3 File — (ZIP) [file pone.0229185.s003.zip › eq_m06_a.bmp_45.bmp]

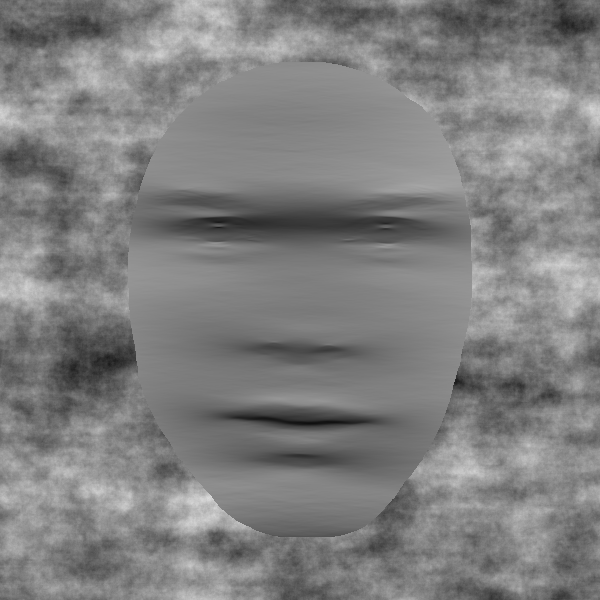

Supplement: S3 File — (ZIP) [file pone.0229185.s003.zip › eq_m06_a.bmp_90.bmp]

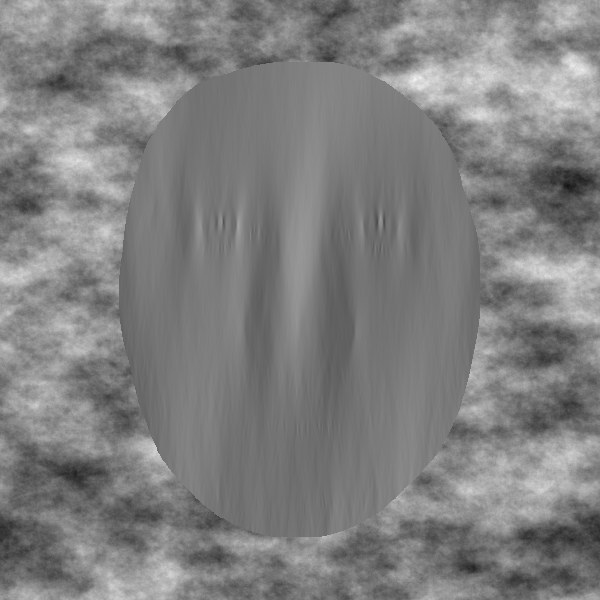

Supplement: S3 File — (ZIP) [file pone.0229185.s003.zip › eq_m07_a.bmp_0.bmp]

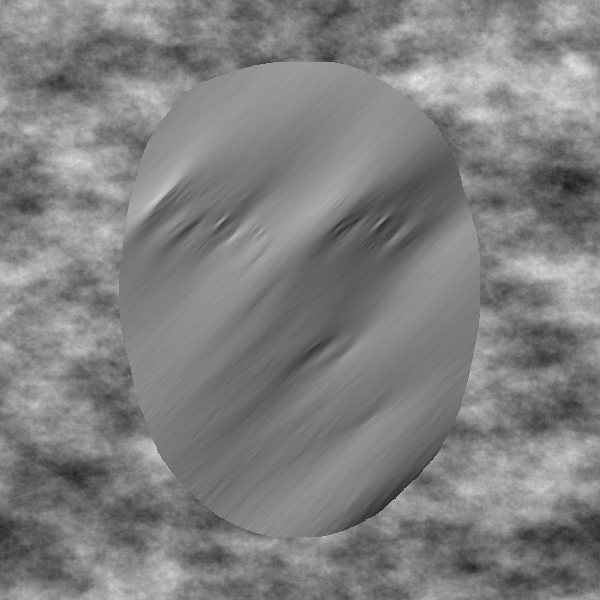

Supplement: S3 File — (ZIP) [file pone.0229185.s003.zip › eq_m07_a.bmp_135.bmp]

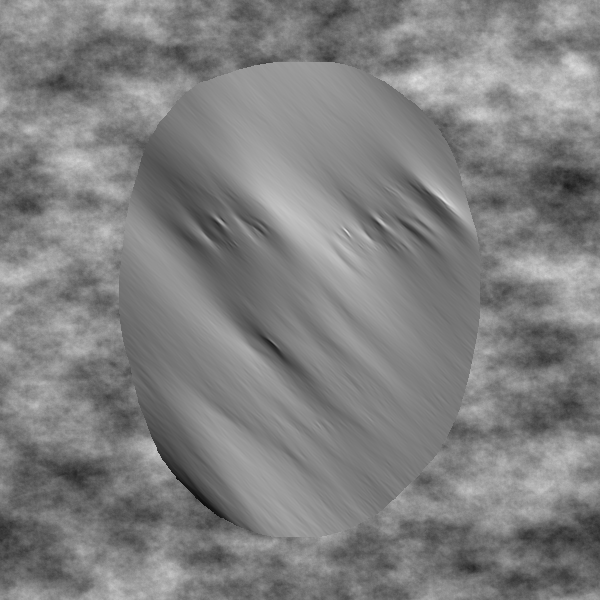

Supplement: S3 File — (ZIP) [file pone.0229185.s003.zip › eq_m07_a.bmp_45.bmp]

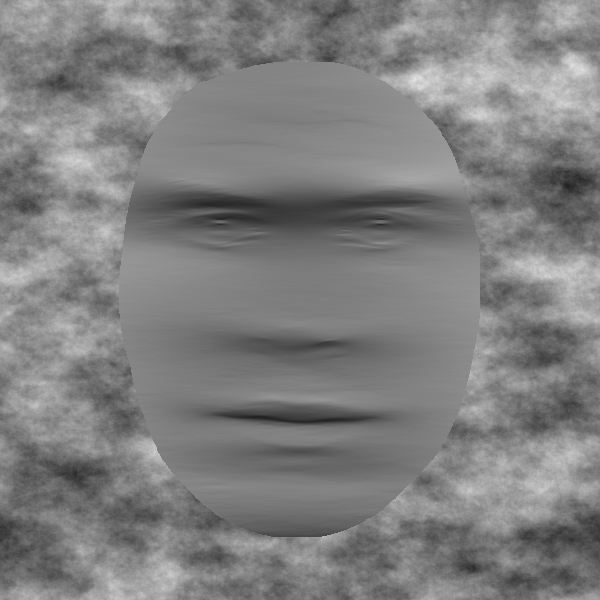

Supplement: S3 File — (ZIP) [file pone.0229185.s003.zip › eq_m07_a.bmp_90.bmp]

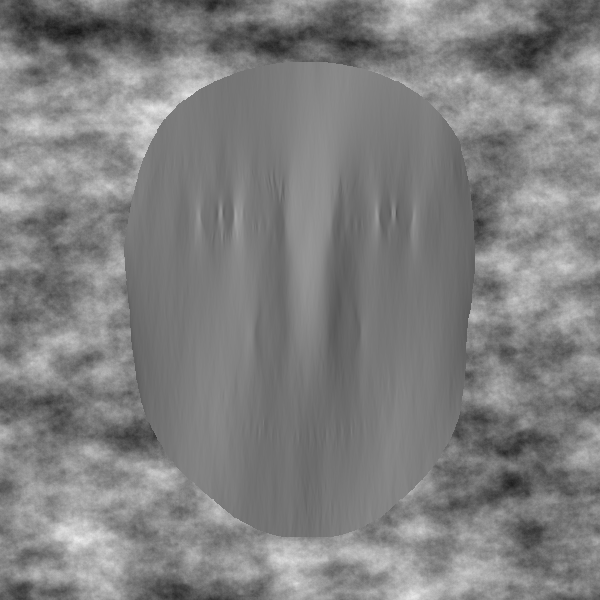

Supplement: S3 File — (ZIP) [file pone.0229185.s003.zip › eq_m08_a.bmp_0.bmp]

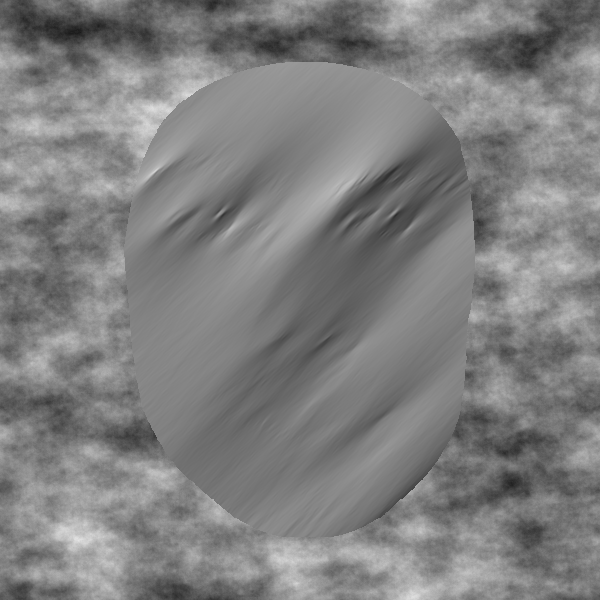

Supplement: S3 File — (ZIP) [file pone.0229185.s003.zip › eq_m08_a.bmp_135.bmp]

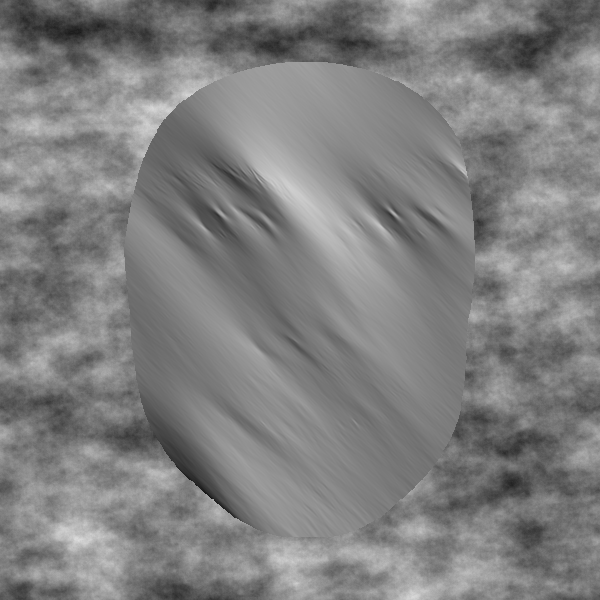

Supplement: S3 File — (ZIP) [file pone.0229185.s003.zip › eq_m08_a.bmp_45.bmp]

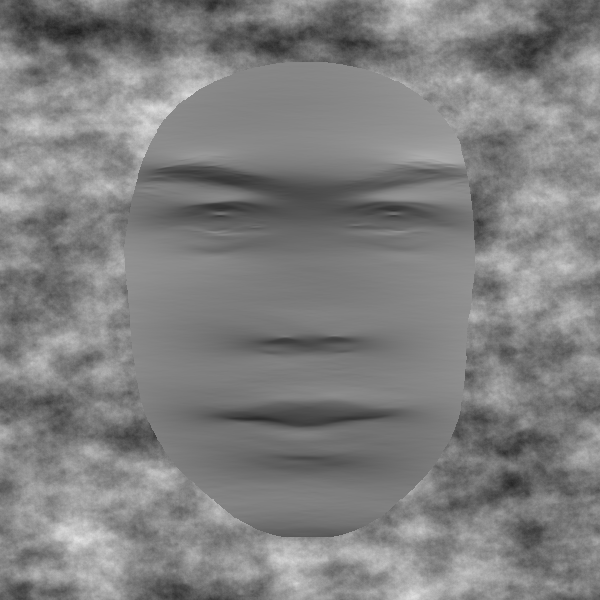

Supplement: S3 File — (ZIP) [file pone.0229185.s003.zip › eq_m08_a.bmp_90.bmp]

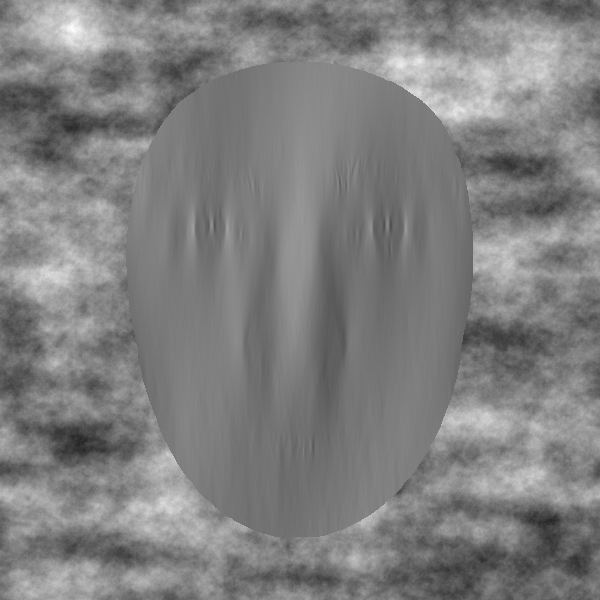

Supplement: S3 File — (ZIP) [file pone.0229185.s003.zip › eq_m09_a.bmp_0.bmp]

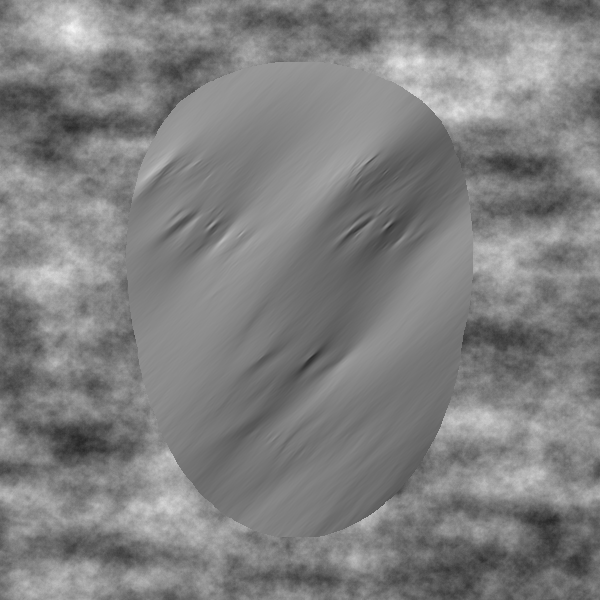

Supplement: S3 File — (ZIP) [file pone.0229185.s003.zip › eq_m09_a.bmp_135.bmp]

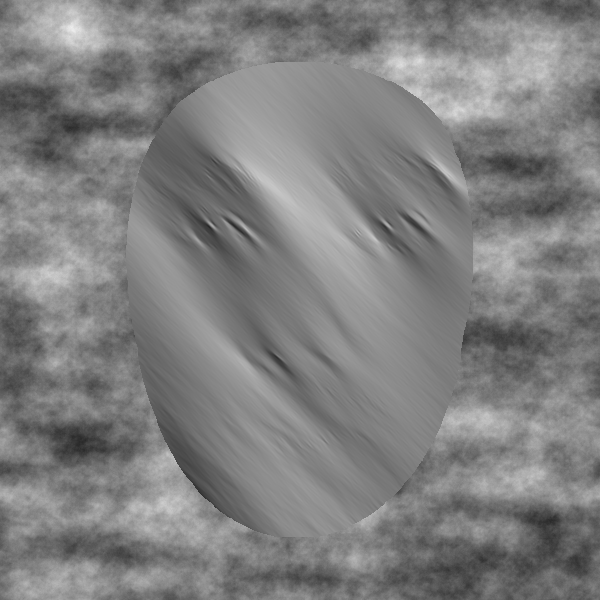

Supplement: S3 File — (ZIP) [file pone.0229185.s003.zip › eq_m09_a.bmp_45.bmp]

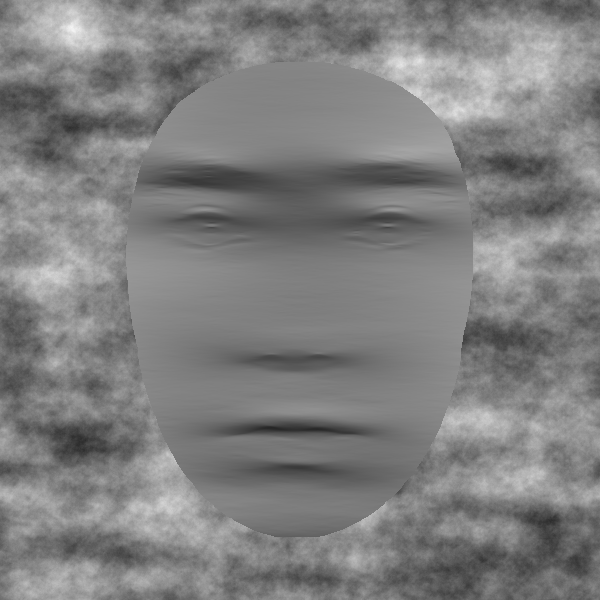

Supplement: S3 File — (ZIP) [file pone.0229185.s003.zip › eq_m09_a.bmp_90.bmp]

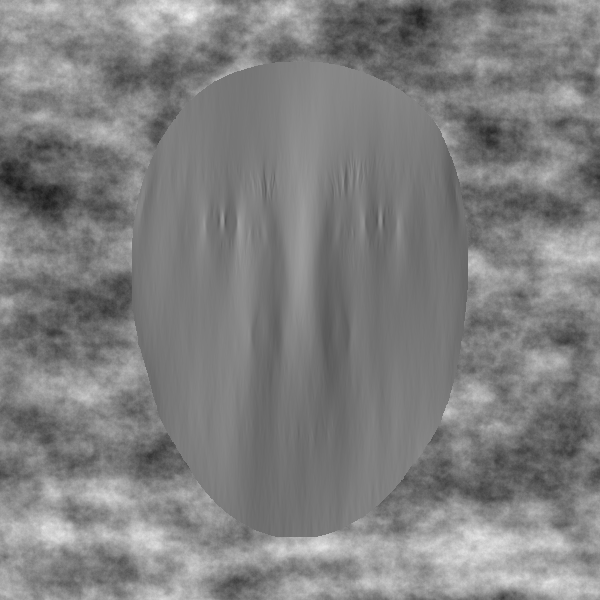

Supplement: S3 File — (ZIP) [file pone.0229185.s003.zip › eq_m10_a.bmp_0.bmp]

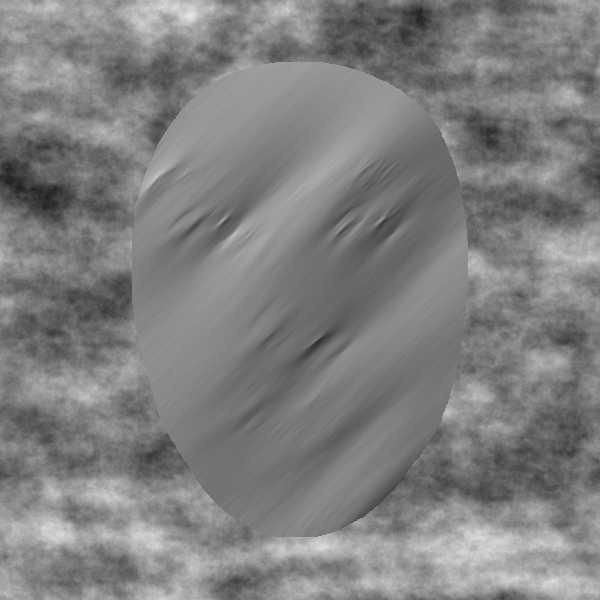

Supplement: S3 File — (ZIP) [file pone.0229185.s003.zip › eq_m10_a.bmp_135.bmp]

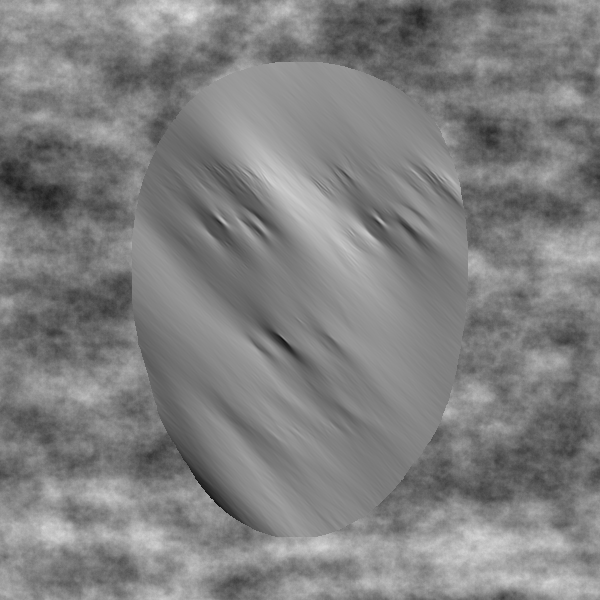

Supplement: S3 File — (ZIP) [file pone.0229185.s003.zip › eq_m10_a.bmp_45.bmp]

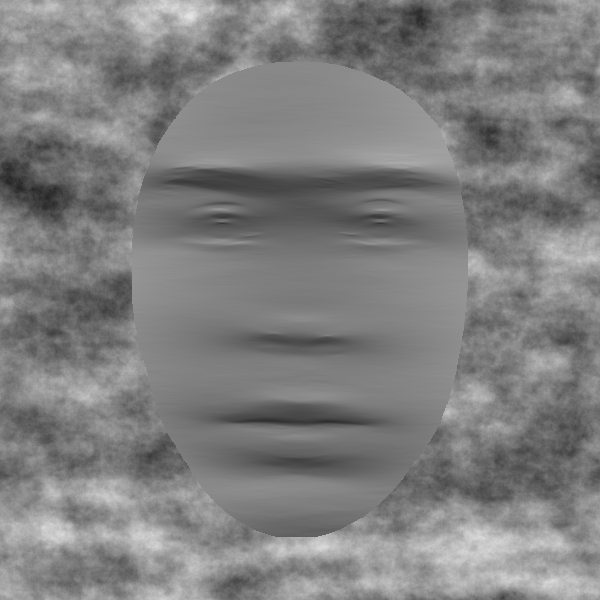

Supplement: S3 File — (ZIP) [file pone.0229185.s003.zip › eq_m10_a.bmp_90.bmp]

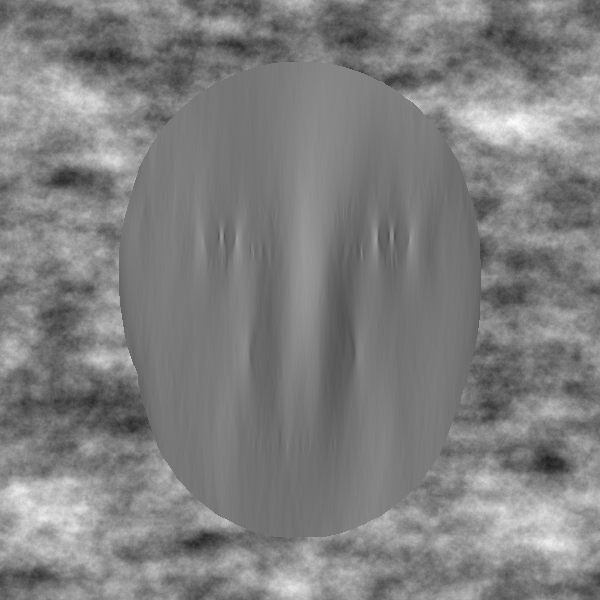

Supplement: S3 File — (ZIP) [file pone.0229185.s003.zip › eq_m11_a.bmp_0.bmp]

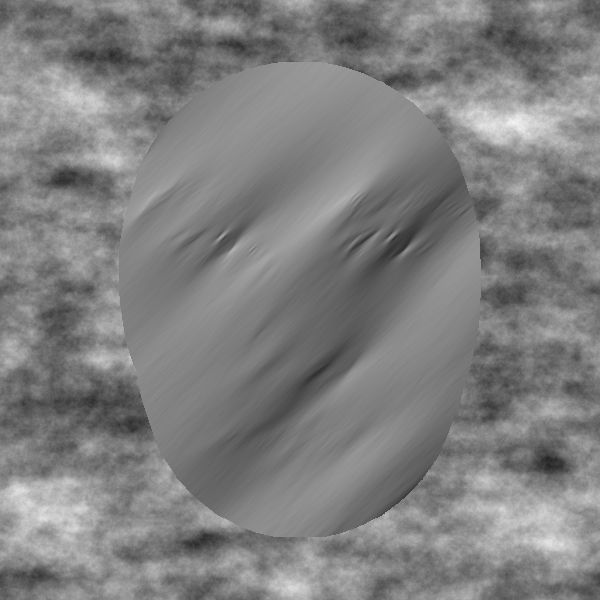

Supplement: S3 File — (ZIP) [file pone.0229185.s003.zip › eq_m11_a.bmp_135.bmp]

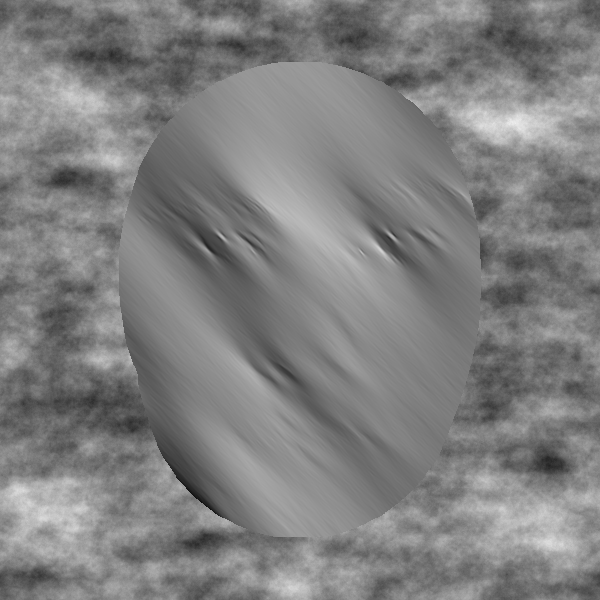

Supplement: S3 File — (ZIP) [file pone.0229185.s003.zip › eq_m11_a.bmp_45.bmp]

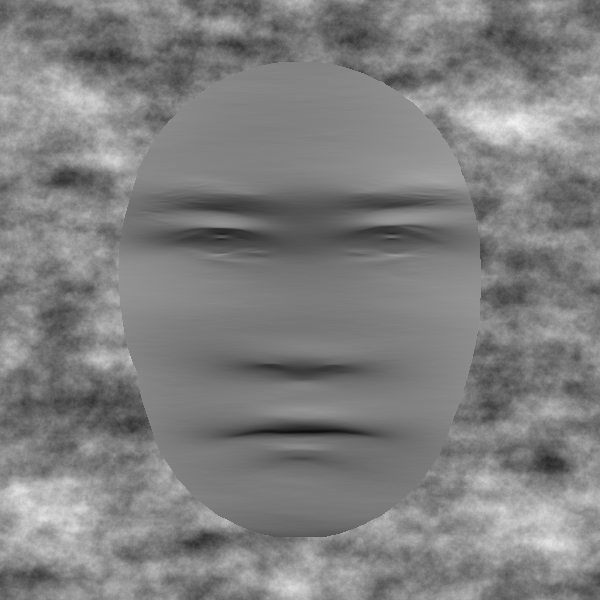

Supplement: S3 File — (ZIP) [file pone.0229185.s003.zip › eq_m11_a.bmp_90.bmp]

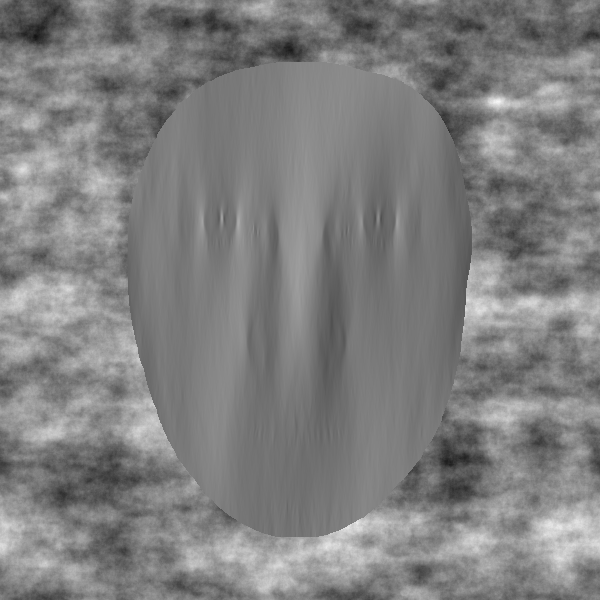

Supplement: S3 File — (ZIP) [file pone.0229185.s003.zip › eq_m13_a.bmp_0.bmp]

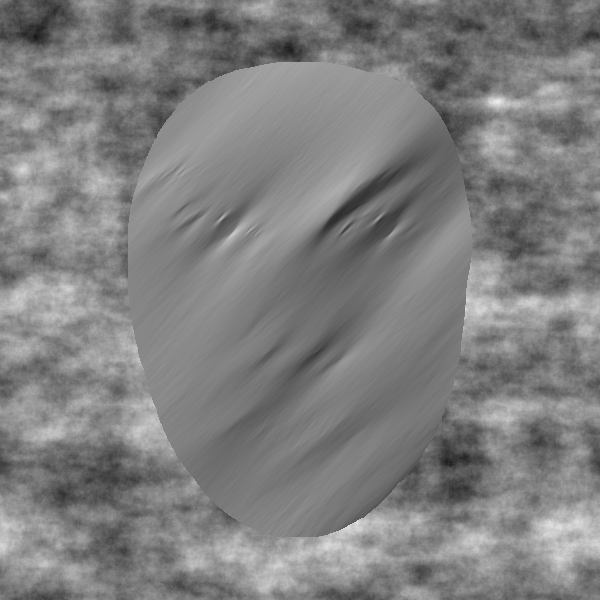

Supplement: S3 File — (ZIP) [file pone.0229185.s003.zip › eq_m13_a.bmp_135.bmp]

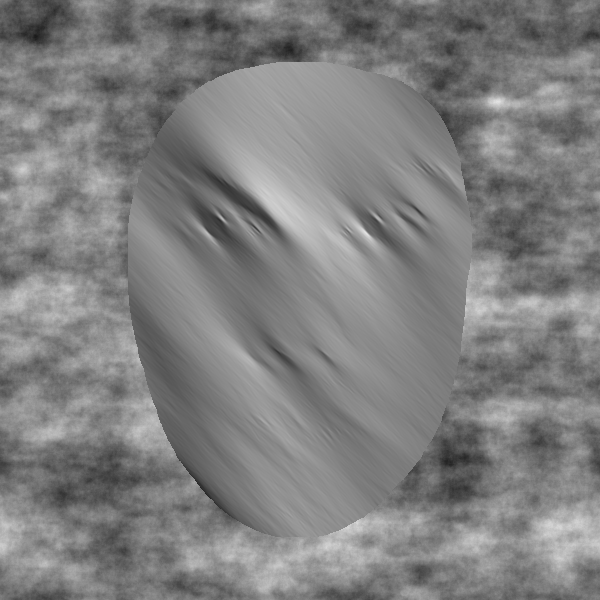

Supplement: S3 File — (ZIP) [file pone.0229185.s003.zip › eq_m13_a.bmp_45.bmp]

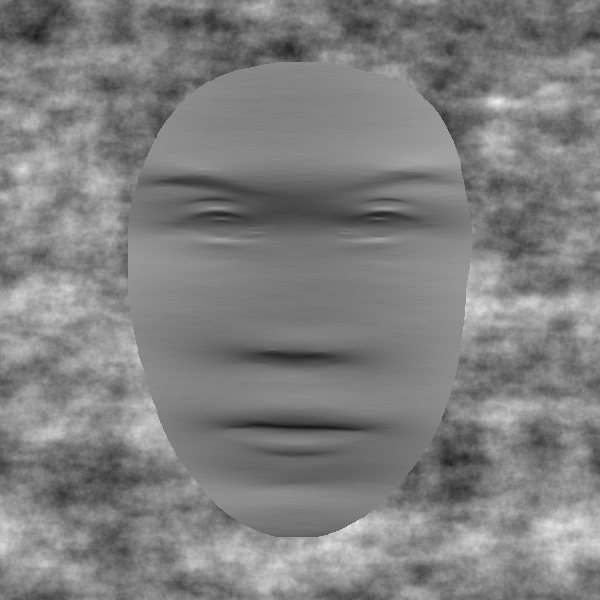

Supplement: S3 File — (ZIP) [file pone.0229185.s003.zip › eq_m13_a.bmp_90.bmp]
